# Phosphomethylpyrimidine Synthase (ThiC): Trapping of Five Intermediates Provides Mechanistic Insights on a Complex Radical Cascade Reaction in Thiamin Biosynthesis

**DOI:** 10.1021/acscentsci.4c00125

**Published:** 2024-05-13

**Authors:** Vishav Sharma, Dmytro Fedoseyenko, Sumedh Joshi, Sameh Abdelwahed, Tadhg P. Begley

**Affiliations:** Department of Chemistry, Texas A&M University, College Station, Texas 77842, United States

## Abstract

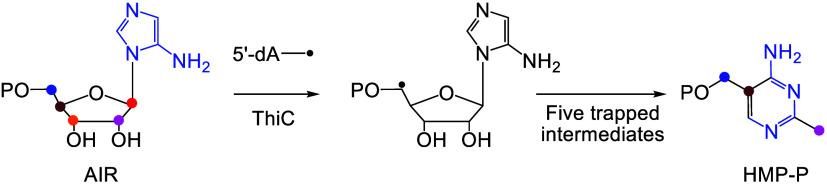

Phosphomethylpyrimidine synthase (ThiC) catalyzes the conversion of AIR to the thiamin pyrimidine HMP-P. This reaction is the most complex enzyme-catalyzed radical cascade identified to date, and the detailed mechanism has remained elusive. In this paper, we describe the trapping of five new intermediates that provide snapshots of the ThiC reaction coordinate and enable the formulation of a revised mechanism for the ThiC-catalyzed reaction.

## Abstract 2

The mechanism of thiamin pyrimidine formation is the last major unsolved problem in thiamin biosynthesis. Here, we describe the trapping of five intermediates, providing snapshots of the reaction coordinate for this complex radical rearrangement.

## Introduction

**Figure 1:**
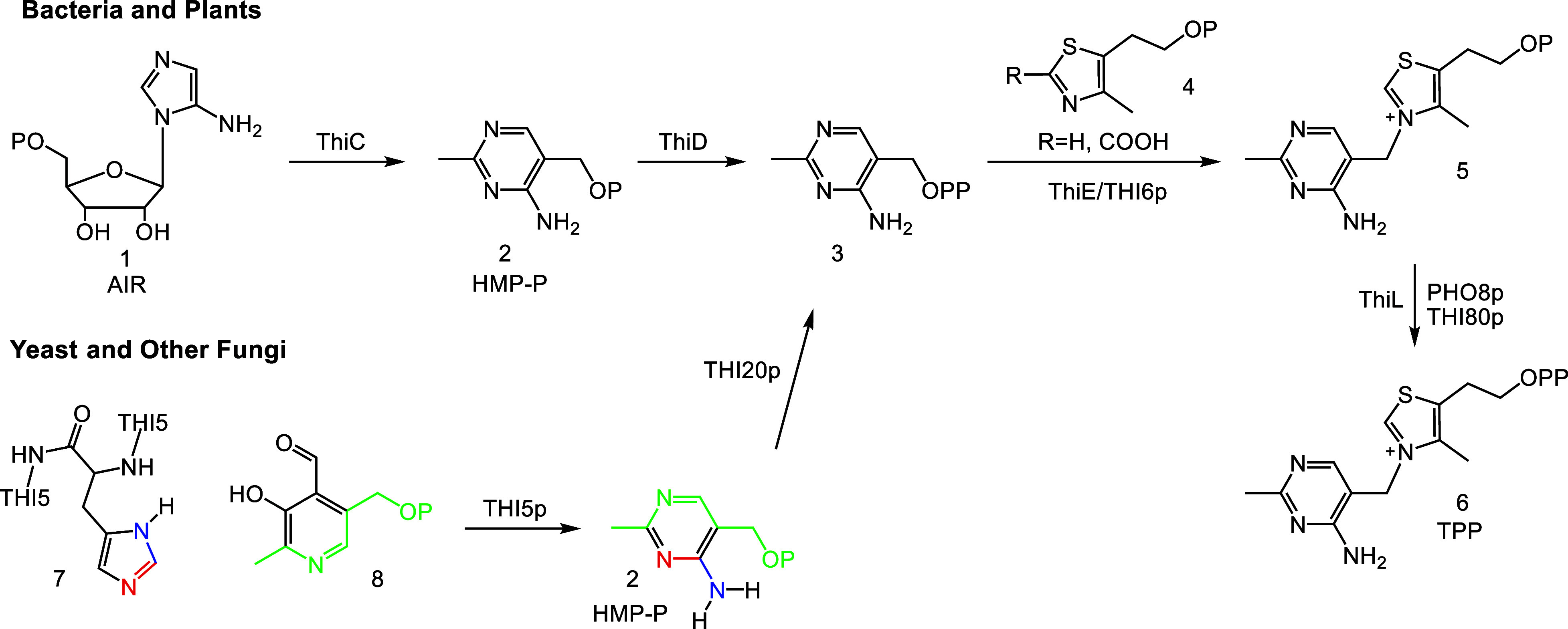
Outline of thiamin biosynthesis.

The thiamin pyrophosphate (TPP, **6**) biosynthetic pathway in bacteria and plants involves the separate biosynthesis of the 4-methyl-5-hydroxyethylthiazole phosphate (thiamin thiazole, **4**) and 4-amino-5-hydroxymethyl-2-methyl pyrimidine phosphate (thiamin pyrimidine, HMP-P, **2**) heterocycles.^1,2^ These heterocycles are then coupled to form thiamin monophosphate **5** which, upon phosphorylation, gives TPP, an essential cofactor in all domains of life (Figure 1). The mechanistic enzymology of thiamin thiazole biosynthesis in plants and the three microbial kingdoms has been studied in detail, and two different strategies for the biosynthesis of thiamin pyrimidine have been identified.^3−9^ In yeast and other fungi, the pyrimidine is formed from PLP **8** and an active site histidine residue of the THI5 protein **7**, while in bacteria and plants, the pyrimidine is formed from 5-aminoimidazole ribotide (AIR, **1**), an intermediate in purine biosynthesis.^10−14^

**Figure 2:**
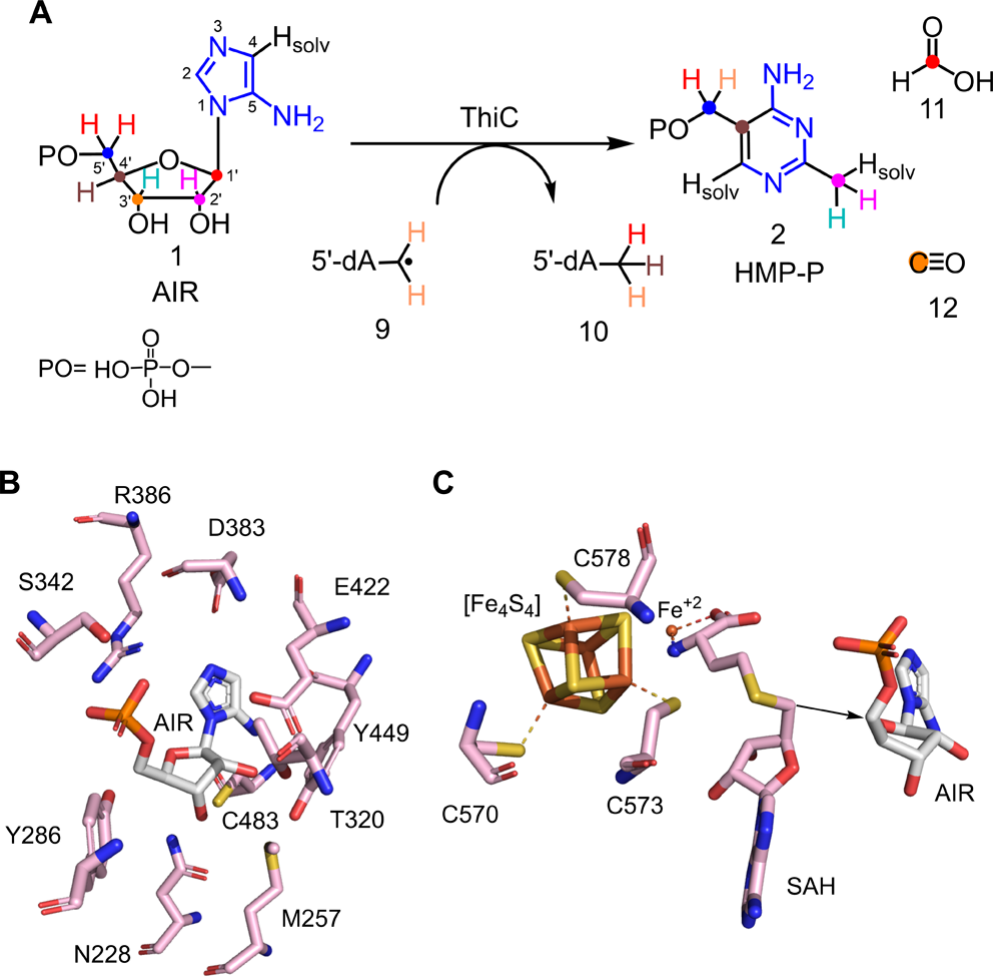
(A) The ThiC-catalyzed reaction with the fate of all atoms shown in color. (B) Active site architecture of *At*ThiC showing residues involved in AIR binding (PDB: 4S28). (C) Active site of *At*ThiC showing the noncanonical [Fe_4_S_4_] cluster (CX_2_CX_4_C), a novel mononuclear iron site involved in SAM binding and bound S-adenosyl homocysteine (SAH).

**Figure 3:**
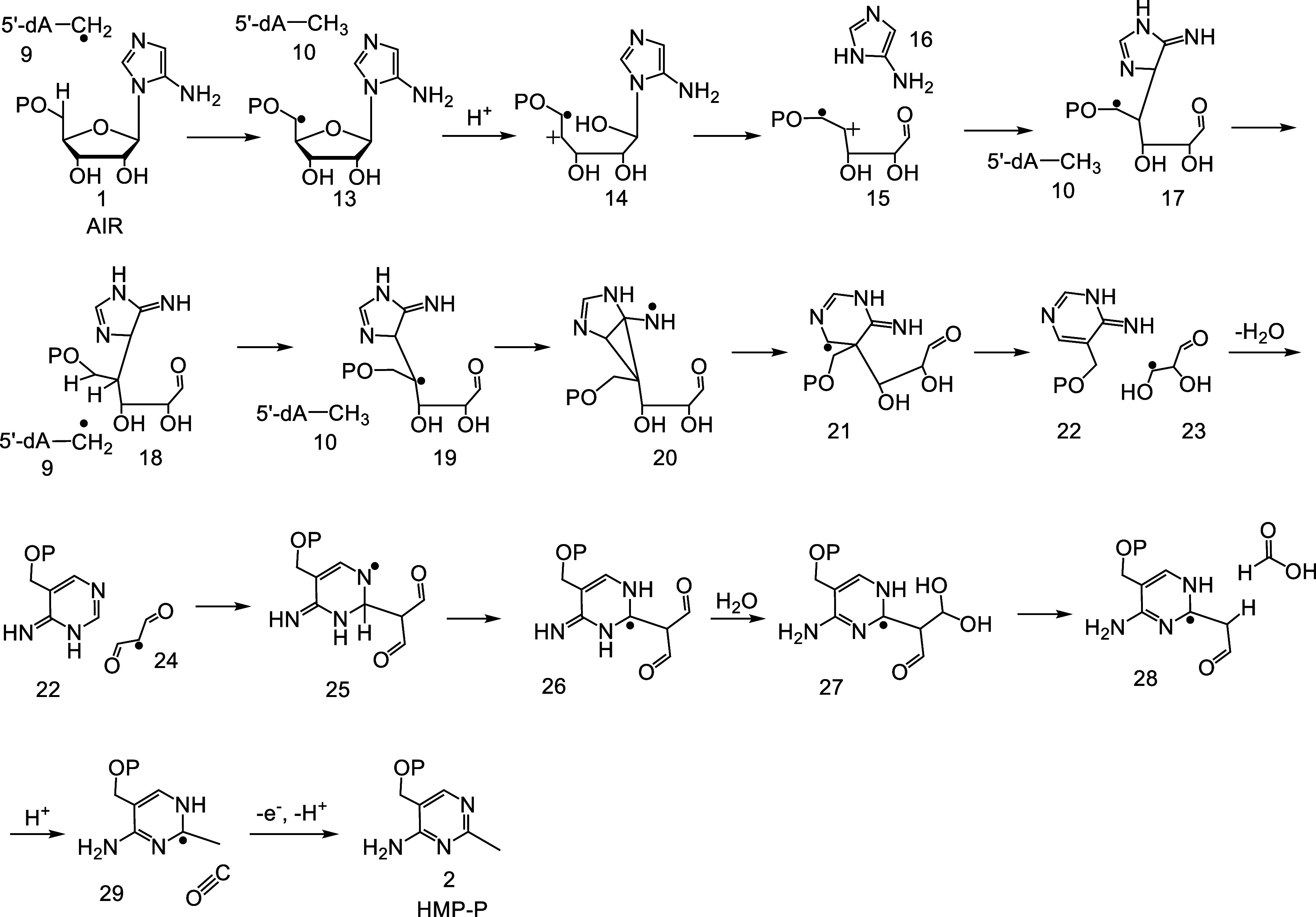
Preliminary mechanistic proposal for the ThiC-catalyzed conversion of AIR (**1**) to HMP-P (**2**).

The complex rearrangement of AIR to HMP-P, formate, and carbon monoxide is catalyzed by the radical *S*-adenosylmethionine (SAM) enzyme phosphomethylpyrimidine synthase (ThiC, Figure 2a).^13,15^ A comprehensive set of labeling studies using ^2^H and ^13^C-AIR isotopologues has established the atom transfer pattern shown.^16,17^ These studies demonstrated that the initial hydrogen atom abstraction occurs from C5′ of AIR and that all of the aminoimidazole atoms are retained in the pyrimidine except for the imidazole C4 proton which readily exchanges with the buffer. They demonstrate that the C4′ carbon of AIR is inserted into the C4–C5 double bond of the heterocycle, converting the aminoimidazole to an aminopyrimidine, that the pyrimidine methyl group is derived from C2′, H2′, H3′ and a proton from the buffer, and that the carbons of formate and CO are derived from C1′ and C3′ respectively. These labeling experiments are consistent with the mechanistic proposal shown in Figure 3.^17^ In this proposal, the 5′-dA radical, generated at the active site by the reduction of SAM by the [Fe_4_S_4_]^+1^ cluster, abstracts a hydrogen atom from C5′ of AIR to generate **10** and the substrate radical **13**. **13** undergoes an acid-catalyzed ring opening to give the alkene radical cation **14**, followed by C–N bond cleavage to give **15** and **16**. Electrophilic addition of **15** to **16** gives **17**. Hydrogen atom transfer from **10** to **17** followed by hydrogen atom abstraction from C4′ of **18** gives radical **19**. The labeling studies support this novel 5′-dA radical mediated 1,2-hydrogen atom shift.^17,18^ A Beckwith ring expansion gives **21**.^19^ A β-scission reaction gives **22** and **23**. Another β-scission reaction, analogous to reactions catalyzed by ribonucleotide reductase and diol dehydratase,^20,21^ converts **23** to **24**, which then adds to the pyrimidine to give **25**. Tautomerization (β-scission) of **25** gives **26**.^22,23^ Aldehyde hydration and loss of formate followed by protonation gives **28**. Loss of carbon monoxide by a final β-scission reaction, followed by deprotonation and electron transfer back to the initially oxidized [Fe_4_S_4_]^+2^ cluster, gives HMP-P. To the best of our knowledge, the radical cascade catalyzed by ThiC is the most complex unsolved radical rearrangement in primary metabolism.

The active site structure of the *Arabidopsis thaliana* ThiC (*At*ThiC, PDB ID: 4S28), showing the AIR binding site, the noncanonical [Fe_4_S_4_] cluster (CX_2_CX_4_C), a novel mononuclear iron site involved in SAM binding and bound S-adenosyl homocysteine (SAH) is shown in Figures 2B and 2C.^24,25^ This structure is consistent with the 5′-dA radical **9** abstracting a hydrogen atom from C5′ of AIR to give **13** but does not provide information on how this radical rearranges to form HMP-P. The structure of the *Caulobacter crescentus* ThiC (*Cc*ThiC, PDB ID: 3EPN) is similarly uninformative on the mechanistic details of the rearrangement reaction.^13^

ThiC is a challenging experimental system. In addition to enzyme instability, optimized enzyme preparations yielded a maximum of 0.3 mol of product/mol of enzyme. We also found that the enzyme is highly specific for the AIR structure and that most AIR analogs were not substrates. Systematic mutagenesis of the active site residues failed to identify trapped intermediates by the HPLC analysis of reaction mixtures. These properties precluded many of the strategies previously used to elucidate the mechanism of radical SAM enzymes and required the development of alternative approaches. In this paper, we describe the trapping and characterization of five intermediates that provide snapshots of the ThiC reaction coordinate and use this new information to revise our initial mechanistic proposal for the ThiC-catalyzed reaction.

## Results

### Conversion of AIR to HMP-P Is Inefficient (28%)

The level of formation of HMP-P was consistently lower than the level of consumption of AIR in the ThiC-catalyzed reaction. For example, when 300 μM of *At*ThiC was incubated with 410 μM AIR, 1 mM SAM, and 6 mM titanium(III) citrate, 250 μM of AIR was consumed while only 70 μM of HMP-P was formed. This observation suggested that in addition to the premature quenching of the 5′-dA radical, other quenched radical intermediates might also escape from the ThiC active site. The structures of such trapped intermediates might provide insight into the reaction mechanism by making available snapshots of the reaction coordinate. Our initial attempts to detect such shunt products by HPLC analysis of the ThiC reaction mixtures were unsuccessful, most likely due to the lack of a chromophore on the expected ribose fragments as well as the reactivity of aminoimidazole with oxygen.^26^

We systematically tested various chromophoric derivatizing reagents to identify nonchromophoric shunt products. While we successfully detected trace levels of sulfur-containing compounds, their characterization proved elusive due to product instability. These products appeared to be generated following the reaction of the ThiC shunt products with the byproducts of sodium dithionite (SO_2_ radical anion, bisulfite, and thiosulfate),^27,28^ which was used as a reducing agent. Finally, the trapping of ThiC shunt products was achieved by replacing dithionite with titanium(III) citrate.

### Use of Pentafluorobenzyl Hydroxylamine (PFBHA) to Facilitate the Detection and Characterization of ThiC Shunt Products

**Figure 4:**
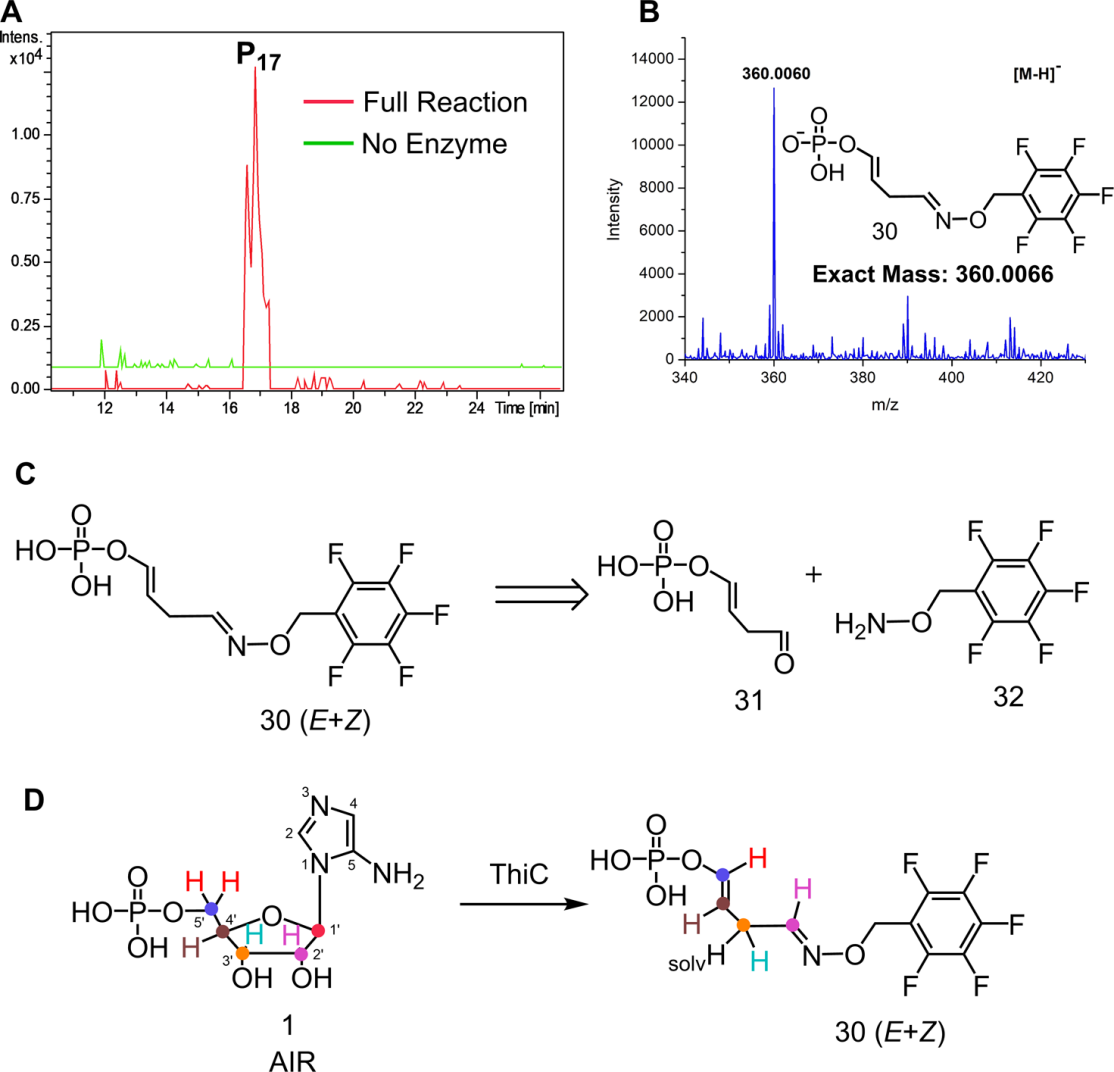
(A) Extracted Ion Chromatogram for the new product P_17_ in the ThiC-catalyzed reaction. The two peaks observed for P_17_ are the *E* and *Z* isomers of PFBHA-oxime **30**. (B) MS analysis of P_17_. (C) Retrosynthetic analysis of **30**. (D) Summary of labeling studies to determine the origin of the atoms of compound **30**.

Radical damage to DNA and the reactivity of nucleic acid radicals have been extensively studied,^29−31^ and in most cases, it results in the formation of aldehyde-containing sugar fragments. This prompted us to explore the use of *O*-(2,3,4,5,6-pentafluorobenzyl) hydroxylamine (PFBHA, **32**) to facilitate the detection and characterization of ThiC shunt products. PFBHA is an aldehyde derivatizing reagent, and the resulting oximes can be detected with high sensitivity by LC-MS as a mixture of *E* and *Z* isomers.^32^ In the event, LC-MS analysis of an *At*ThiC reaction mixture treated with PFBHA resulted in the detection of a new peak eluting at 17 min (P_17_, Figures 4A and S3). MS analysis of P_17_ was consistent with the structure of the PFBHA-oxime **30** formed by the derivatization of aldehyde **31** (Figures 4B,C and S4). The structure of **30** was confirmed by ^1^H and ^31^P NMR analysis using *At*ThiC (D383A), which produced larger quantities of **30** than the native enzyme (see below and Figures S5, S6 and S7). The derivatization experiment was repeated using ^2^H and ^13^C labeled AIR to determine the origin of the atoms of **30** (Figure 4D). This isotopologue analysis demonstrated that the four carbons of **30** are derived from the 2′, 3′, 4′, and 5′ carbons of AIR. The C1′ and the atoms from the aminoimidazole of AIR are not incorporated into **30**. Of the seven hydrogens in **30**, four are derived from the 2′, 3′, 4′, and 5′ protons of AIR, two are derived from PFBHA, and one proton is incorporated from the solvent (Figure S8). The exchange of the 3′H with solvent suggests that C3′ is alpha to the carbonyl of **31** (Figure S8E). The retention of the C4′-H (abstracted by **9** in the native reaction, **18** to **19**, Figure 3) and C3′ (lost as CO in the native reaction, **28** to **29**, Figure 3) in **30** suggests that shunt product **31** is formed before abstraction of the C4′-H and before CO formation. The absence of C1′ in **30** demonstrates that the C1′-C2′ bond of AIR is cleaved in the early stages of the ThiC-catalyzed reaction.

**Figure 5:**
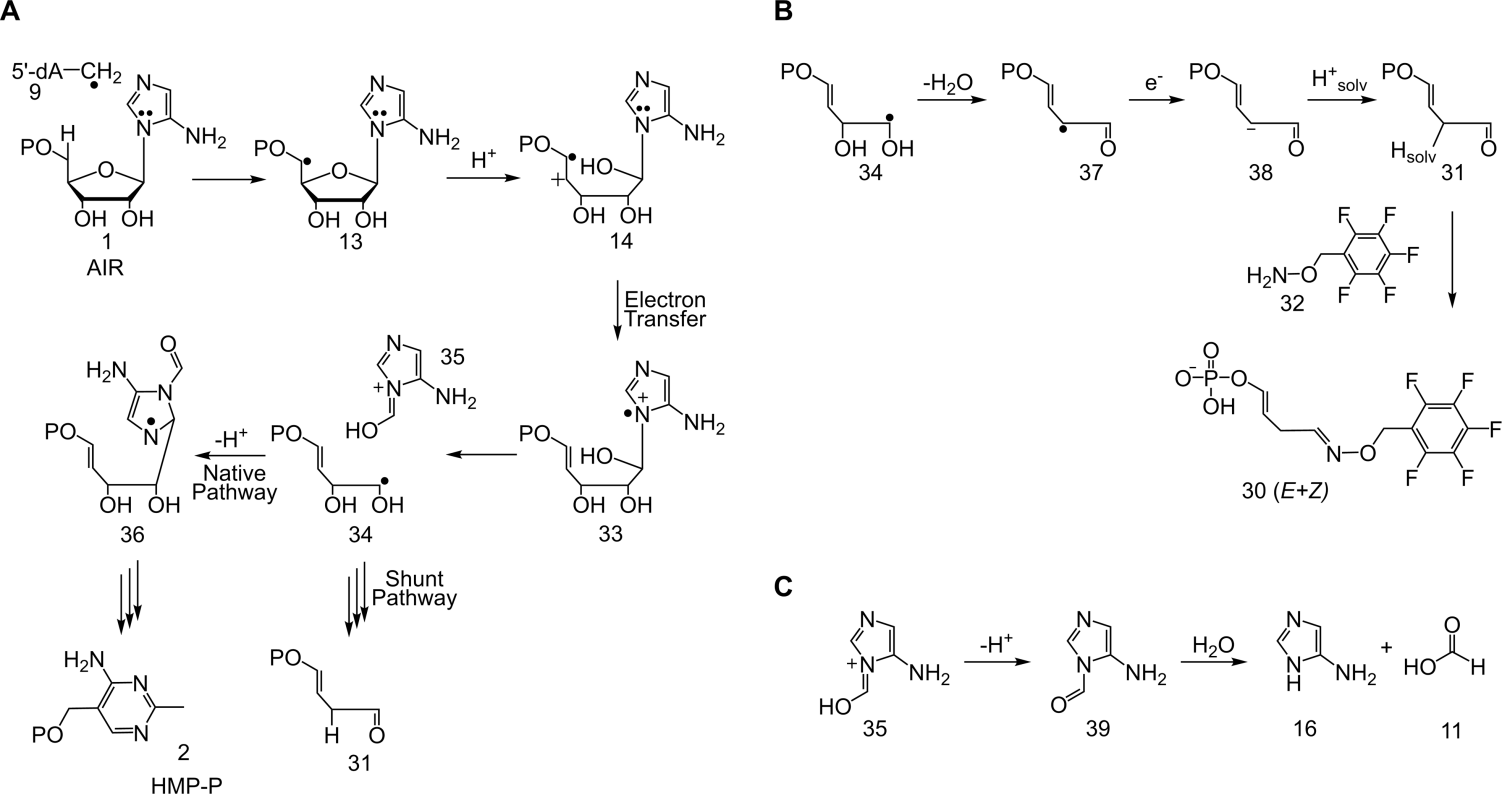
(A) Revised mechanistic proposal for the early steps of the ThiC-catalyzed reaction. (B) Mechanistic proposal for the formation of shunt metabolite **31**. (C) Proposed scheme for the hydrolysis of **35** in the shunt pathway.

These data are not consistent with the ThiC mechanism proposed in Figure 3.^17^ Specifically, that mechanism predicts that cleavage of the C1′-C2′ bond occurs after recombination of the imidazole and the ribose fragments (**15** + **16**), while the isolation of **30** suggests that cleavage of the C1′-C2′ bond occurs prior to the attachment of the ribose to the aminoimidazole. A revised proposal for the early steps of the ThiC mechanism, consistent with these observations, is shown in Figure 5A. In this mechanism, the 5′-dA radical abstracts a hydrogen atom from C5′ of AIR to give **13**. Acid-catalyzed ring opening of **13** followed by electron transfer from the electron-rich aminoimidazole to the alkene radical cation gives **33**, which undergoes a β-scission to give **34** and **35**.^33−35^ Addition of radical **34** to **35** yields **36**, which is converted to HMP-P by a variant of the chemistry shown in Figure 3 (see Figure 11 for the conversion of **36** to HMP-P). In competition with the formation of **36**, we propose that **34** undergoes a diol dehydratase like reaction to give **37**, which is then reduced by the titanium(III) citrate or the [Fe_4_S_4_]^+1^ cluster to give **38**. Protonation of **38** by solvent or a solvent-exchangeable protein residue yields **31**, derivatized by PFBHA as **30** (Figure 5B). This modified proposal is consistent with prior EPR studies on hydroxybenzimidazole synthase (BzaF), an enzyme that catalyzes the conversion of AIR to 5-hydroxybenzimidazole during the anaerobic biosynthesis of vitamin B_12_.^36^

### Studies on *At*ThiC (D383A) and *At*ThiC (D383N)

The successful trapping and characterization of a ribose fragment in the native ThiC-catalyzed reaction and the resulting revised mechanistic proposal prompted us to revisit our analysis of reaction mixtures prepared using ThiC mutants. All of the residues mutated in this study are highly conserved across ThiC orthologs. Asp 383 is hydrogen bonded to N3 of AIR (Figures 2B and S9). Since the addition of nucleophilic radicals to heterocycles containing C=N bonds is acid-catalyzed,^37,38^ it is likely that protonation of the aminoimidazole N3 by the conserved Asp 383 activates the heterocycle for the addition of radical **34** to **35** (Figure 5A). We should therefore be able to change the ratio of **30** to HMP-P by mutating Asp 383. In the event, *At*ThiC (D383A) showed substrate consumption similar to the native *At*ThiC, but no HMP-P was detected in the reaction mixture (Figures S2, S10 and S11). Shunt product **30** was the only product detected by LC-MS analysis after derivatization with PFBHA (Figure S12). The **30**:5′-dA ratio was 1:9 (Figure S13). We could also detect the formation of formate from C1′ of AIR in the *At*ThiC (D383A) reaction using DEPT-90 NMR analysis consistent with the hydrolysis of **35** in the shunt pathway (Figures 5A, 5C and S14).

*At*ThiC (D383N) partially rescued the native activity (Figures S10 and S11) consistent with the weaker acidity of asparagine relative to aspartic acid. The *At*ThiC (D383A) reaction also gave the highest yield of shunt metabolite **30**, facilitating its characterization by NMR as described above (Figures S12C).

### Studies on *Cc*ThiC (E413Q)

**Figure 6:**
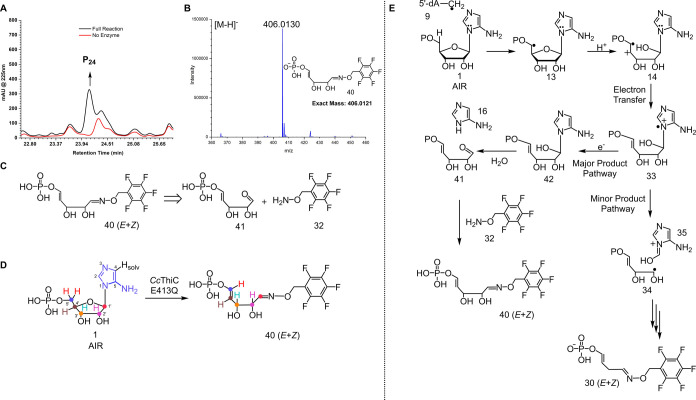
(A) HPLC analysis of the *Cc*ThiC (E413Q) catalyzed reaction after PFBHA treatment showing the formation of P_24_. (B) LC-MS analysis of P_24_. (C) Retrosynthetic analysis of compound **40**. (D) Summary of the labeling studies used to characterize **40** (*E* + *Z*). (E) Mechanistic proposal for the formation of **40** (*E* + *Z*).

Next, we turned our attention to the role of Glu 422 in *At*ThiC. This residue is hydrogen bonded to the C2′ hydroxyl of AIR (Figures 2B and S9) and is in close proximity to the aminoimidazole heterocycle. We used the corresponding glutamine mutant from the structurally characterized *Caulobacter crescentus* ThiC (*Cc*ThiC E413Q, PDB ID: 3EPN)^13^ for these studies because of the low solubility of *At*ThiC (E422Q). HPLC analysis of the *Cc*ThiC (E413Q) reaction mixture demonstrated AIR consumption but no formation of HMP-P (Figure S15). Instead, P_24_ (*E*+*Z*) and compound **30** were detected after derivatization with PFBHA. P_24_ had [M-H]^−^ of 406.013 Da (Figures 6A, 6B, S16). The exact mass and MS-MS analysis suggest that P_24_ has structure **40** and is derived from aldehyde **41** (Figures 6C and S17). The use of ^2^H, ^13^C, and ^15^N isotopologues of AIR confirmed that all five ribose carbons of AIR are present in **40** and that the aldehyde is at C1′. The aminoimidazole moiety is not present in the structure (Figures 6D and S18).

Our mechanistic proposal for the formation of **40** is shown in Figure 6E. Formation of **33** proceeds as shown in Figure 5A. We propose that hydrogen bonding of the E413 carboxylate with the C2′ hydroxyl is important in holding the C1′-C2′ bond in the stereoelectronically favored conformation for the β-scission reaction (i.e., perpendicular to the plane of the aminoimidazole). In addition, E413 likely provides electrostatic stabilization to the imidazolium radical in compound **33**. This stabilization retards electron transfer from the reduced cluster (or from excess reductant) to form **42** and allows time for the β -scission reaction to occur (**33** to **34** + **35**) in the native reaction. Both interactions are missing in the E413Q mutant, thus slowing down the β-scission and activating **33** for reduction to give **42**. *N*-Glycosyl bond cleavage of **42** would then give **16** and **41**. Detection of **40** as the major trapped shunt product supports the formation of radical cation intermediate **33** in the native reaction (Figure 6E). The minor product in the *Cc*ThiC (E413Q) reaction was confirmed as **30** based on LC-MS analysis (Figure S19). The formation of **30** indicates that the conversion of **33** to **34** + **35** is in competition with the conversion of **33** to **42** (Figure 6E).

Recently, we were able to overexpress *At*ThiC (E422Q) as a soluble protein and demonstrate that it gave the same products as *Cc*ThiC (E413Q) (Figure S20). We also detected low levels of shunt metabolite **40** in the native *Cc*ThiC reaction, supporting its relevance to the mechanism of the native reaction (Figure S21).

### Studies on *At*ThiC (N228D)

Asn228 and Glu422 of *At*ThiC are hydrogen bonded to the 3′ and 2′ OH of AIR, respectively, in a manner that is similar to the diol activation interactions in diol dehydratase and ribonucleotide reductase (PDB ID: 5I2G, 1DIO, 2CVW, Figures 2B and S9).^20,21,39−41^ This suggested that these two residues may catalyze the diol dehydratase reaction involved in the conversion of **75** to **60** (Figure 11). It also suggested that increasing the acidity of N228 by replacing it with an aspartic acid residue might activate a premature diol dehydratase reaction, enhancing the conversion of **34** to **31** (shunt pathway) and depleting **36**, the product of recombination of **34** and **35** (native pathway) (Figures 5A and 5B). In the event, *At*ThiC (N228D) showed substrate consumption similar to the native *At*ThiC, but no HMP-P formation was detected in the reaction mixture (Figure S22A). Shunt product **30** (*E* + *Z*) was the only product detected by LC-MS analysis after derivatization with PFBHA (Figures S22B and S22C).

### Trapping of Formyl Aminoimidazole (39)

**Figure 7:**
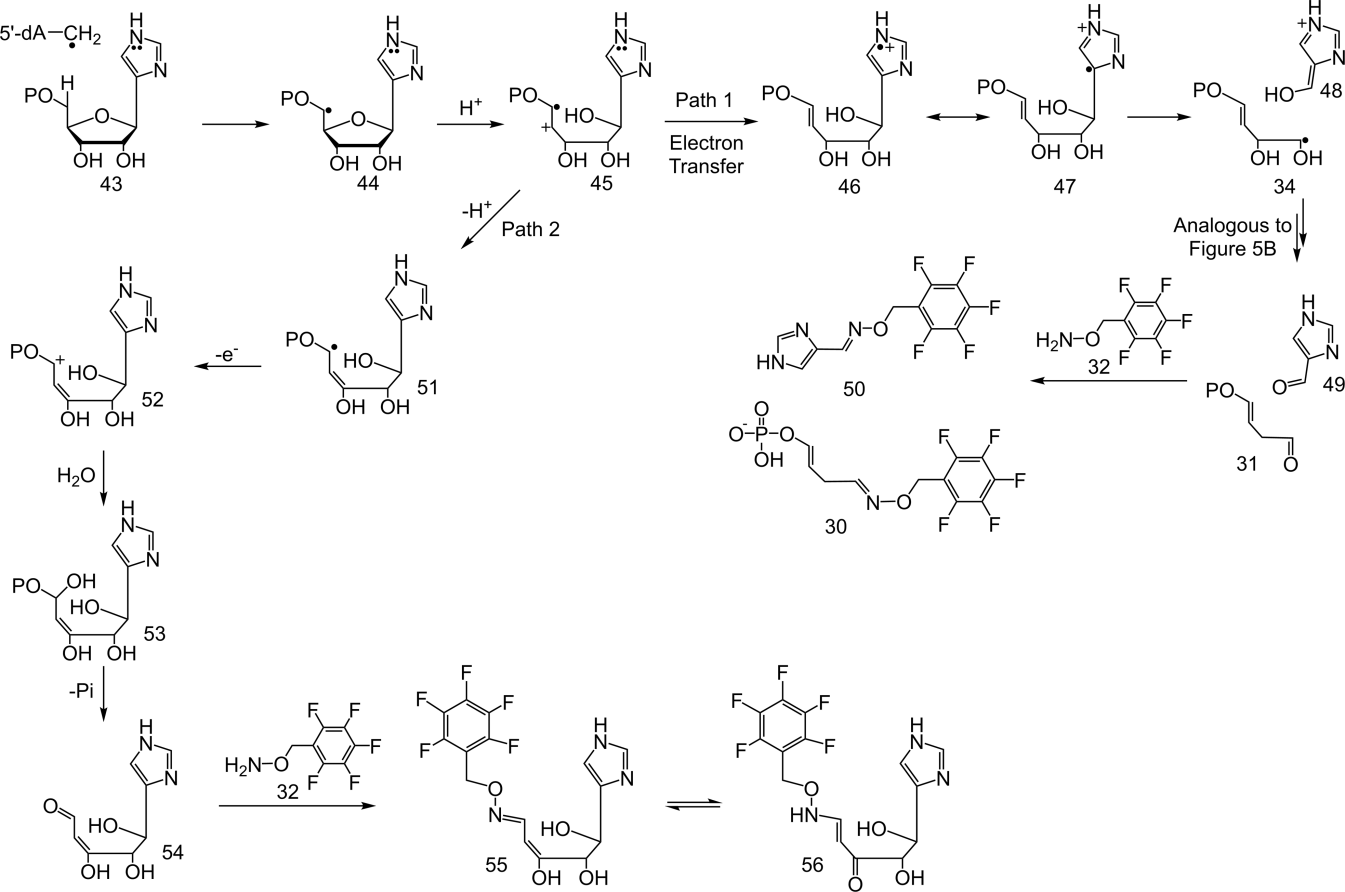
Strategy for trapping formyl imidazole intermediate **39** (Figure 5) as **50** and the product of the first β-scission reaction **14** (Figure 3) as **55**/**56**.

Our mechanistic analysis suggests that the formation of shunt product **31** is accompanied by the formation of formyl imidazole **39** (Figure 5C). Our initial attempts to detect **39** or **16** in the ThiC reaction mixtures were unsuccessful, most likely because *N*-formyl imidazole is readily hydrolyzed, and aminoimidazole **16** reacts rapidly with oxygen.^26^ To surmount these difficulties, we synthesized an AIR analogue **43** in which the labile C–N bond of AIR is replaced by a stable C–C bond (Figures 7, S23 and S24).^42^ In the event, LC-MS analysis of the reaction of **43** with *At*ThiC, under standard reaction conditions, followed by the addition of PFBHA, resulted in the formation of both **30** and **50** (Figures 7, S25 and S26). The structure of **50** was confirmed by coelution with a synthetic standard (Figure S27 and S28). The mechanism for the formation of **50** and **30** in the *At*ThiC reaction with **43** is similar to the native mechanism described in Figure 5A (Figure 7, Path 1). The detection of **50** provides evidence for the formation of formyl imidazole **39** in the native ThiC reaction.

In the reaction mixture of *At*ThiC with **43**, we also detected an additional trapped product by LC-MS analysis with a predicted structure of **55**/**56** (Figures 7, Path 2 and S29). Since aminoimidazole is a better reducing agent than imidazole, we propose that the reduction of enol phosphate radical cation **14** (Figure 5A) is faster than its reduction in **45**, thus allowing time for **45** to undergo deprotonation and oxidation (electron transfer to [Fe_4_S_4_]^+2^) to form **52**. Hydration of **52** followed by phosphate elimination forms **54**. Derivatization of **54** with PFBHA forms **55**, which exists in equilibrium with **56**. The detection of **55**/**56** supports that the cleavage of the C4′-O bond is the first reaction of the AIR radical **13** (Figure 5A).

### Studies on *Cc*ThiC (C474S)

Cys474 is located near AIR in the structure of *Cc*ThiC (Figure S9). The function of this residue cannot be deduced from the structure, most likely because the conversion of the enzyme–substrate complex to the enzyme product complex involves a major rearrangement. However, since active site cysteines generally play an important catalytic role, we constructed the C474S mutant for further study.

HPLC analysis of the *Cc*ThiC (C474S) reaction mixture prior to derivatization with PFBHA demonstrated the consumption of AIR and the formation of 5′-dA. HMP-P was not detected (Figure S30). This suggested the formation of a shunt product in the *Cc*ThiC (C474S) reaction. Carbon monoxide was also not detected, demonstrating that the shunt product is formed prior to the decarbonylation reaction (Figure S31 and **28** to **29**, Figure 3). When the reaction was run using 4′-^2^H-AIR, deuterium transfer from C4′ to the 5′-dA radical occurred, demonstrating that the shunt product is formed after the second hydrogen atom abstraction (Figure S32 and **18** to **19**, Figure 3).

**Figure 8:**
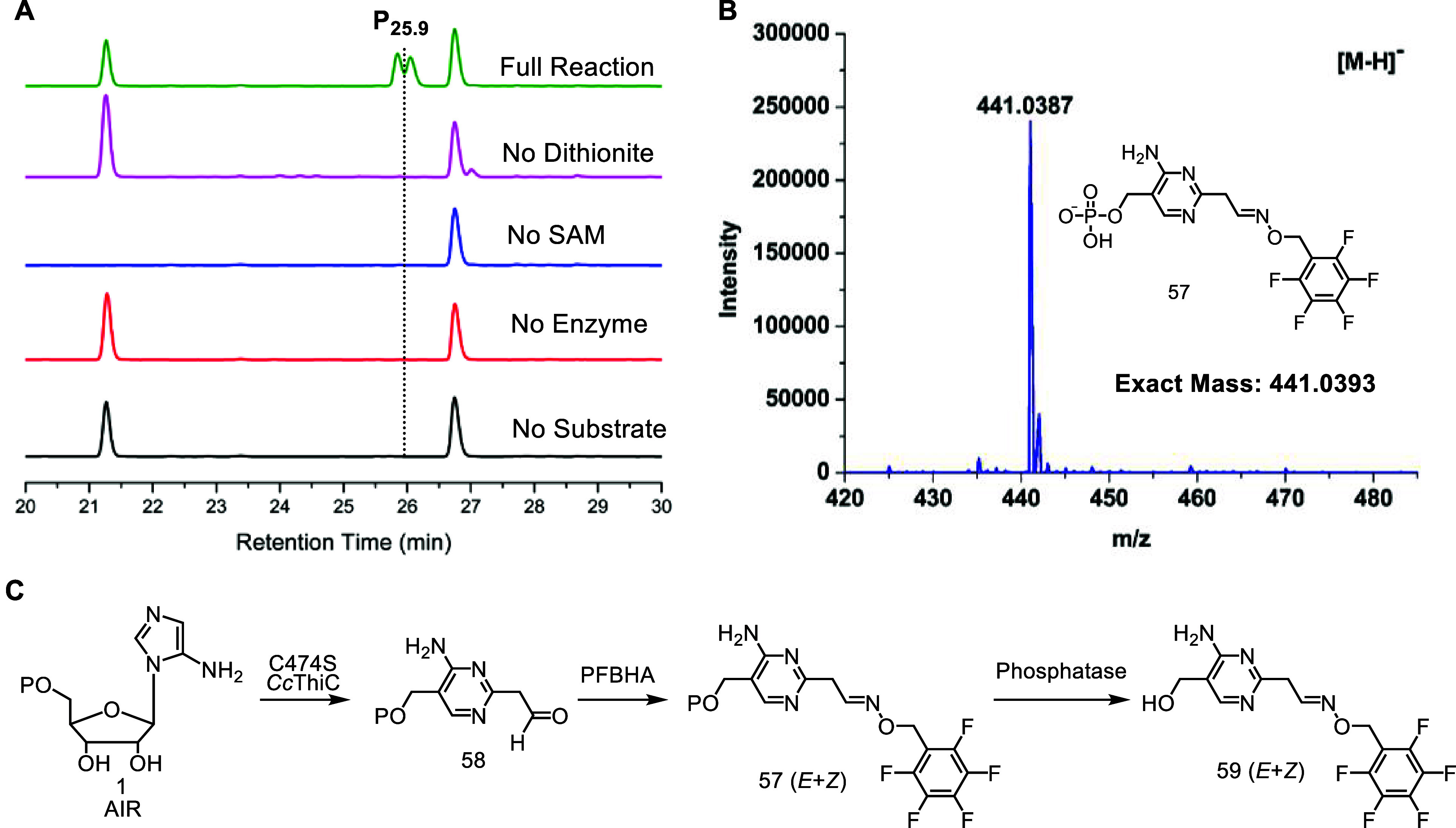
Reactions leading to the formation of **57** (*E* + *Z*). (A) HPLC analysis of the *Cc*ThiC (C474S) catalyzed reaction after the addition of PFBHA and (B) LC-MS of P_25.9_ (**57**). (C) The *Cc*ThiC (C474S) catalyzed reaction and subsequent derivatization.

HPLC analysis of the PFBHA derivatized reaction mixture showed a pair of peaks, characteristic of the *E* and *Z* isomers of PFBHA-oximes, eluting at 25.9 min (P_25.9_). These peaks were absent in all of the indicated control reactions (Figure 8A). LC-MS analysis of P_25.9_ showed a [M-H]^−^ of 441.0387 Da and [M + H]^+^ of 443.0538 Da (Figures 8B, S33–S36). LC-MS analysis using isotopologues of AIR suggested structure **57** for P_25.9_ (Figure S37). The *Cc*ThiC (C474S) reaction was then scaled up, derivatized, and dephosphorylated to generate **59** for NMR analysis (Figures 8C and S38–S40). This analysis confirmed structure **59** (Figures S41–S47). The **57**:5′-dA ratio was 1:6 (Figure S48). We also detected low levels of **57** in the native ThiC reaction, suggesting that **58** is closely related to an on-pathway intermediate (Figure S49).

LC-MS analysis of the *Cc*ThiC (C474S) catalyzed reaction mixture also shows the formation of two minor products **67** and **68** (Figures S50–S53, 10). These products are likely formed by substituting the phosphate in **58** with bisulfite and thiosulfate—both known degradation products of dithionite in water.^27^ This type of substitution has good precedent in thiamin chemistry and enzymology.^43−48^

**Figure 9:**
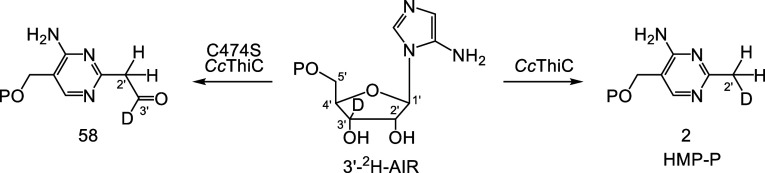
Labeling experiment that demonstrated that the shift of the C3′-H to C2′ occurred after the formation of the radical precursor to **58**.

Previous labeling studies have demonstrated that the methyl group of HMP-P is derived from the C2′ of AIR and that the C3′ carbon is converted to carbon monoxide. The methyl protons are derived from C2′-H, C3′-H, and the buffer (Figure 2A). To determine if the shift of C3′-H to C2′ had occurred in **58**, 3′-^2^H-AIR was treated with *Cc*ThiC (C474S), and the resulting reaction mixture was derivatized with PFBHA and analyzed by LC-MS and MS-MS (Figure S54). This analysis demonstrated that the C3′-H of AIR had not yet migrated to C2′ in **58** (Figure 9).

**Figure 10:**
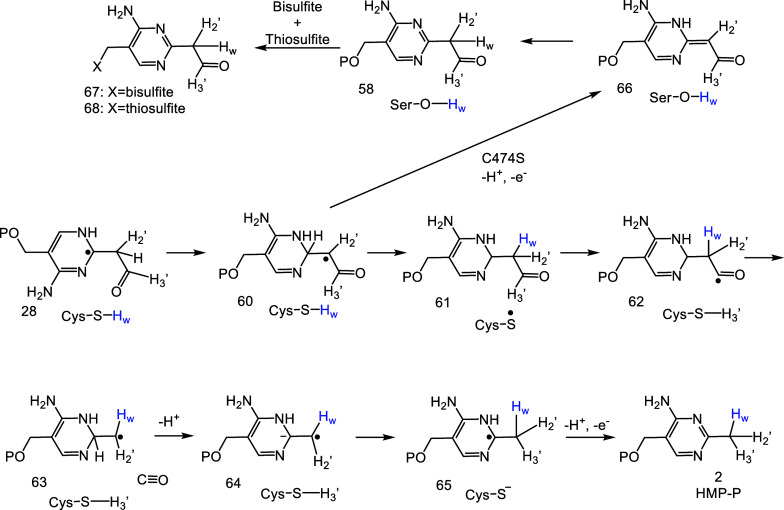
Proposed mechanism for the formation of the thiamin pyridine methyl group and the shunt products **58**, **67**, and **68**.

The structure of trapped intermediate **58** is inconsistent with the mechanism for the formation of the pyrimidine methyl group proposed in Figure 3. The identification of an essential catalytic cysteine at the active site, the trapping of **58** as a shunt product, and the demonstration that the C3′-H of AIR had not yet migrated to C2′ in **58** allows us to propose reasonable chemistry for the formation of the pyrimidine methyl group from intermediate **28** (Figure 3). We propose that **28** undergoes tautomerization to form **60** (Figure 10). Hydrogen atom abstraction by **60** from C474 gives **61**. The resulting cysteine radical abstracts the aldehydic hydrogen from **61** to give **62**, which then undergoes facile decarbonylation to give **63**.^49^ Deprotonation of **63** gives **64**, which is then protonated by Cys474 to give **65**. Electron transfer back to the initially oxidized [Fe_4_S_4_]^+2^ cluster gives HMP-P. In the C474S mutant, the hydrogen atom transfer between pyrimidine radical **60** and Cys474 is blocked (**60** to **61**). Deprotonation of **60** followed by electron transfer to the [Fe_4_S_4_]^+2^ cluster gives **66**, which then undergoes a tautomerization to give the observed trapped product **58**. **58** reacts with bisulfite and thiosulfate to form **67** and **68**, respectively (Figures 10, S51 and S53).

## Discussion

The ThiC mechanistic proposal shown in Figure 3 is supported by extensive labeling studies that identify the origin of each atom in the products.^16,17^ It is also consistent with our observation that 5′-dA returns a hydrogen atom to the C5′ carbon of **17** (**17** to **18**) and that the resulting 5′-dA radical then abstracts a hydrogen atom from C4′ of **18**.^17,18^ In addition, each step in the proposal has a reasonable chemical precedent. While this mechanism is underdetermined, it served as a reasonable starting point for further study. Here, we identify five trapped intermediates that provide new constraints on the mechanistic analysis. The structures of these trapped products and our mechanistic analysis for each are summarized in Table 1.

**Table 1:**
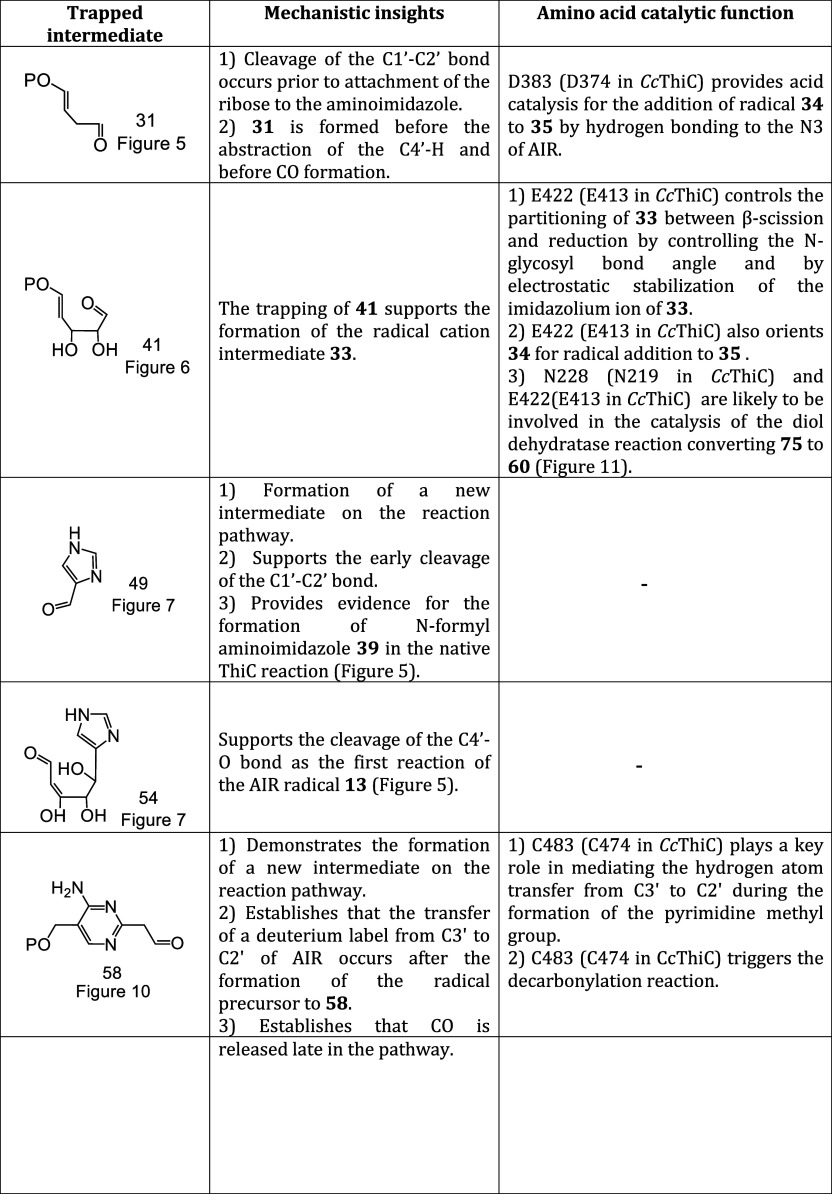
Summary of ThiC Intermediate Trapping Experiments

**Figure 11:**
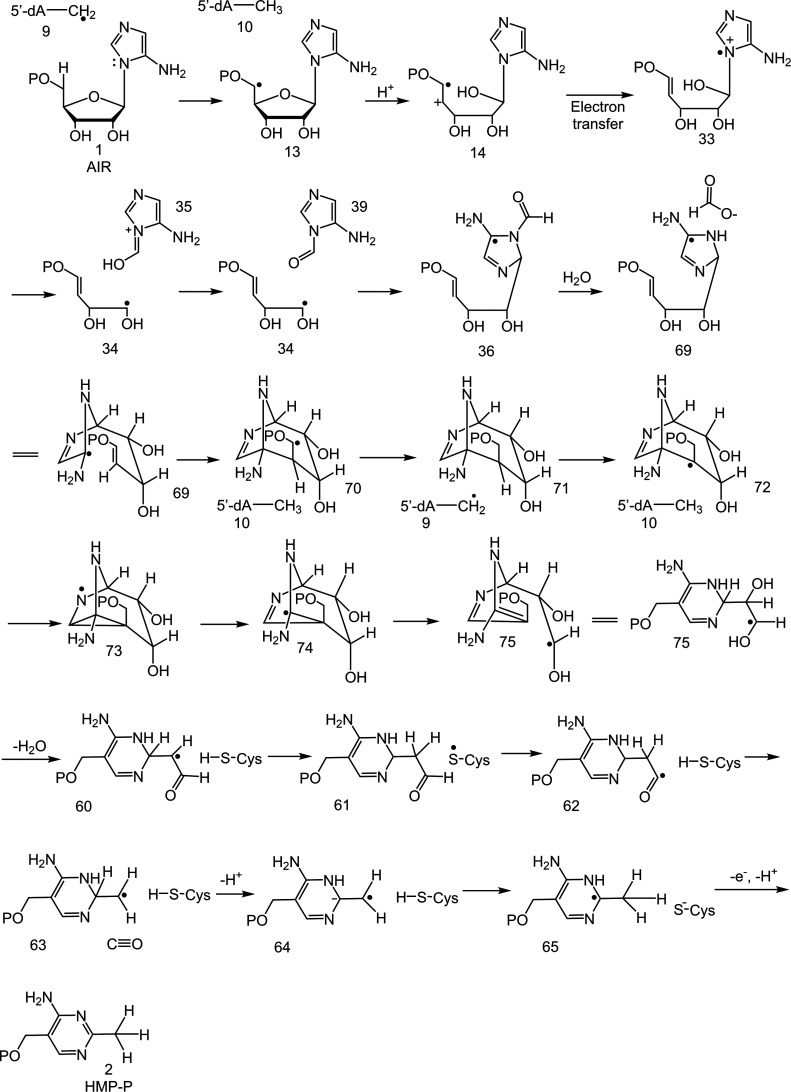
Current mechanistic proposal for the ThiC-catalyzed reaction based on the trapping of five reaction intermediates derived from 14 (**54**), 33 (**41**), 34 (**31**), 39 (**49**), and 60 (**58**).

A mechanism consistent with these new data is shown in Figure 11. In this mechanism, the 5′-dA radical abstracts a hydrogen atom from C5′ of AIR. The resulting radical **13** undergoes β-scission^33−35^ to give **14** (supported by the isolation of **41** and **54**), followed by electron transfer from the electron-rich aminoimidazole to the enol phosphate radical cation to give **33**. This intermediate has been previously detected by EPR in the BzaF-catalyzed reaction, which converts AIR to the dimethylbenzimidazole ligand of vitamin B_12_.^36^ β-scission of the C1′-C2′ bond gives **34** and **35** (supported by the isolation of **31** and **49**). A tautomerization of **35** gives **39**. The addition of radical **34** to **39** gives **36**. The addition of a carbon-centered radical to an imidazole carbon has been observed in diphthamide biosynthesis,^50^ and radical additions to the C=N double bond of pyridine is acid catalyzed.^37,38^ Loss of formate gives **69**. Radical addition to the enol-phosphate of **69** gives radical **70**, which is quenched by hydrogen atom abstraction from 5′-dA to give **71**. Hydrogen atom transfer from C4′ of **71** to the 5′-dA radical gives **72**. This unusual 5′-dA-assisted 1,2 hydrogen atom shift is supported by previously reported labeling studies.^17,18^ Intermediate **72** is well set up for a Beckwith rearrangement^19^ to give **74** via the intermediacy of **73**. A beta-scission reaction of **74** gives **75**. Loss of water from **75** gives **60** (supported by the isolation of **58**). This reaction is well-precedented in the diol dehydratase and the ribonucleotide reductase-catalyzed reactions.^20,21^ Two cysteine-mediated hydrogen abstraction reactions give **62** which then eliminates carbon monoxide in a well-precedented alpha-cleavage of an acyl radical.^49^ This function of Cys474 is supported by studies of the C474S mutant, which results in the trapping of **60**. Deprotonation of **63** gives **64**. Large acidity enhancements of protons beta to radicals have been previously reported.^22,23,51^ Proton transfer from Cys474 gives **65**. Electron transfer to the initially oxidized [Fe_4_S_4_]^+2^ cluster completes the formation of HMP-P.

**Figure 12:**
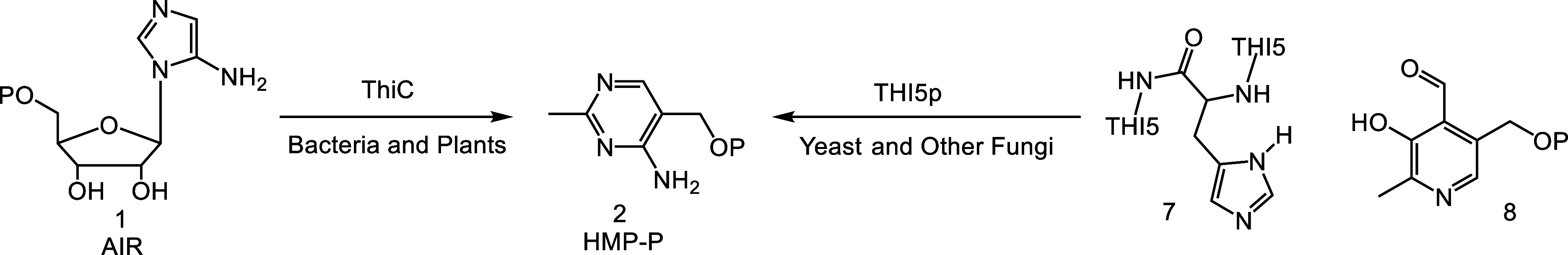
ThiC and the THI5-catalyzed biosynthesis of HMP-P.

**Figure 13:**
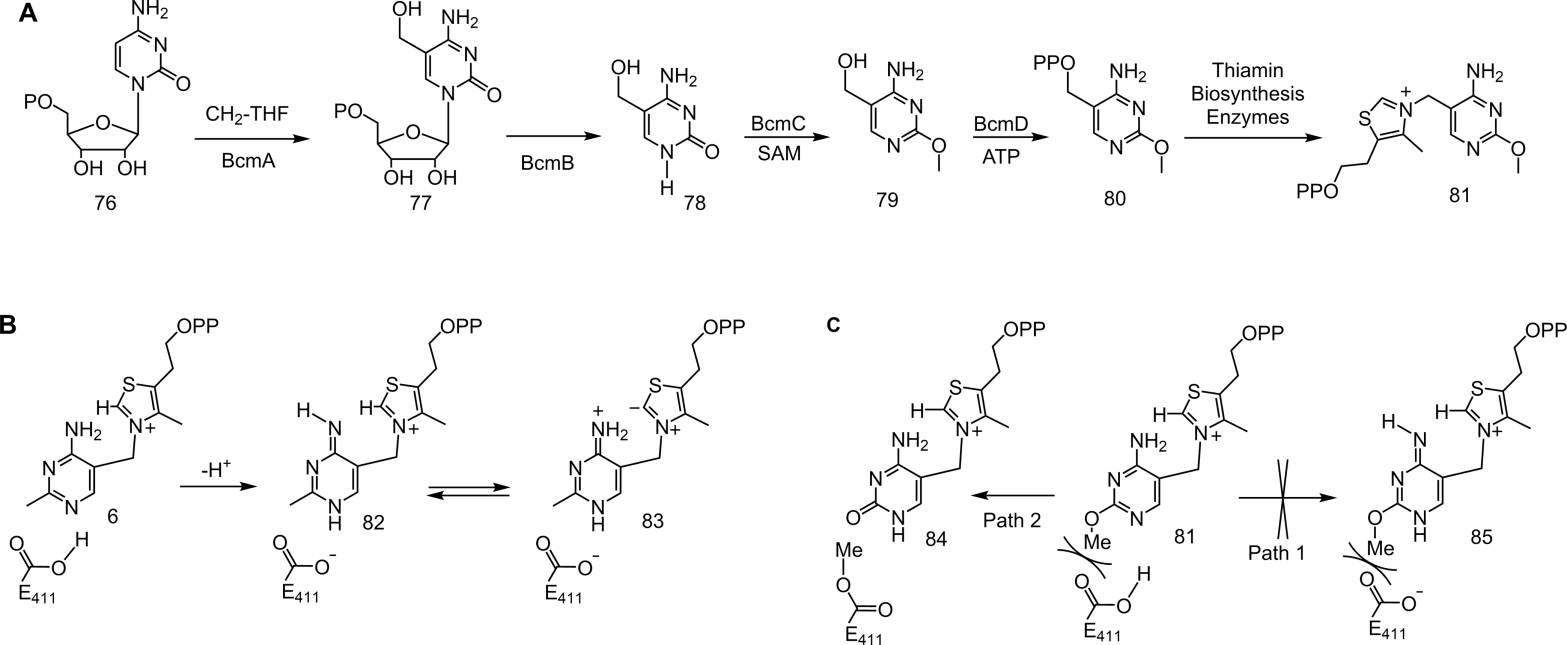
(A) Biosynthesis of bacimethrin **79** and its conversion to methoxythiamin pyrophosphate **81** using late-stage thiamin biosynthetic enzymes. (B) Tautomerization of the thiamin pyrimidine to form the base (**82**) used in the formation of the thiamin ylide (**83**). (C) Mechanism of transketolase inhibition by MeO-TPP (Path 1) and proposed demethylation of MeO-TPP to form the 2′-oxythiamin pyrophosphate **84** antivitamin (Path 2).

Two different routes exist for the biosynthesis of the thiamin pyrimidine (Figure 12). Both pathways are complex and use unprecedented biosynthetic chemistry. In bacteria and plants, the pyrimidine is made by a remarkable radical rearrangement of AIR, while in yeast and other fungi, the pyrimidine is assembled from active site histidine and bound PLP via a formal Diels–Alder reaction of the histidine C=N double bond to oxidatively dearomatized PLP.^12^ While the role of the thiamin pyrimidine in forming the thiamin ylide is well understood (Figure 13B), the biosynthetic logic underpinning the pyrimidine biosynthesis is unknown. In particular, it is unclear why thiamin pyrimidine biosynthesis does not use the robust chemistry of nucleic acid pyrimidine biosynthesis.

To address this, we have studied the biosynthesis and toxicity of bacimethrin **79**, previously identified as a thiamin antivitamin.^52,53^ This compound is an analog of HMP and is biosynthesized from cytidine monophosphate (CMP, **76**), as shown in Figure 13A.^54^ It is activated in vivo by conversion to 2′-methoxythiamin pyrophosphate (MeO-TPP, **81**) using the late-stage thiamin pyrophosphate biosynthetic enzymes.^53^ In vivo studies of *E. coli* mutants in each of the seven TPP-dependent enzymes demonstrated that α-ketoglutarate dehydrogenase, transketolase, and deoxy-d-xylulose-5-phosphate synthase were the major targets for inhibition by MeO-TPP.^53,55^ The structure of transketolase with bound MeO-TPP demonstrated that the methoxy group prevents the protonation of N1′ of the pyrimidine.^56^ This protonation is essential for generating the base **82** needed to form the thiamin ylide (**83**, Figure 13B). Thiamin-dependent enzymes not inhibited by MeO-TPP presumably do not have this clash.^56^ In addition, while MeO-TPP is stable in aqueous buffer, it may not have high biostability, and it may be susceptible to enzyme-catalyzed demethylation to give 2′-oxythiamin pyrophosphate **84**, a known thiamin antivitamin^57^ and methylated enzyme (Figure 13C, Path 2).

This analysis suggests that MeO-TPP **81** might be an ancestral relic of thiamin, in which cofactor activity was achieved by rotating the methoxy group away from the carboxylic acid involved in the pyrimidine tautomerization. If so, replacing the oxygen at C2′ of the pyrimidine with a methyl group may have been a key challenge in the evolution of the current thiamin pyrimidine biosynthesis. The pyrimidine methyl group is important, as suggested by the bacimethrin study above and the observation that desmethyl thiamin pyrophosphate typically shows <10% of the activity of the cofactor.^58,59^ Since there is no precedent for biochemical deoxygenation of uracil or related pyrimidines, the biosynthesis of the thiamin pyrimidine in its current form would have required a major redesign of nucleic acid pyrimidine biosynthesis. Two possibilities are shown in Figure S55.

Radical SAM enzymes are likely to be highly evolvable because radical reactions are generally very fast and do not require the multiple specific enzyme–substrate interactions required for the catalysis of closed-shell reactions. All that a primitive radical SAM enzyme needs is radical-generating chemistry and a simple binding site that protects substrate and intermediates from nonproductive reactions such as radical coupling and hydrogen atom abstraction. It is therefore likely that the cellular metabolome is constantly screened by available radical SAM enzymes for useful new reactions and that the evolution of complex new activities can occur faster than it does for complex closed-shell reactions. Consistent with this analysis, radical SAM enzymes constitute the largest enzyme superfamily (700,000 estimated members).^60^ We propose that this process of radical SAM enzyme evolution has selected a single enzyme-catalyzed radical cascade reaction to convert AIR, an abundant metabolite, to HMP-P, in preference to a major redesign of nucleic acid pyrimidine biosynthesis (Figure S54).

## Supporting Information Available

Synthesis of compounds **43** and **50**, overexpression and purification of ThiC and its variants, enzyme reconstitution, HPLC and LC-MS analysis of trapped intermediates, isotopologue analysis of trapped intermediates, and NMR characterization of **30** and **59** (PDF)
Transparent Peer Review report available (PDF)

## Abbreviations

*At*ThiC: *Arabidopsis thaliana* phosphomethylpyrimidine synthase
*Cc*ThiC: *Caulobacter crescentus* phosphomethylpyrimidine synthase
SAM: *S*-adenosyl methionine
AIR: 5-aminoimidazole ribotide
HMP-P: 4-amino-5-hydroxymethyl-2-methyl pyrimidine phosphate
HPLC: high-performance liquid chromatography
LC-MS: liquid chromatography–mass spectrometry
EIC: extracted ion chromatogram
TPP: thiamin pyrophosphate
PLP: pyridoxal 5′-phosphate
