# Supplementary material for: Phosphomethylpyrimidine Synthase (ThiC): Trapping of Five Intermediates Provides Mechanistic Insights on a Complex Radical Cascade Reaction in Thiamin Biosynthesis
Source: ACS Cent Sci. 2024 May 13;10(5):988–1000. doi: 10.1021/acscentsci.4c00125 (PMC11117688; doi:10.1021/acscentsci.4c00125)
Supplement: Supplementary file 1 — oc4c00125_si_001.pdf [file oc4c00125_si_001.pdf]

Supporting information

Phosphomethylpyrimidine Synthase (ThiC): Trapping of Five Intermediates Provides Mechanistic Insights on a Complex Radical Cascade Reaction in Thiamin Biosynthesis

Vishav Sharma, Dmytro Fedoseyenko, Sumedh Joshi, Sameh Abdelwahed, Tadhg P. Begley*

Department of Chemistry, Texas A&M University, College Station, Texas 77842, United States

* Email: begley@tamu.edu

Table of Contents

General materials and methods S7

HPLC parameters S7

HPLC conditions S7

HPLC method: (Flow rate 1 mL/min) S8

LC-MS parameters S8

LC conditions S8

LC method: (Flow rate 0.4 mL/min) S8

MS parameters S8

NMR analysis S8

Overexpression and purification of *Arabidopsis thaliana* ThiC, *Caulobacter crescentus* ThiC, and their variants S9

Overexpression and purification of AIRs Kinase S9

Synthesis of 5-aminoimidazole riboside (AIRs, 86) and its isotopologues S10

Enzymatic phosphorylation of AIRs (86) and its isotopologues S10

Figure S1. Enzymatic phosphorylation of AIRs 86. (A) Scheme for enzymatic phosphorylation. (B) HPLC chromatogram for phosphorylation reaction showing the formation of AIR in full reaction (black trace). S10

Enzymatic assay of *Arabidopsis thaliana* ThiC, *Caulobacter crescentus* ThiC, and their variants (No PFBHA derivatization) S10

Figure S2. Phosphatase treatment of 1 and 2 leading to the formation of 86 and 87, respectively S11

Enzymatic assay of *Arabidopsis thaliana* ThiC, *Caulobacter crescentus* ThiC, and their variants (PFBHA derivatization) S11

Figure S3. Extracted Ion Chromatogram (EIC) for the new product P_{17} (30) formed in the *At*ThiC-catalyzed reaction of AIR after PFBHA treatment S12

Figure S4. Characterization of 30 formed in the *At*ThiC-catalyzed reaction of AIR after PFBHA treatment. (A) MS analysis of 30 (positive ion mode). (B) MS-MS analysis of 30 (positive ion mode). S12

Enzymatic assay of *At*ThiC (D383A) with AIR for NMR characterization of 30 .... S13

Figure S5. Identification of 30 in the *At*ThiC (D383A) catalyzed reaction of AIR after PFBHA treatment. (A) HPLC chromatogram showing the formation of 30. The splitting of the peak is due to the presence of *E* and Z isomers of oxime 30. This peak was collected over multiple runs for NMR characterization. (B) Proposed structures for *E* & *Z* isomers of 30. 31 is proposed to be the product of the *At*ThiC (D383A) catalyzed reaction, which reacts with PFBHA to form the *E* and Z isomers of oxime 30. .... S13

Figure S6. ^{31}P NMR spectra of 30. (A) Mixture of potassium phosphate (Kpi) and HPLC purified sample of 30. (B) Potassium phosphate. (C) HPLC purified sample of 30 .... S14

Figure S7. ^{1}H NMR spectra of 30 (^{31}P Coupled)..S14

Figure S8. MS analysis of 30 formed upon using AIR (1) isotopologues and labeled solvent in the *At*ThiC-catalyzed reaction. (A) 1',2',3',4',5'-$^{13}C_5$-1. (B) 1'-^{13}C-1. (C) 5'-$^{2}H_2$-1. (D) 4'-^{2}H-1. (E) 3'-^{2}H-1. (F) 2'-^{2}H-1. (G) 50% $^{2}H_2O$. (H) 50% H_2O^{18}. The 362.01 peak, observed consistently across all spectra, indicates the presence of impurity from an unidentified compound, which is also present in the control samples.......S16

Figure S9. (A) Interactions of AIR with N228, D383, E422, and C483 in the active site of *At*ThiC (PDB:4S28)[8]. (B) Interactions of imidazole ribonucleotide (IRN, 5-desamino AIR) with N217, D374, E413, and C474 in the active site of *Cc*ThiC (PDB:3EPN)[2]. The residue responsible for acid-catalyzed ring opening (13 to 14, Figures 3, 5, and 11) has not been identified. S17

Figure S10. HPLC chromatogram (@240 nm, no PFBHA derivatization) for the reaction of *At*ThiC and its mutants with AIR. (A) No enzyme control. (B) wt *At*ThiC. (C) *At*ThiC (D383N). (D) *At*ThiC (D383A). wt, D383N, and D383A *At*ThiC all consume AIR (detected as 86) and form 5'-deoxyadenosine 10. The formation of HMP-P (detected as 87) is significantly lower in *At*ThiC (D383N), with no formation in *At*ThiC (D383A). The quantitation of these chromatograms is shown in Figure S10. ....S17

Figure S11. Quantitation of HPLC chromatograms shown in Figure S9..S18

Figure S12. LC-MS analysis of 30 formed in the *At*ThiC (D383A) catalyzed reaction of AIR after PFBHA treatment. (A) EIC of 30 (P_{17}). (B) MS analysis of 30. (C) Overlaid EIC of 30 formed in the wt *At*ThiC and *At*ThiC (D383A) catalyzed reactions. The formation of 30 in the *At*ThiC (D383A) catalyzed reaction is significantly higher than the wt *At*ThiC-catalyzed reaction..S18

Quantitation of shunt product (30) in the *At*ThiC (D383A) catalyzed reaction......................................S19

Figure S13. Quantitation of shunt product 30 in the *At*ThiC (D383A) catalyzed reaction. (A) HPLC traces for different concentrations of the formaldehyde PFBHA oxime 86. (B) Calibration curve for 86. ....S19

Procedure for DEPT-90 NMR of the *At*ThiC (D383A) catalyzed reaction with 1'-^{13}C-AIR S19

Figure S14. DEPT-90-NMR analysis of the *At*ThiC (D383A) catalyzed reaction with 1'-^{13}C-AIR showing the formation of ^{13}C formate (171 ppm). S19

Figure S15. HPLC chromatogram (@240 nm, no PFBHA derivatization) of wt *Cc*ThiC and *Cc*ThiC (E413Q) catalyzed reactions of AIR. (A) No enzyme control. (B) wt *Cc*ThiC. (C) *Cc*ThiC (E413Q). *Cc*ThiC (E413Q) consumes AIR (detected as 86) but does not form HMP-P (detected as 87). S20

Figure S16. Characterization of 40 in the *Cc*ThiC (E413Q) catalyzed reaction of AIR after PFBHA treatment. (A) HPLC chromatogram showing the formation of P_{24}(40). (B) EIC (LC-MS) of 40. The difference in retention time on LC-MS is because of different C-18 columns and flow rates used in LC-MS and HPLC analysis. The *E* and *Z* isomers are inseparable by HPLC but separable by LC-MS. S20

Figure S17. MS-MS of 40 in positive ion mode. S21

Figure S18. MS analysis of 40 formed upon using AIR (1) isotopologues and labeled solvent in the *Cc*ThiC (E413Q) catalyzed reaction. (A) 1',2',3',4',5'-$^{13}C_5$-1. (B) 1'-^{13}C-1. (C) 5'-$^{2}H_2$-1. (D) 4'-^{2}H-1. (E) 3'-^{2}H-1. (F) 2'-^{2}H-1. (G) 50% $^{2}H_2O$. (H) 50% H_2O^{18}. (I) 5-^{15}N-1. (J) Summary of labeling studies. S24

Figure S19. Characterization of 30 in the *Cc*ThiC (E413Q) catalyzed reaction of AIR after PFBHA treatment. (A) EIC of 30. (B) Overlaid EIC of 40 and 30 to compare the relative ratio of the two shunt products....S24

Figure S20. HPLC chromatogram of the *At*ThiC (E422Q) catalyzed reaction of AIR after PFBHA treatment showing formation of P_{24} (40)....S25

Figure S21. EIC of 40 in the wt *Cc*ThiC-catalyzed reaction of AIR after PFBHA treatment. S25

Figure S22. Studies on *At*ThiC (N228D) (A) HPLC chromatogram (@240 nm, no PFBHA derivatization) of the *At*ThiC (N228D) catalyzed reaction of AIR showing consumption of AIR (detected as 86) but no HMP-P (detected as 87) formation. (B) HPLC chromatogram (@ 225nm) of *At*ThiC (N228D) catalyzed reaction of AIR after PFBHA treatment showing formation of 30 in full reaction only. (C) MS analysis of 30 formed in the *At*ThiC (N228D) catalyzed reaction of AIR after PFBHA treatment. S26

Synthesis of substrate analog 43 ....S26

Figure S23. Characterization of 89. (A) ^{1}H-NMR of 89. (B) ESI-MS of 89 in positive ion mode. S27

Figure S24. Enzymatic phosphorylation of 89. (A) Scheme for the enzymatic phosphorylation. (B) HPLC chromatogram for the phosphorylation reaction....S28

Figure S25. LC-MS analysis of the *At*ThiC catalyzed reaction of 43 after PFBHA treatment. (A) EIC of 30. (B) MS analysis of 30 (negative ion mode). S28

Figure S26. LC-MS analysis of the *At*ThiC catalyzed reaction of 43 after PFBHA treatment. (A) EIC of 50. (B) MS analysis of 50 (negative ion mode). S29

Synthesis of authentic sample of 50....S29

Figure S27. Synthesis of 50. (A) Scheme for the synthesis of 50. (B) MS analysis of 50 (negative ion mode). (C) ^{1}H-NMR of 50. ....S30

Figure S28. EIC showing the coinjection of the *At*ThiC-catalyzed reaction of 43 with the synthetic standard 50. ....S30

Figure S29. Characterization of 55/56 formed in the *At*ThiC catalyzed reaction of 43 after PFBHA treatment. (A) EIC of 55/56. (B) MS analysis of 55/56. (C) MS-MS analysis of 56. (D) MS analysis of 55/56 after performing the reaction in 50% H_2O^{18} buffer showed an O^{18} incorporation in 55/56. (E) MS analysis of 55/56 after performing the reaction in 50% D_2O buffer showed no solvent-exchangeable protons in 55/56. ....S32

Figure S30. HPLC chromatogram (@240 nm, no PFBHA derivatization) of the *Cc*ThiC (C474S) catalyzed reaction of AIR. AIR (detected as 86) is consumed in the *Cc*ThiC (C474S), but HMP-P (detected as 87) is not formed....S33

Figure S31. Hemoglobin assay for carbon monoxide detection. The red trace shows the wt *Cc*ThiC-catalyzed reaction of AIR in the presence of hemoglobin (50 μM), producing carboxyhemoglobin (430 nm). The black trace shows the *Cc*ThiC (C474S) catalyzed reaction of AIR in the presence of hemoglobin with only deoxyhemoglobin present (419 nm) and no carboxyhemoglobin production. A previously reported procedure was used for this assay.[6] S33

Figure S32. LC-MS analysis of the *Cc*ThiC (C474S) catalyzed reaction with 4'-^{2}H-AIR showing deuterium incorporation in 5'-dA 10 ([M-H]$^{-}$:251.1016). The [M-H]$^{-}$ of 250.09 observed is unlabeled 5'-dA formed due to unproductive hydrogen atom abstraction by 5'-dA radical. S34

Figure S33. LC-MS of 57 formed in the *Cc*ThiC (C474S) reaction (negative ion mode). (A) EIC of 57. (B) [M-H]$^{-}$ for 57. .... S34

Figure S34. LC-MS of 57 formed in the *Cc*ThiC (C474S) reaction (positive ion mode). (A) EIC of 57. (B) [M+H]$^{+}$ for 57. .... S35

Figure S35. MS-MS analysis of 57 in negative ion mode. S35

Figure S36. MS-MS analysis of 57 in positive ion mode. S36

Figure S37. MS analysis of 57 formed upon using AIR (1) isotopologues and labeled solvent in the *Cc*ThiC (C474S) catalyzed reaction. (A) 1',2',3',4',5'-$^{13}C_5$-1. (B) 1'-^{13}C-1. (C) 5-^{15}N-1. (D) 5'-$^{2}H_2$-1. (E) 4'-^{2}H-1. (F) 3'-^{2}H-1. (G) 2'-^{2}H-1. The 2'-^{2}H is retained at the C2' carbon in 58. However, the pKa of 2'-^{2}H decreases because of the α carbonyl group, and it exchanges with solvent. (H) 5'-$^{2}H_2$-1 & $^{2}H_4$-SAM. (I) 50% $^{2}H_2O$. (J) 50% H_2O^{18}. (K) Summary of LC-MS analysis of label transfer from isotopologues of AIR to 57. (L) The *Cc*ThiC (C474S) catalyzed reaction with the fate of all atoms shown in color. S40

Figure S38. HPLC analysis of the *Cc*ThiC (C474S) catalyzed the reaction of AIR after CIP treatment and the addition of PFBHA, showing a new product $P_{28.6}$ (59) in the full reaction only. 59 is the dephosphorylated form of 57. .... S40

Figure S39. HPLC analysis of the *Cc*ThiC (C474S) catalyzed reaction of AIR after the addition of PFBHA shows the formation of $P_{25.9}$ (57) (Red Trace), and HPLC analysis of the *Cc*ThiC (C474S) catalyzed reaction of AIR after CIP treatment and the addition of PFBHA shows the formation of $P_{28.6}$ (59) (Black Trace). The comparison shows that 57 is converted to 59 upon CIP treatment. S41

Figure S40. LC-MS of 59 in the *Cc*ThiC (C474S) catalyzed reaction (negative ion mode). (A) EIC of 59. (B) [M-H]$^{-}$ for 59. .... S41

Figure S41. Structures of different isomers of 59 (A) *Z* isomer of 59 with hydrogens annotated from a-f and carbons annotated from 1-10. (B) *E* isomer of 59 with hydrogens annotated from a' -f' and carbon annotated from 1' -10'. S42

Figure S42. ^{1}H-NMR of 59. .... S42

Figure S43. ^{13}C-NMR of 59. .... S43

Figure S44. DEPT-135 NMR of 59. .... S43

Figure S46. ^{1}H-^{13}C HSQC NMR of 59. .... S44

Figure S47. ^{1}H-^{13}C HMBC NMR of 59. .... S44

Quantitation of shunt product 57 in the *Cc*ThiC C474S reaction S45

Figure S48. Quantitation of shunt product 57 in the *Cc*ThiC (C474S) catalyzed reaction of AIR. (A) HPLC traces for different concentrations of formaldehyde PFBHA oxime 88. (B) Calibration curve for 88. (C) HPLC traces for different concentrations of HMP 87. (B) Calibration curve for 87. .... S46

Figure S49. LC-MS of 57 formed in the wt *Cc*ThiC-catalyzed reaction of AIR (negative ion mode) (A) EIC of 57. (B) $[M-H]^-$ for 57. ....S46

Figure S50. LC-MS of 67 in the *Cc*ThiC (C474S) catalyzed reaction (negative ion mode). (A) EIC of 67. (B) $[M-H]^-$ for 67 ....S47

Figure S51. Proposed mechanism for the formation of 67 in the *Cc*ThiC (C474S) catalyzed reaction. ..S47

Figure S52. LC-MS of 68 formed in the *Cc*ThiC (C474S) catalyzed (negative ion mode). (A) EIC of 68. (B) $[M-H]^-$ for 68 ....S48

Figure S53. Proposed mechanism for the formation of 68 in the *Cc*ThiC (C474S) catalyzed reaction. ..S48

Figure S54. (A) Positive mode MS-MS analysis of 57 formed in the *Cc*ThiC (C474S) catalyzed reaction of AIR. (B) Positive mode MS-MS analysis of 57 formed in the *Cc*ThiC C474S reaction with 3'-^{2}H-AIR. S49

Figure S55 . Proposed alternative routes to HMP. A) Route based on bacimethrin biosynthesis. B) Route based on the canonical pyrimidine biosynthesis. Extensive genome sequence analysis of thiamin biosynthetic gene clusters suggests that the ThiC and THI5 catalyzed reactions are the only routes to the thiamin pyrimidine. S50

References S51

General materials and methods

All chemicals were purchased from Sigma Aldrich (St. Louis, MO) unless otherwise stated and used without further purification. Quick CIP was purchased from NEB (M0525S). LB growth medium was obtained from Difco. Kanamycin, ampicillin, chloramphenicol antibiotics, and isopropyl-β-D-thiogalactopyranoside (IPTG) were purchased from LabScientific Inc. Benzonase and Lysozyme were purchased from Sigma Aldrich. His-trap columns (5 ml) were obtained from GE healthcare. Econo-Pack 10DG and Bio-spin 6 desalting columns were purchased from Bio-Rad Laboratories. Large cultures were grown and overexpressed in 2.5 L baffled ultra-yield flasks from Thomson Instrument Company. NMR tubes (3 mm and 5 mm diameter) were obtained from Wilmad-Labglass. D_2O and MeOD, were purchased from Cambridge Isotope Laboratories Inc. Centrifugal filters were obtained from Pall Life Sciences. HPLC grade solvents were obtained from Fisher Scientific.

HPLC parameters

An Agilent 1260 HPLC equipped with a quaternary pump was used. The system included a diode array UV-Vis detector and eluted compounds were detected by absorbance at 254, 215, 225, 240, 280, 309, 288, and 475 nm. The parameters for the fluorescence detector were: excitation at 385 nm and emission at 484 nm. The HPLC analysis was performed on a ZORBAX Eclipse XDB-C18 column (15 cm x 4.6 mm, 5 µm particles, Agilent Technologies). Typical injection volumes were in the range of 10-50 µL. Data were processed using ChemStation ver. B.04.01 SP1 (Agilent technologies).

HPLC conditions

For analysis:

A- Water
B- 100 mM Potassium phosphate buffer, pH 6.6
C- Methanol

For purifying compounds via HPLC:

A- Water
B- 5 mM Ammonium acetate, pH 6.6
C- Methanol

HPLC method: (Flow rate 1 mL/min)

Method 1 (for ThiC reactions with no PFBHA derivatization):

0 min – 100% B, 5 min – 100% B, 12 min – 48% A 40% B 12% C, 14 min – 50% A 30% B 20% C, 18 min - 30% A 10% B 60% C, 20 min - 100% B, 25 min - 100% B.

or

Method 2 (for ThiC reactions with PFBHA derivatization):

0 min – 100% B, 5 min – 100% B, 12 min – 48% A 40% B 12% C, 14 min – 7% A 70% B 23% C, 25 min - 25% A 75% C, 28 min - 25% A 75% C, 32 min - 100% B, 37 min - 100% B.

LC-MS parameters

LC-ESI-TOF-MS was performed using an Agilent 1260 HPLC system equipped with a binary pump and a 1200 series diode array detector followed by a MicroToF-Q II mass spectrometer (Bruker Daltonics) using an ESI source either in negative or positive mode. The analysis was performed on an LC-18-T column (15 cm x 3 mm, 3 μm particles, Supelco). Typical injection volumes were in the range of 20-80 μL. The data was processed using DataAnalysis 4.0 SP1 (Bruker Daltonics).

LC conditions

A- 5 mM Ammonium acetate buffer, pH 6.6
B- 75% Methanol and 25% Water

LC method: (Flow rate 0.4 mL/min)

0 min – 100% A, 7 min – 100% A, 10 min – 80% A 20%B, 27 min – 100%B, 29 min – 100% B, 30 min – 100% A, 40 min – 100% A.

MS parameters

Capillary, -4500 V; capillary offset, -500 V; nebulizer gas, 3.0 bar; dry gas, 10 L/min; dry gas temperature, 200 ºC; funnel 1 RF, 250.0 Vpp; funnel 2 RF, 300.0 Vpp; ISCID, 0.0 eV; hexapole RF, 200 Vpp; quadrupole ion energy, 3.0 eV; collision cell, collision energy, 8.0 eV; collision RF, 150.0 Vpp, transfer time, 80.0 μs; prepulse storage, 5.0 μs.

NMR analysis

NMR spectra of all the synthetic samples were recorded on a Bruker Avance III 400 MHz instrument. NMR characterization of samples collected by HPLC was performed on Bruker

Avance III 500 MHz instrument with an H-C-N cryoprobe in 3 mm Wilmad labglass (328-PP-7) high-precision NMR tubes.

Overexpression and purification of *Arabidopsis thaliana* ThiC, *Caulobacter crescentus* ThiC, and their variants

Arabidopsis thaliana ThiC used is an N-terminal truncated version lacking the first 71 amino acids.[1] The same template of *Arabidopsis thaliana* ThiC was used to synthesize the mutants. The ThiC gene was cloned into the pTHT vector (a derivative of pET28b vector with a TEV protease cleavage site after the N-terminal His-tag).[2] ThiC was co-expressed with a plasmid encoding the *suf* operon in *E. coli* BL21(DE3) for in vivo assembly of the $[Fe_4S_4]$ cluster.[3] A starter culture was grown overnight in LB media containing kanamycin (40µg/ml) and chloramphenicol (34µg/ml). 90 ml of this culture was added to 9 L of LB media (6 x 1.5 L flasks) with antibiotics and grown at 37 °C with shaking (220 rpm) till OD_{600} ~ 0.6. The flasks were then incubated at 4 °C for ~1 h without shaking. Each flask (1.5 L) was supplemented with 120 mg ferrous ammonium sulfate and 120 mg L-cysteine. Cultures were then induced with 500 µM IPTG followed by incubation at 15 °C for ~16-18 h with shaking (110 rpm). The cells were harvested by centrifugation and stored in liquid nitrogen until further use. Typical yields were 20-25 g of cell pellets from 9 L cell culture.

All steps for ThiC purification were carried out in an anaerobic chamber (COY laboratories). Cell pellets (10-12 g) were thawed and resuspended in 50-60 mL of lysis buffer (100 mM Tris-HCl, pH 7.5) at room temperature in the presence of lysozyme (10-12 mg) and benzonase (1000 units). The suspension was stirred for ~30 min on an ice bath and further sonicated to lyse the cells. Sonication was done for 90–120 s with 90% amplitude (repeat this step 4–5 times with 6–8 min of stirring between each cycle). Cell debris was removed by centrifugation, and the lysate was loaded onto a His-trap column preequilibrated with lysis buffer. The column was washed with 10 column volumes of wash buffer (100 mM Tris-HCl, 30 mM imidazole, 300 mM NaCl, pH 7.5). The protein was then eluted from the his-trap column with elution buffer (100 mM Tris-HCl, 250 mM imidazole, 300 mM NaCl, pH 7.5). The dark-colored fractions were pooled and buffer-exchanged to 100 mM Tris-HCl, 30% glycerol, pH 7.5, using an Econo-Pac 10DG desalting column. Typical yields were ~ 6-8 mg per liter of cell culture. The purified enzyme was stored submerged in liquid nitrogen until further use. Protein concentration was measured by the absorbance at 280 nm (A_{280}) with an extinction coefficient calculated using the ProtParam tool of the ExPASy proteomics server.

Overexpression and purification of AIRs Kinase

AIRs kinase from *Salmonella enterica* was overexpressed in *E. coli* B834(DE3) and purified using Ni-NTA chromatography per the published protocol.[4]

Synthesis of 5-aminoimidazole riboside (AIRs, 86) and its isotopologues

AIRs **86** and its isotopologues were synthesized according to the published procedures.[5-7]

Enzymatic phosphorylation of AIRs (86) and its isotopologues

All the stocks were prepared in 100 mM Tris-HCl buffer (pH 7.5). A typical reaction mixture consisted of 100 mM Tris-HCl, 2.5 mM AIRs, 4 mM adenosine triphosphate (ATP), 50 mM $MgSO_4.7H_2O$, and 400 μM AIRs kinase. The reaction mixture was incubated at room temperature for 1 hour and analyzed by HPLC (Figure S1). AIR **1** was purified from large-scale AIRs Kinase reactions (8-10 mL reactions) using HPLC and lyophilized to dryness. Lyophilized AIR was then transferred into the glove box and resuspended in degassed water to a final AIR concentration of 5-10 mM. This AIR stock was used for ThiC reactions. To measure the concentration of purified AIR, it was treated with phosphatase to obtain AIRs. A calibration curve of a synthetic standard of AIRs was then used to measure the concentration of phosphatase-treated AIR. The same procedure was used for the phosphorylation of isotopologues of AIRs.

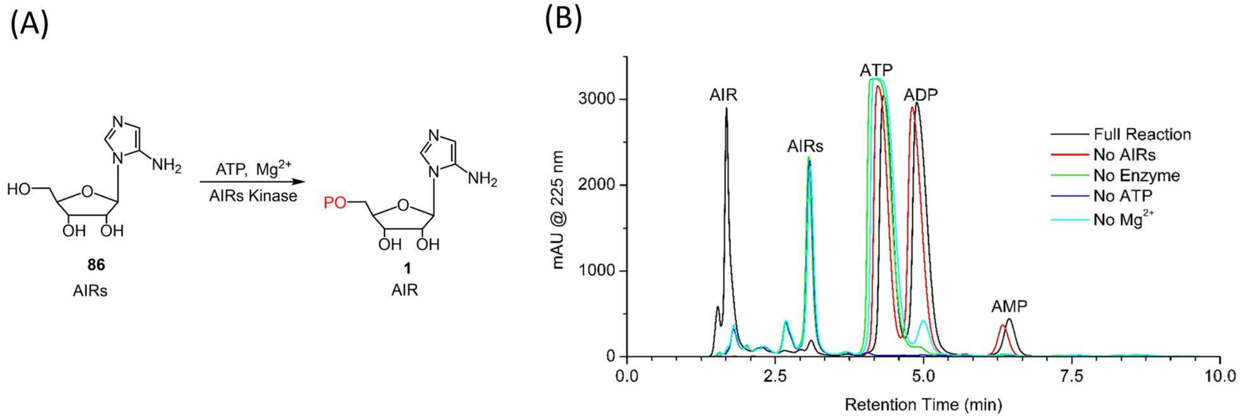

Figure S1. Enzymatic phosphorylation of AIRs **86**. (A) Scheme for enzymatic phosphorylation. (B) HPLC chromatogram for phosphorylation reaction showing the formation of AIR in full reaction (black trace).

Enzymatic assay of *Arabidopsis thaliana* ThiC, *Caulobacter crescentus* ThiC, and their variants (No PFBHA derivatization)

A typical enzymatic reaction (100 μL) was performed in 100 mM Tris-HCl buffer (pH 7.5) containing 300 μM ThiC, 500 μM AIR, 1 mM SAM, and 6 mM titanium (III) citrate. For studies on *Cc*ThiC (C474S), titanium (III) citrate was replaced with 2 mM sodium dithionite. The reaction was incubated anaerobically at room temperature for 6 hours. 1 μL Calf intestinal phosphatase

(CIP) was added to 100 μL of ThiC enzymatic reaction. The mixture was incubated at room temperature for 60 minutes. The reaction mixture was heat quenched at 100 °C for one minute and then centrifuged and filtered through a 10 kDa cut-off filter before being analyzed by HPLC or LC-MS. Treatment of ThiC reaction with CIP dephosphorylates the substrate AIR to **86** and product HMP-P to **87**, leading to a longer retention time on the C-18 column.

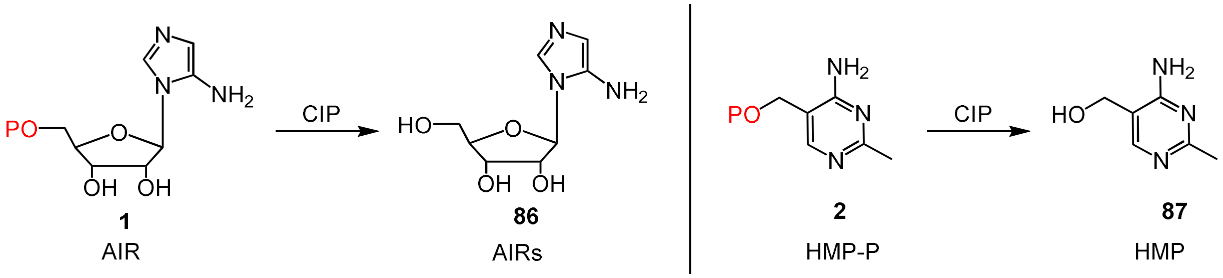

Figure S2. Phosphatase treatment of **1** and **2** leading to the formation of **86** and **87**, respectively.

Enzymatic assay of *Arabidopsis thaliana* ThiC, *Caulobacter crescentus* ThiC, and their variants (PFBHA derivatization)

A typical enzymatic reaction (100 μL) was performed in 100 mM Tris-HCl buffer (pH 7.5) containing 300 μM ThiC, 500 μM AIR, 1 mM SAM, and 6 mM titanium (III) citrate. For studies on *Cc*ThiC (C474S), titanium (III) citrate was replaced with 2 mM sodium dithionite. The reaction was incubated anaerobically at room temperature for 6 hours. 50 mM PFBHA (10 μL; 5 mM final concentration) was added to 90 μL of ThiC enzymatic reaction. The mixture was incubated at 60 °C for 30 minutes. The reaction mixture was heat quenched at 100 °C for one minute and then centrifuged and filtered through a 10 kDa cut-off filter before being analyzed by HPLC or LC-MS.

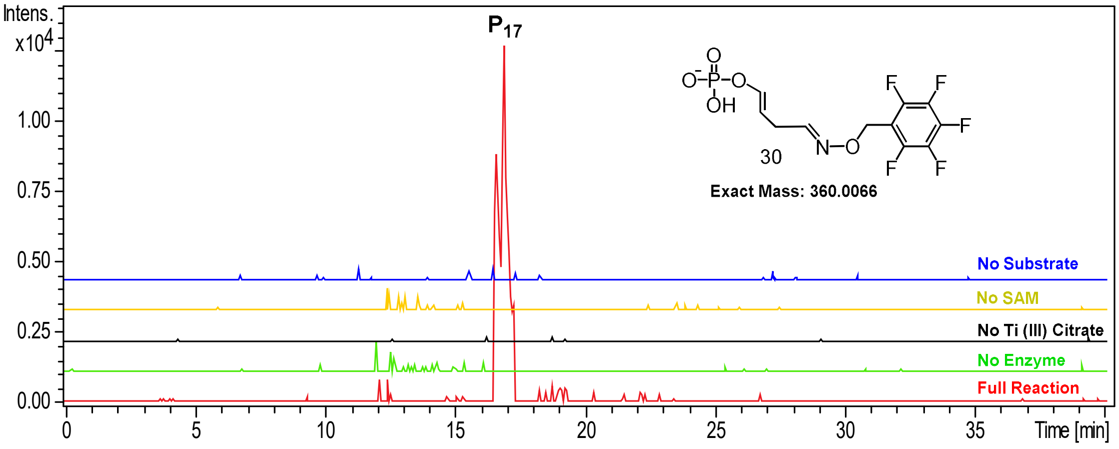

Figure S3. Extracted Ion Chromatogram (EIC) for the new product P_{17} (**30**) formed in the *At*ThiC-catalyzed reaction of AIR after PFBHA treatment.

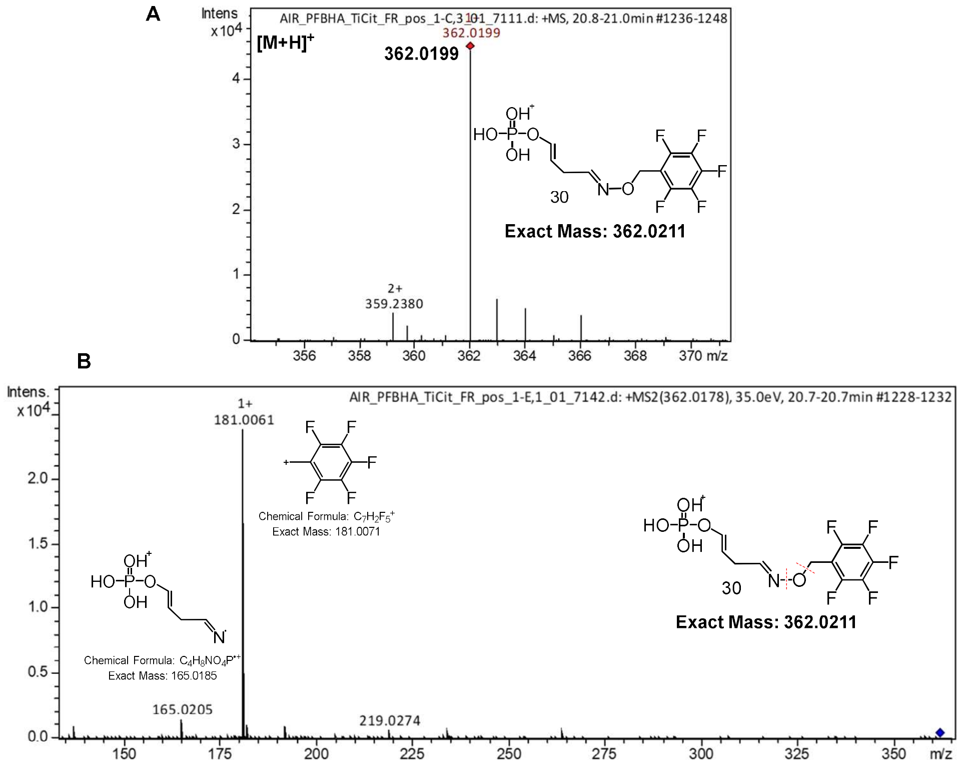

Figure S4. Characterization of **30** formed in the *At*ThiC-catalyzed reaction of AIR after PFBHA treatment. (A) MS analysis of **30** (positive ion mode). (B) MS-MS analysis of **30** (positive ion mode).

Enzymatic assay of *At*ThiC (D383A) with AIR for NMR characterization of 30

Multiple reactions were set up on a 1 mL scale to give sufficient product for analysis using the above conditions (see the section on enzymatic assay of *Arabidopsis thaliana* ThiC, *Caulobacter crescentus* ThiC, and their variants: PFBHA derivatization)). Compound **30** was purified from the reaction mixture by HPLC using method 2 described in the HPLC method section (Line B: 100 mM potassium phosphate, pH=6.6) (Figure S4A). The purified product was concentrated using a freeze-dryer (Labconco). The freeze-dried sample was dissolved in 1 mL of 5mM ammonium acetate (pH=6.6) and purified again using HPLC method 2 (Line B: 5mM ammonium acetate, pH=6.6). The resulting fractions were freeze-dried and dissolved in 250 µL D_2O for NMR analysis.

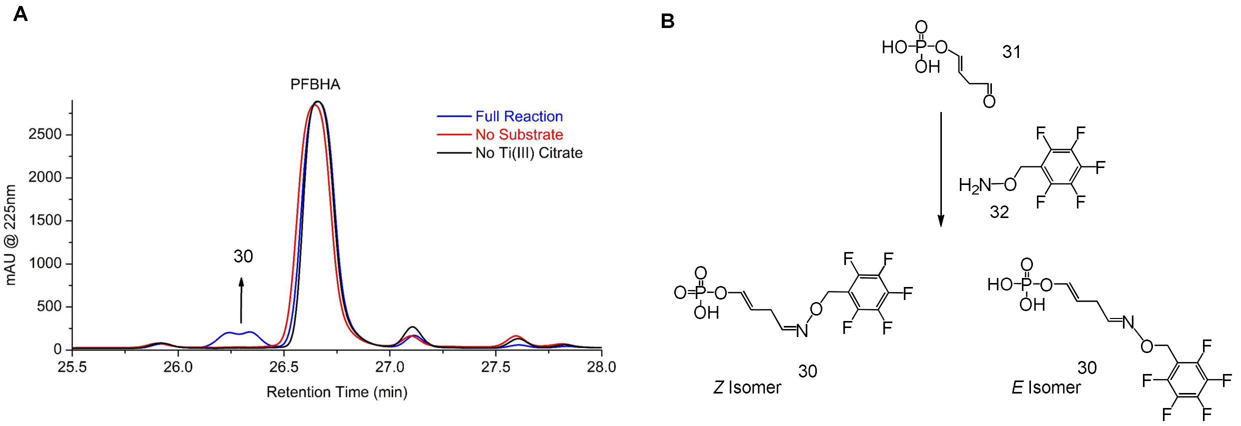

Figure S5. Identification of **30** in the *At*ThiC (D383A) catalyzed reaction of AIR after PFBHA treatment. (A) HPLC chromatogram showing the formation of **30**. The splitting of the peak is due to the presence of *E* and Z isomers of oxime **30**. This peak was collected over multiple runs for NMR characterization. (B) Proposed structures for *E* & *Z* isomers of **30**. **31** is proposed to be the product of the *At*ThiC (D383A) catalyzed reaction, which reacts with PFBHA to form the *E* and Z isomers of oxime **30**.

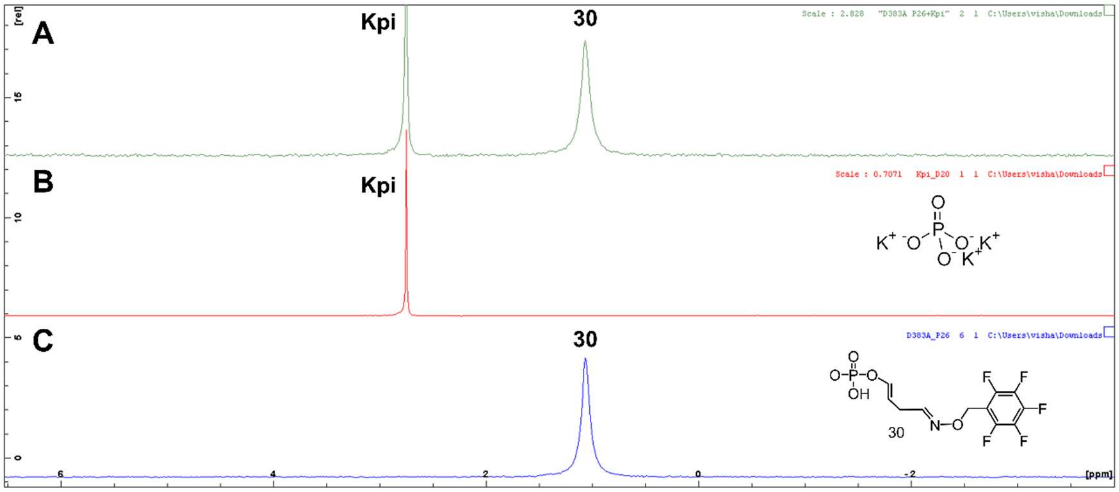

Figure S6. ^{31}P NMR spectra of **30**. (A) Mixture of potassium phosphate (Kpi) and HPLC purified sample of **30**. (B) Potassium phosphate. (C) HPLC purified sample of 30.

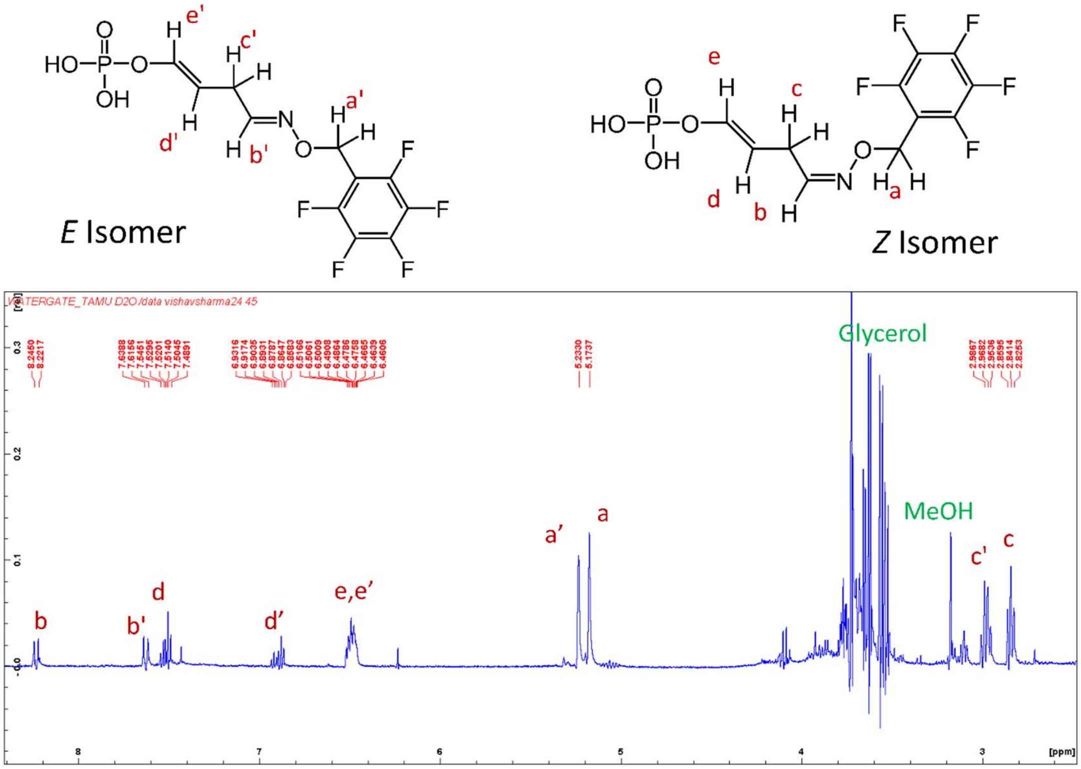

Figure S7. ^{1}H NMR spectra of **30** (^{31}P Coupled).

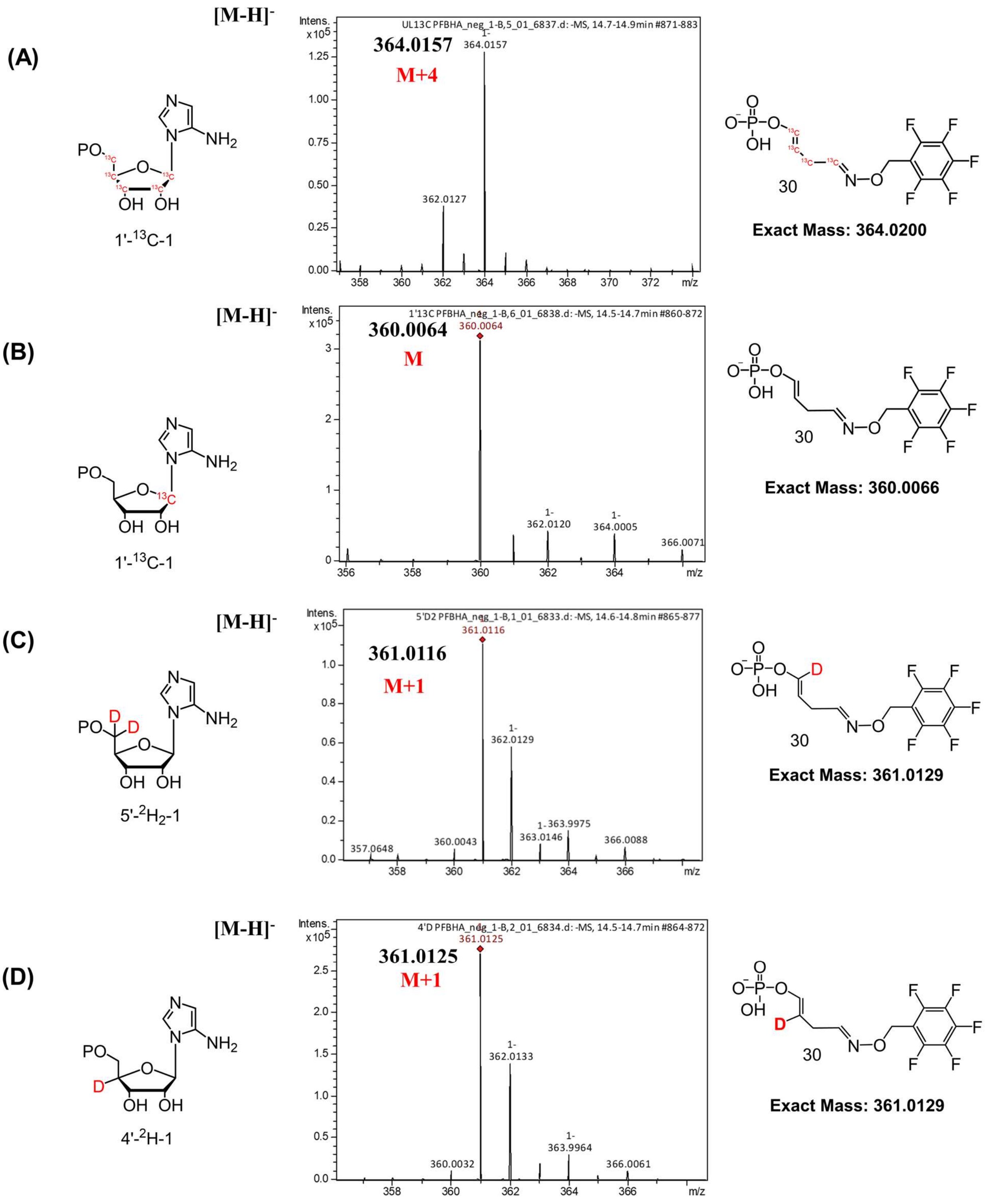
(A)
[M-H]-
UL13C PFBHA_neg_1-B,5_01_6837.d: -MS, 14.7-14.9min #871-883
364.0157
M+4
362.0127
1'-13C-1
30
Exact Mass: 364.0200
(B)
[M-H]-
1'13C PFBHA_neg_1-B,6_01_6838.d: -MS, 14.5-14.7min #860-872
360.0064
M
362.0120
364.0005
366.0071
1'-13C-1
30
Exact Mass: 360.0066
(C)
[M-H]-
5'D2 PFBHA_neg_1-B,1_01_6833.d: -MS, 14.6-14.8min #865-877
361.0116
M+1
362.0129
363.9975
363.0146
357.0648
360.0043
366.0088
5'-2H2-1
30
Exact Mass: 361.0129
(D)
[M-H]-
4'D PFBHA_neg_1-B,2_01_6834.d: -MS, 14.5-14.7min #864-872
361.0125
M+1
362.0133
363.9964
360.0032
366.0061
4'-2H-1
30
Exact Mass: 361.0129

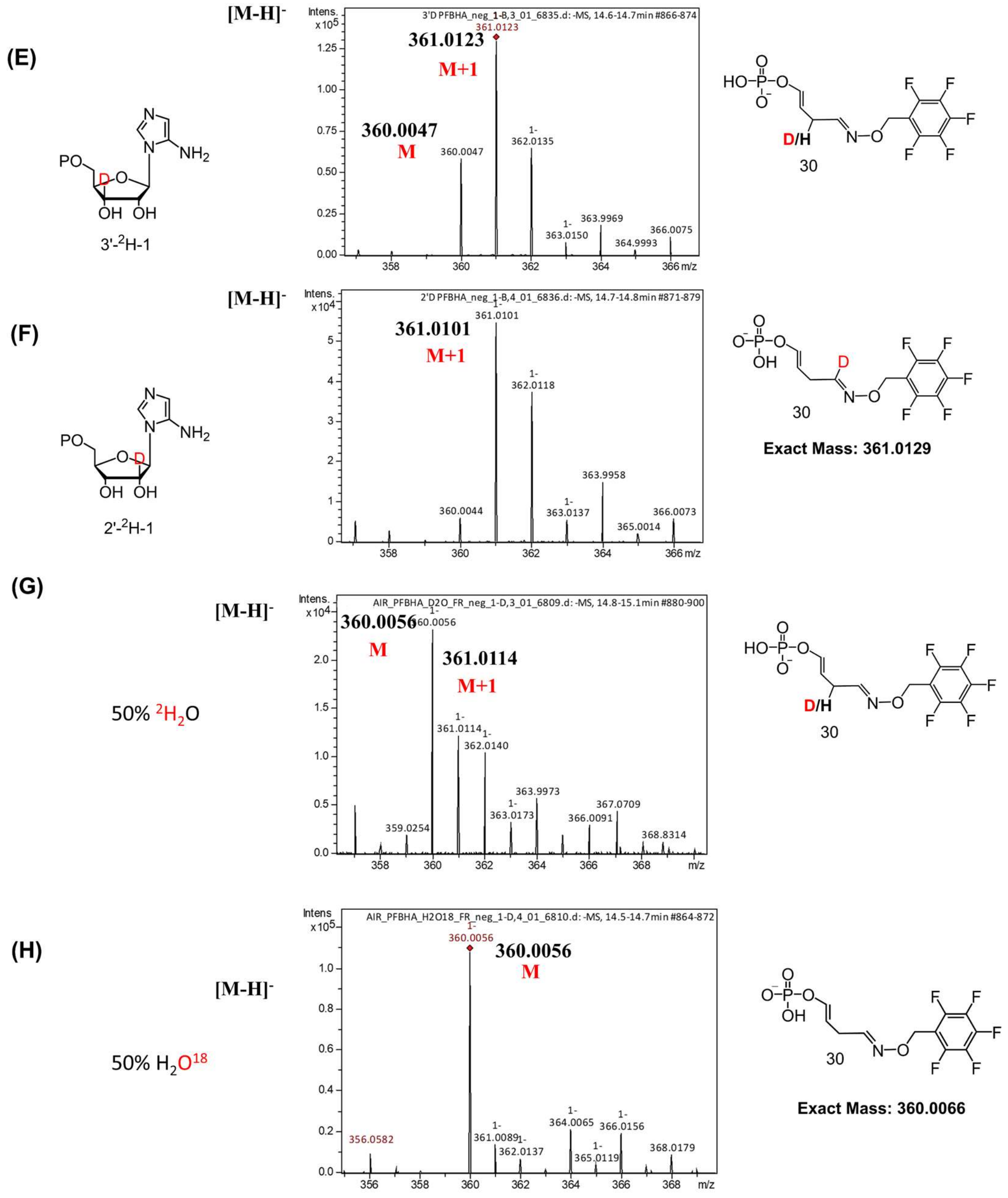

Figure S8. MS analysis of **30** formed upon using AIR (1) isotopologues and labeled solvent in the *At*ThiC-catalyzed reaction. (A) 1',2',3',4',5'-$^{13}C_5$-**1**. (B) 1'-^{13}C-**1**. (C) 5'-$^{2}H_2$-**1**. (D) 4'-^{2}H-**1**. (E) 3'-^{2}H-**1**. (F) 2'-^{2}H-**1**. (G) 50% $^{2}H_2O$. (H) 50% H_2O^{18}. The 362.01 peak, observed consistently across all spectra, indicates the presence of impurity from an unidentified compound, which is also present in the control samples.

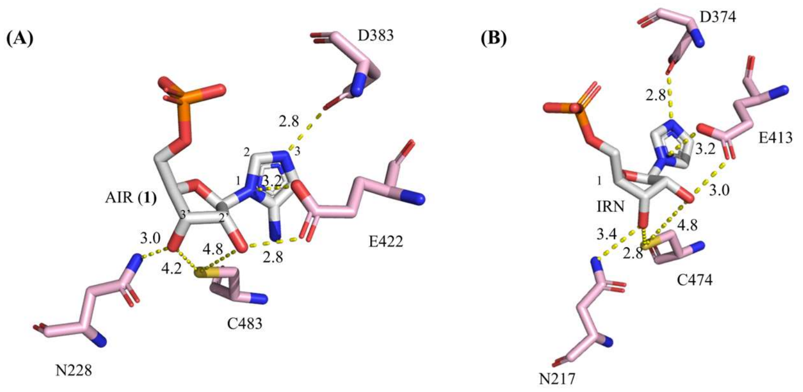

Figure S9. (A) Interactions of AIR with N228, D383, E422, and C483 in the active site of *At*ThiC (PDB:4S28)[8]. (B) Interactions of imidazole ribonucleotide (IRN, 5-desamino AIR) with N217, D374, E413, and C474 in the active site of *Cc*ThiC (PDB:3EPN)[2]. The residue responsible for acid-catalyzed ring opening (**13** to **14**, Figures 3, 5, and 11) has not been identified.

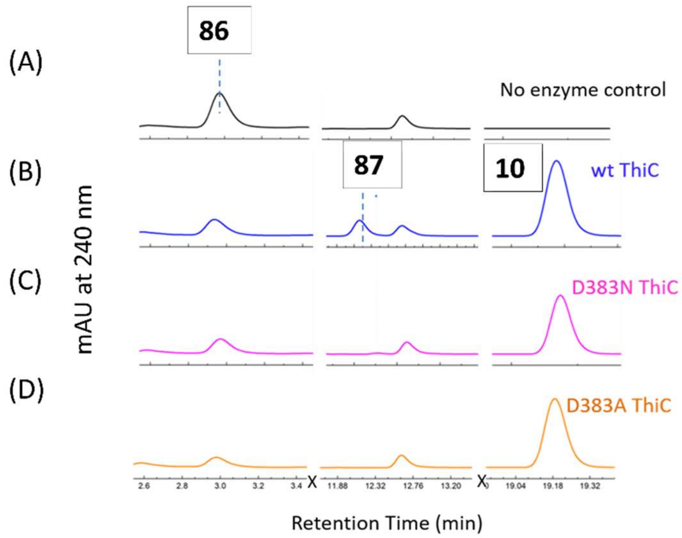

Figure S10. HPLC chromatogram (@240 nm, no PFBHA derivatization) for the reaction of *At*ThiC and its mutants with AIR. (A) No enzyme control. (B) wt *At*ThiC. (C) *At*ThiC (D383N). (D) *At*ThiC (D383A). wt, D383N, and D383A *At*ThiC all consume AIR (detected as **86**) and form 5'-deoxyadenosine **10**. The formation of HMP-P (detected as **87**) is significantly lower in *At*ThiC (D383N), with no formation in *At*ThiC (D383A). The quantitation of these chromatograms is shown in Figure S10.

	AIRs (**86**) Consumed (µM)	HMP (**87**) Formed (µM)	5'-dAdo (**10**) Formed (µM)
wt ThiC	250	70	308
D383N ThiC	200	5	230
D383A ThiC	250	N/A	280

Enzyme concentration=300 µM; AIR concentration= 410 µM;
SAM=1 mM; Ti (III) Citrate=6 mM

Figure S11. Quantitation of HPLC chromatograms shown in Figure S9.

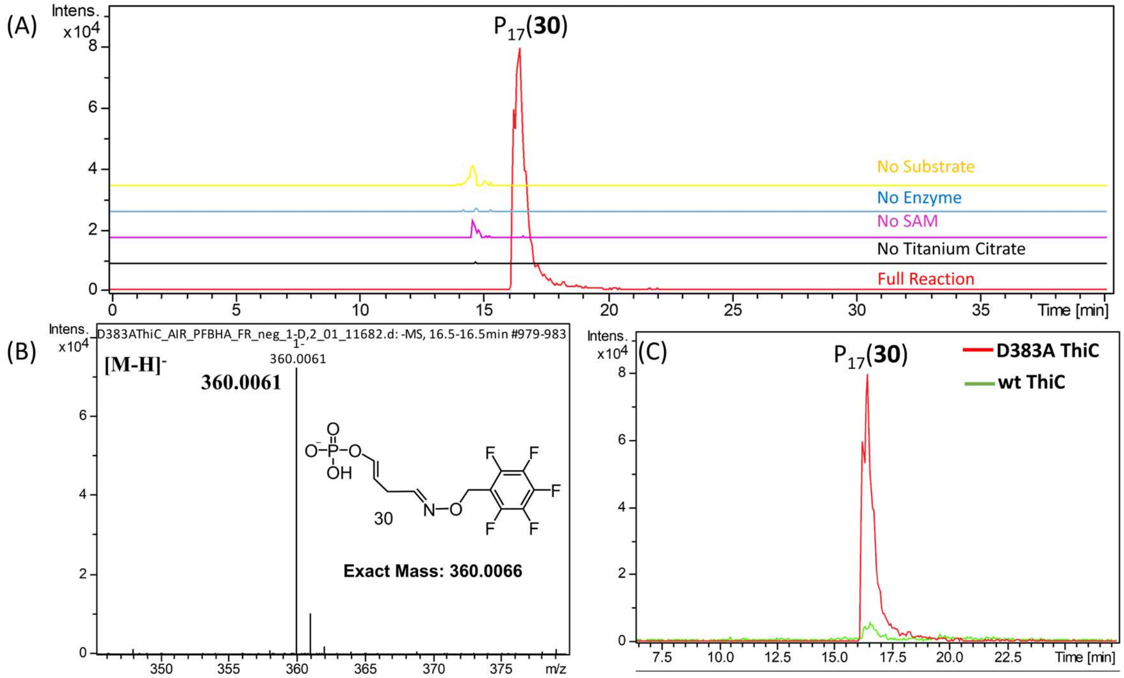

Figure S12. LC-MS analysis of **30** formed in the *At*ThiC (D383A) catalyzed reaction of AIR after PFBHA treatment. (A) EIC of **30** (P_{17}). (B) MS analysis of **30**. (C) Overlaid EIC of **30** formed in the wt *At*ThiC and *At*ThiC (D383A) catalyzed reactions. The formation of **30** in the *At*ThiC (D383A) catalyzed reaction is significantly higher than the wt *At*ThiC-catalyzed reaction.

Quantitation of shunt product (30) in the *At*ThiC (D383A) catalyzed reaction

To quantify **30**, a commercial standard of formaldehyde PFBHA oxime **88** was used to derive a calibration curve (Figure S12). Based on the calibration curve, the amount of **30** formed in the *At*ThiC (D383A) catalyzed reaction was approximately 32 μM. The ratio of **30**:5'-dA is approximately 1:9. Reaction conditions: Enzyme=300 μM, AIR= 410 μM, SAM= 1mM, Ti (III) citrate= 6mM. The amount of AIR consumed was 250 μM and the amount of 5'-dA formed was 280 μM.

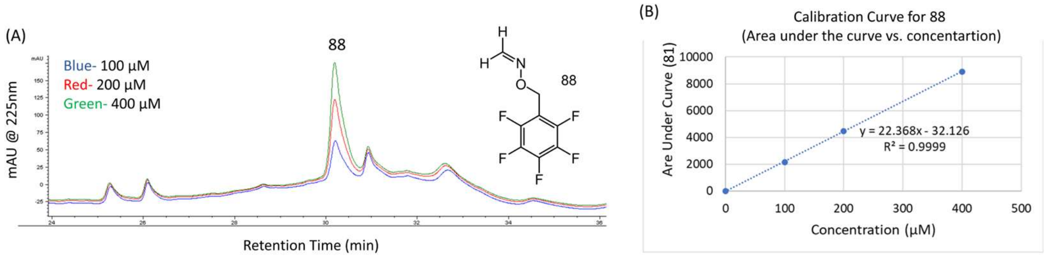

Figure S13. Quantitation of shunt product **30** in the *At*ThiC (D383A) catalyzed reaction. (A) HPLC traces for different concentrations of the formaldehyde PFBHA oxime **86**. (B) Calibration curve for **86**.

Procedure for DEPT-90 NMR of the *At*ThiC (D383A) catalyzed reaction with 1'-^{13}C-AIR

For the DEPT90-NMR analysis of the enzymatic reaction, *At*ThiC (D383A) was desalted twice using a Bio-spin 6 column (Bio-Rad) into 100 mM phosphate buffer, pH 7.5 (to remove glycerol from the storage buffer). The components of the enzymatic reaction (400 μL) were 100 mM phosphate buffer, pH 7.5, 400 μM *At*ThiC (D383A), chemically synthesized 1'-^{13}C-AIR (2 mM),[6] S-adenosylmethionine (SAM, 1 mM), and sodium dithionite (2 mM). The reaction mixture was allowed to proceed anaerobically for about 6 hours, diluted with 10% D_2O, and transferred to the NMR tube for analysis.

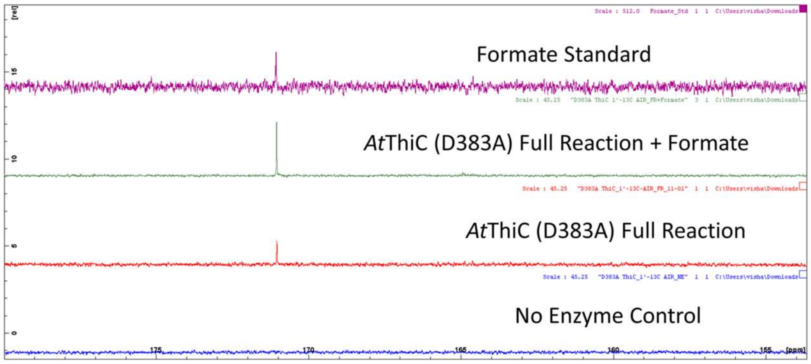

Figure S14. DEPT-90-NMR analysis of the *At*ThiC (D383A) catalyzed reaction with 1'-^{13}C-AIR showing the formation of ^{13}C formate (171 ppm).

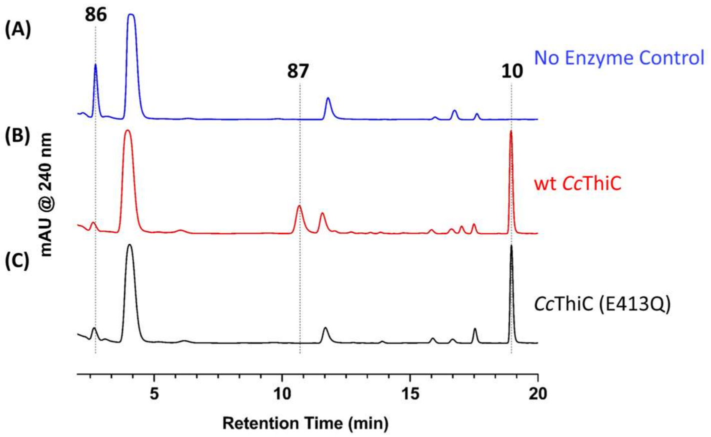

Figure S15. HPLC chromatogram (@240 nm, no PFBHA derivatization) of wt *Cc*ThiC and *Cc*ThiC (E413Q) catalyzed reactions of AIR. (A) No enzyme control. (B) wt *Cc*ThiC. (C) *Cc*ThiC (E413Q). *Cc*ThiC (E413Q) consumes AIR (detected as **86**) but does not form HMP-P (detected as **87**).

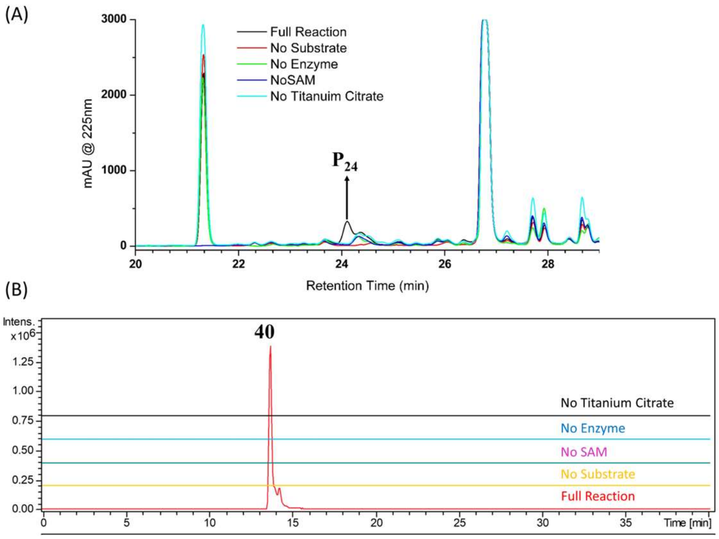

Figure S16. Characterization of **40** in the *Cc*ThiC (E413Q) catalyzed reaction of AIR after PFBHA treatment. (A) HPLC chromatogram showing the formation of P_{24}(**40**). (B) EIC (LC-MS) of **40**. The difference in retention time on LC-MS is because of different C-18 columns and flow rates used in LC-MS and HPLC analysis. The *E* and *Z* isomers are inseparable by HPLC but separable by LC-MS.

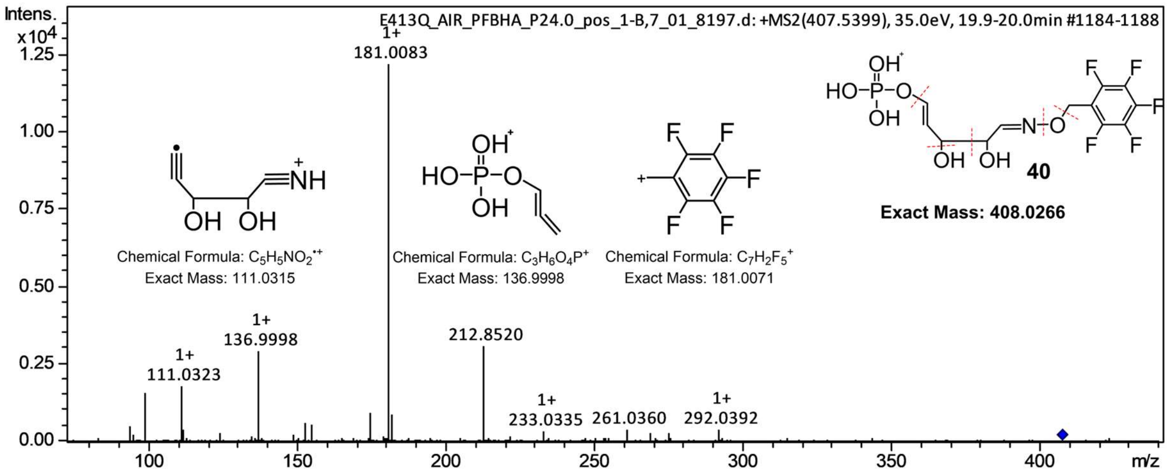

Figure S17. MS-MS of **40** in positive ion mode.

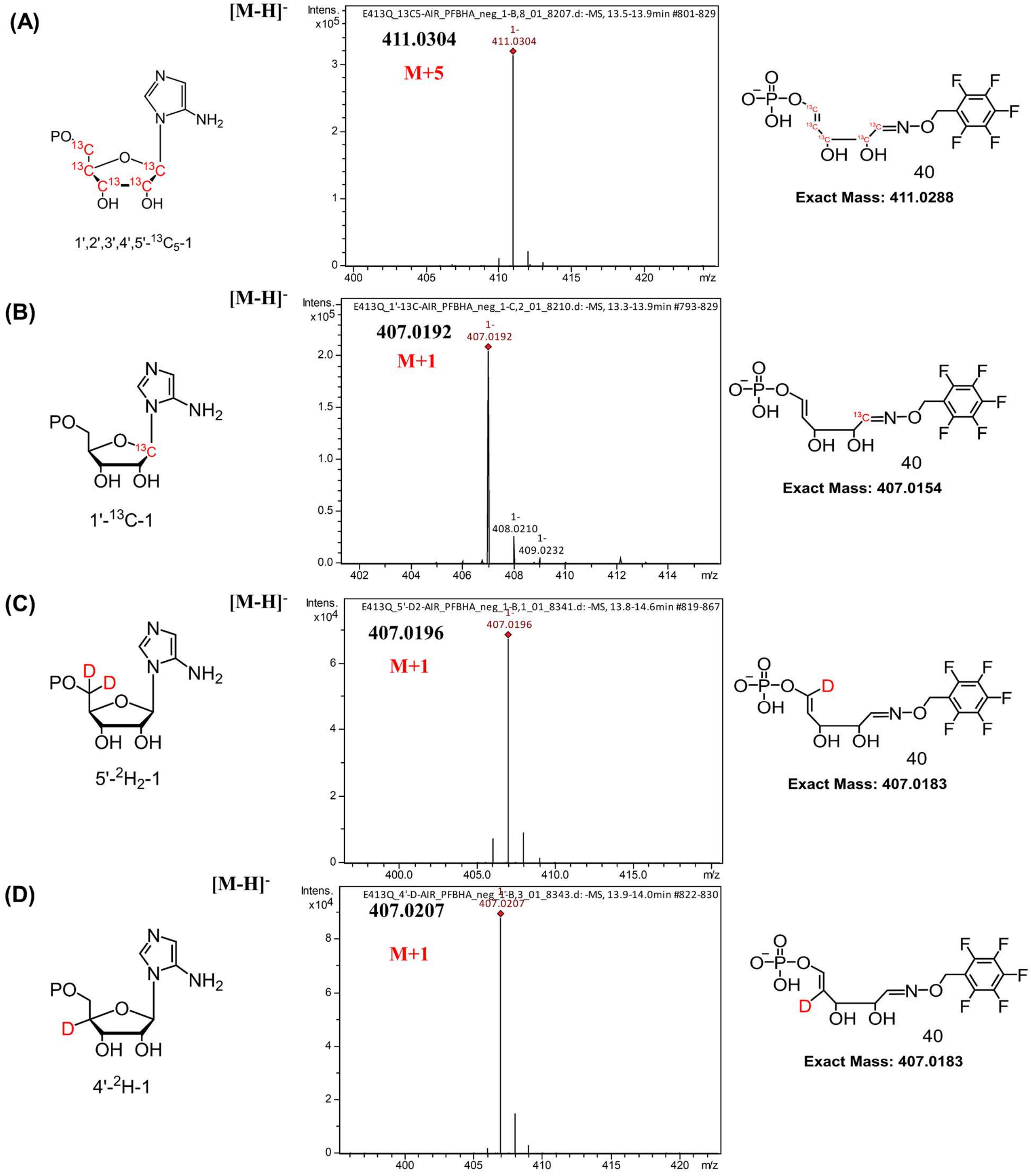
(A)
1',2',3',4',5'-13C5-1
[M-H]-
Intens. x10^5
E413Q_13C5-AIR_PFBHA_neg_1-B,8_01_8207.d: -MS, 13.5-13.9min #801-829
411.0304
M+5
1- 411.0304
m/z
40
Exact Mass: 411.0288
(B)
1'-13C-1
[M-H]-
Intens. x10^5
E413Q_1'-13C-AIR_PFBHA_neg_1-C,2_01_8210.d: -MS, 13.3-13.9min #793-829
407.0192
M+1
1- 407.0192
1- 408.0210
1- 409.0232
m/z
40
Exact Mass: 407.0154
(C)
5'-2H2-1
[M-H]-
Intens. x10^4
E413Q_5'-D2-AIR_PFBHA_neg_1-B,1_01_8341.d: -MS, 13.8-14.6min #819-867
407.0196
M+1
1- 407.0196
m/z
40
Exact Mass: 407.0183
(D)
4'-2H-1
[M-H]-
Intens. x10^4
E413Q_4'-D-AIR_PFBHA_neg_1-B,3_01_8343.d: -MS, 13.9-14.0min #822-830
407.0207
M+1
1- 407.0207
m/z
40
Exact Mass: 407.0183

(E)

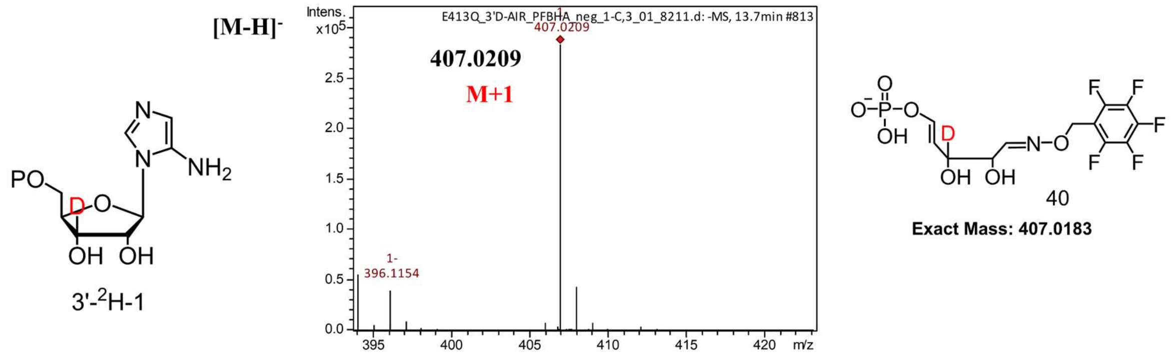
[M-H]-
E413Q_3'D-AIR_PFBHA_neg_1-C,3_01_8211.d: -MS, 13.7min #813
407.0209
M+1
396.1154
3'-2H-1
40
Exact Mass: 407.0183

(F)

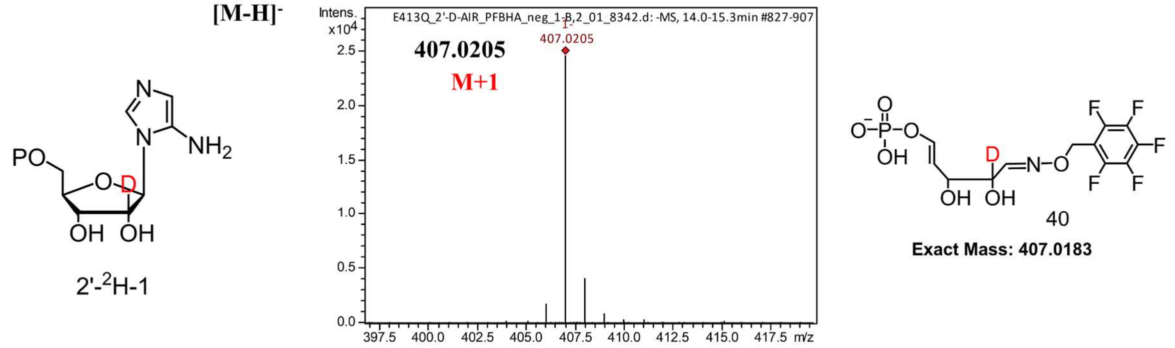
[M-H]-
E413Q_2'-D-AIR_PFBHA_neg_1-B,2_01_8342.d: -MS, 14.0-15.3min #827-907
407.0205
M+1
2'-2H-1
40
Exact Mass: 407.0183

(G)

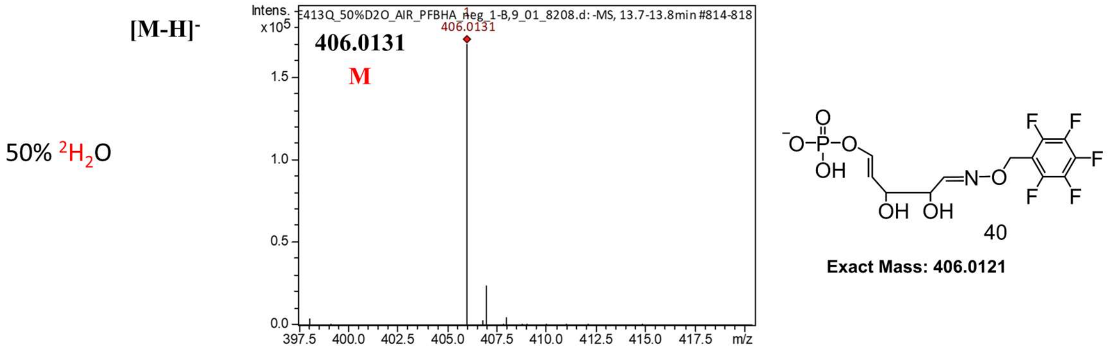
[M-H]-
E413Q_50%D2O_AIR_PFBHA_neg_1-B,9_01_8208.d: -MS, 13.7-13.8min #814-818
406.0131
M
50% 2H2O
40
Exact Mass: 406.0121

(H)

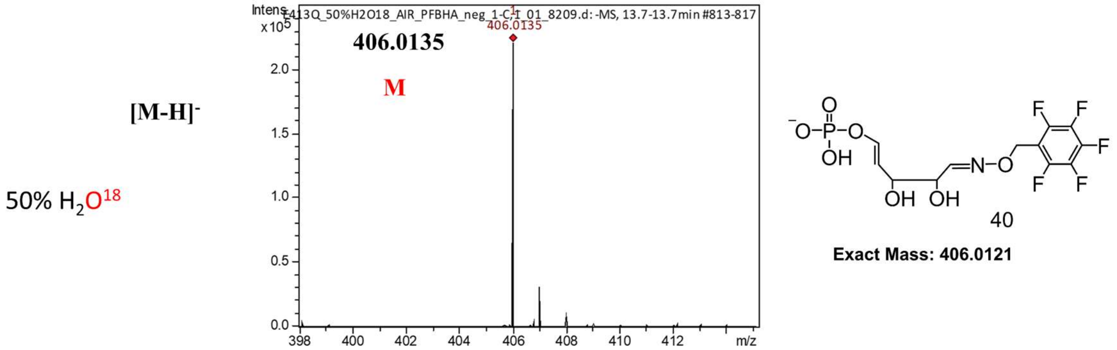
[M-H]-
E413Q_50%H2O18_AIR_PFBHA_neg_1-C,1_01_8209.d: -MS, 13.7-13.7min #813-817
406.0135
M
50% H2O18
40
Exact Mass: 406.0121

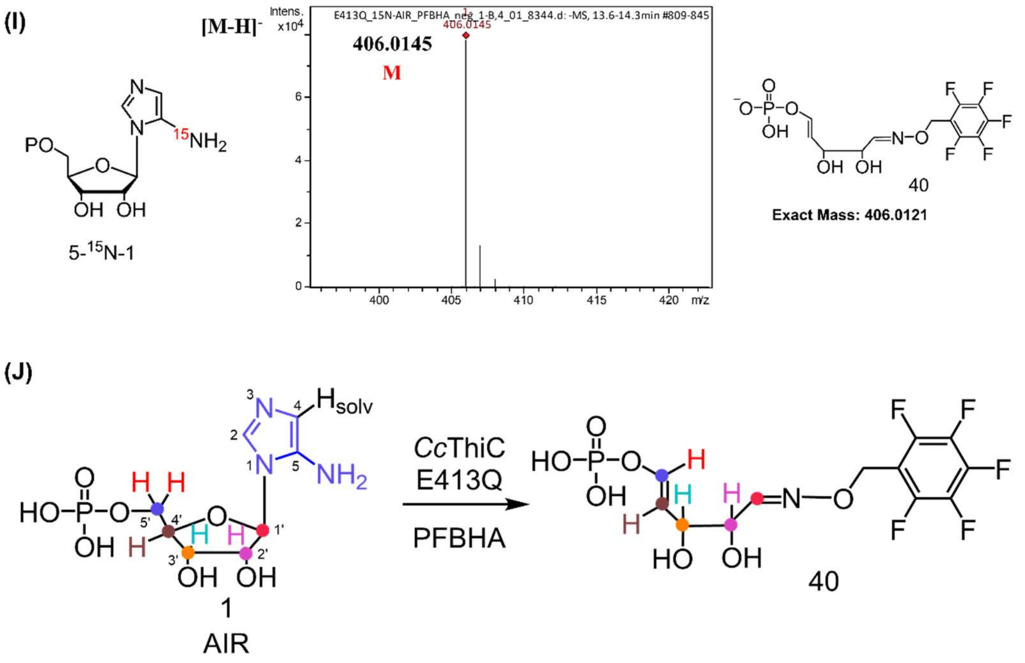

Figure S18. MS analysis of **40** formed upon using AIR (**1**) isotopologues and labeled solvent in the *Cc*ThiC (E413Q) catalyzed reaction. (A) 1',2',3',4',5'-$^{13}C_5$-**1**. (B) 1'-^{13}C-**1**. (C) 5'-$^2H_2$-**1**. (D) 4'-^2H-**1**. (E) 3'-^2H-**1**. (F) 2'-^2H-**1**. (G) 50% ^2H_2O. (H) 50% H_2O^{18}. (I) 5-^{15}N-**1**. (J) Summary of labeling studies.

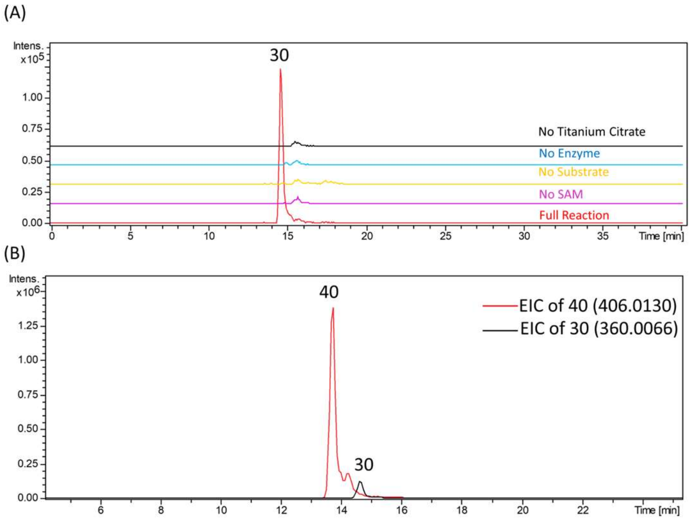

Figure S19. Characterization of **30** in the *Cc*ThiC (E413Q) catalyzed reaction of AIR after PFBHA treatment. (A) EIC of **30**. (B) Overlaid EIC of **40** and **30** to compare the relative ratio of the two shunt products.

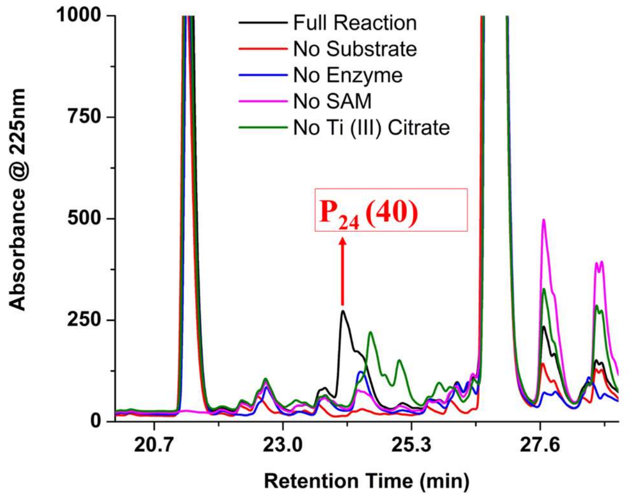

Figure S20. HPLC chromatogram of the *At*ThiC (E422Q) catalyzed reaction of AIR after PFBHA treatment showing formation of P_{24} (**40**).

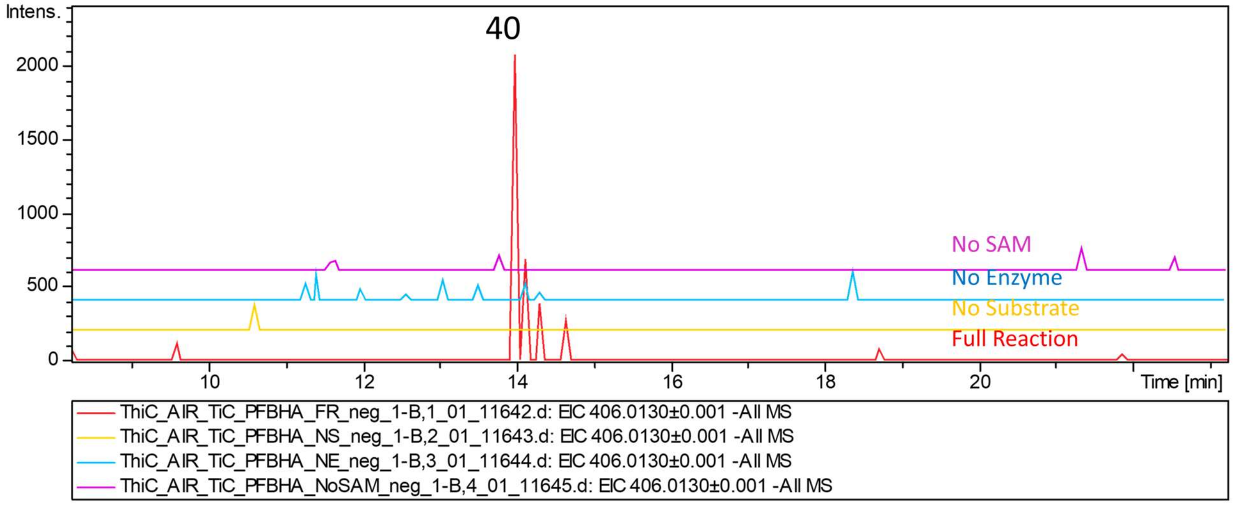

Figure S21. EIC of **40** in the wt *Cc*ThiC-catalyzed reaction of AIR after PFBHA treatment.

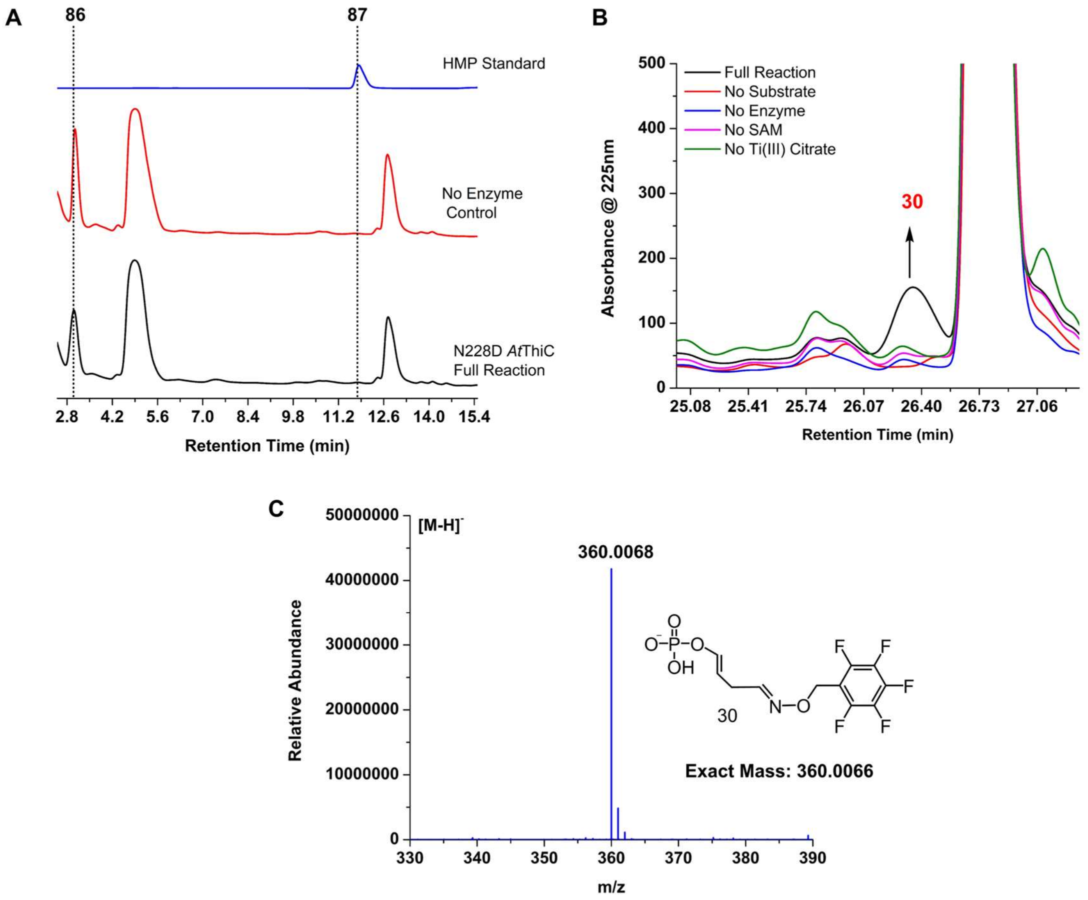

Figure S22. Studies on *At*ThiC (N228D) (A) HPLC chromatogram (@240 nm, no PFBHA derivatization) of the *At*ThiC (N228D) catalyzed reaction of AIR showing consumption of AIR (detected as **86**) but no HMP-P (detected as **87**) formation. (B) HPLC chromatogram (@ 225nm) of *At*ThiC (N228D) catalyzed reaction of AIR after PFBHA treatment showing formation of **30** in full reaction only. (C) MS analysis of **30** formed in the *At*ThiC (N228D) catalyzed reaction of AIR after PFBHA treatment.

Synthesis of substrate analog 43

89 was synthesized as previously described.[9] Its NMR spectrum matching the reported ^{1}H-NMR is shown in Figure S22. **89** was enzymatically phosphorylated to **43** using AIRs Kinase and HPLC purified for use in the ThiC reaction. (For reaction conditions, see the section on Enzymatic phosphorylation of AIRs and its isotopologues).

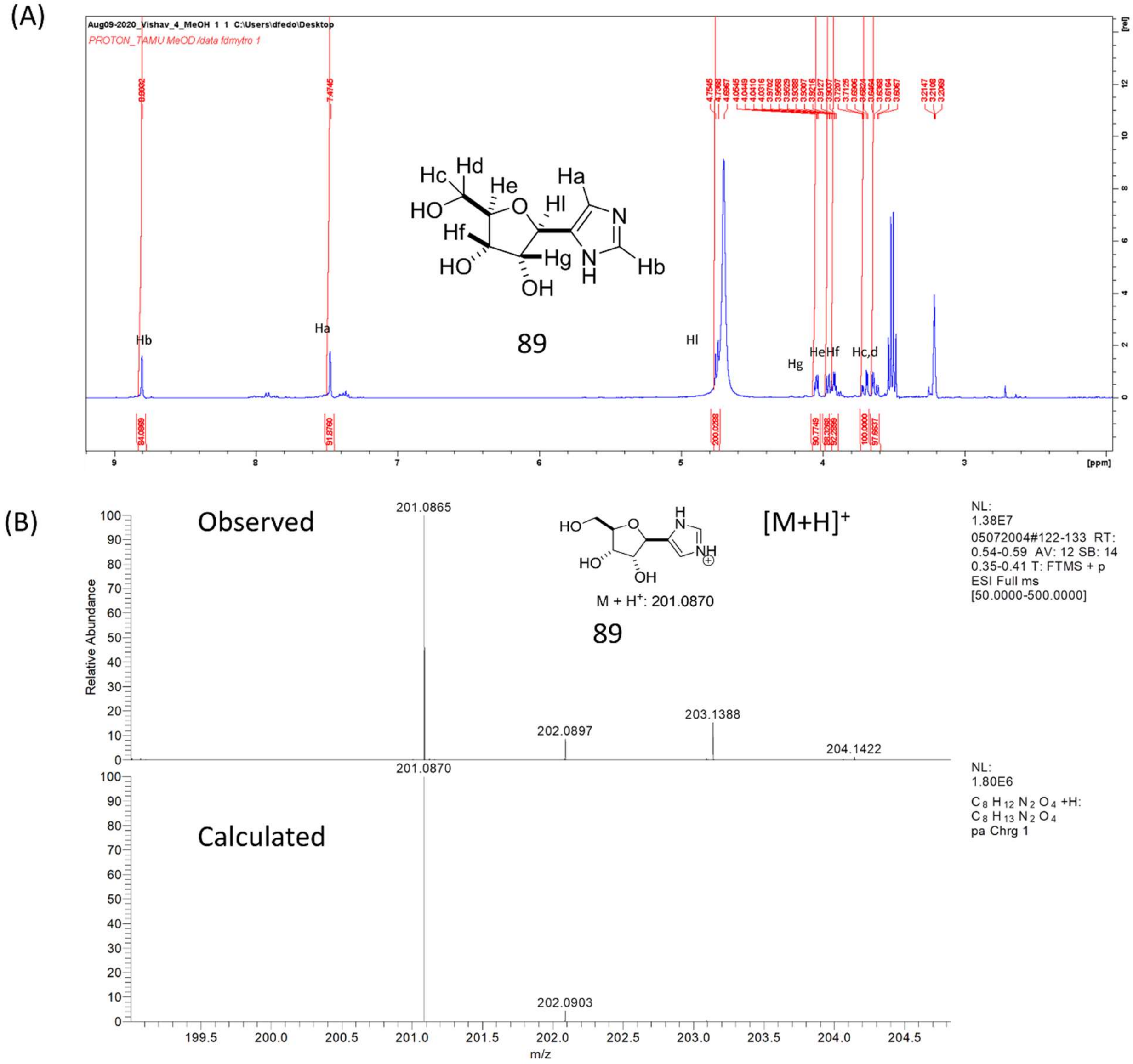

Figure S23. Characterization of **89**. (A) ^{1}H-NMR of **89**. (B) ESI-MS of **89** in positive ion mode.

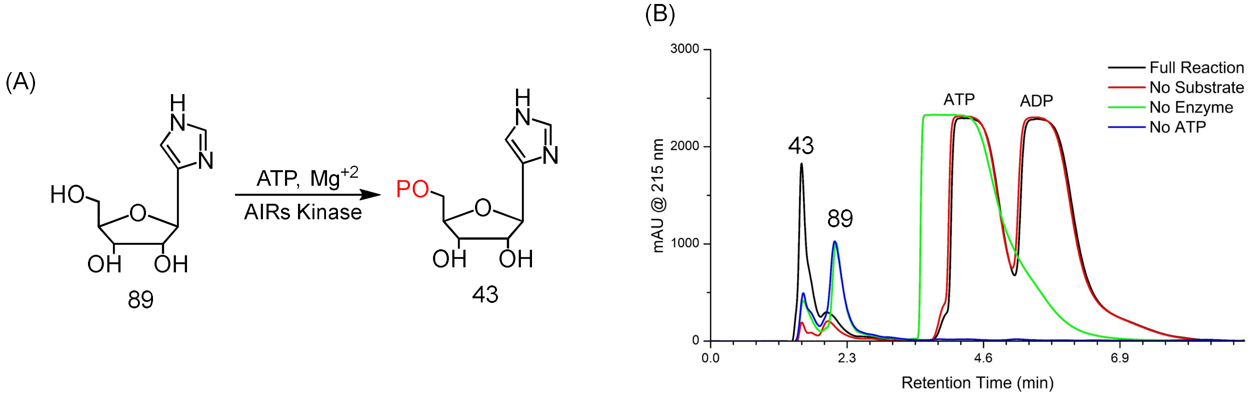

Figure S24. Enzymatic phosphorylation of **89**. (A) Scheme for the enzymatic phosphorylation. (B) HPLC chromatogram for the phosphorylation reaction.

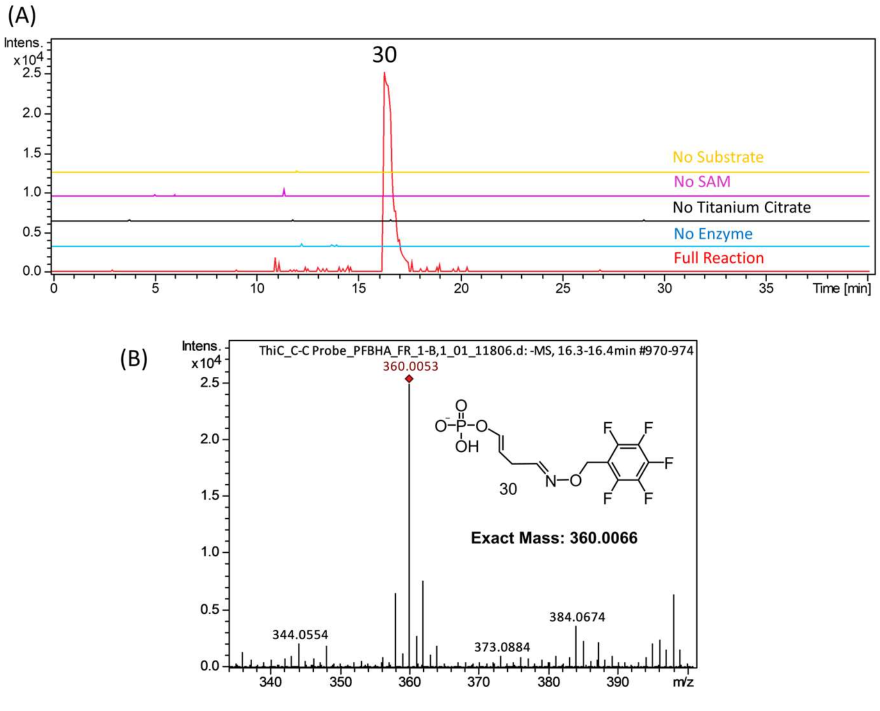

Figure S25. LC-MS analysis of the *At*ThiC catalyzed reaction of **43** after PFBHA treatment. (A) EIC of **30**. (B) MS analysis of **30** (negative ion mode).

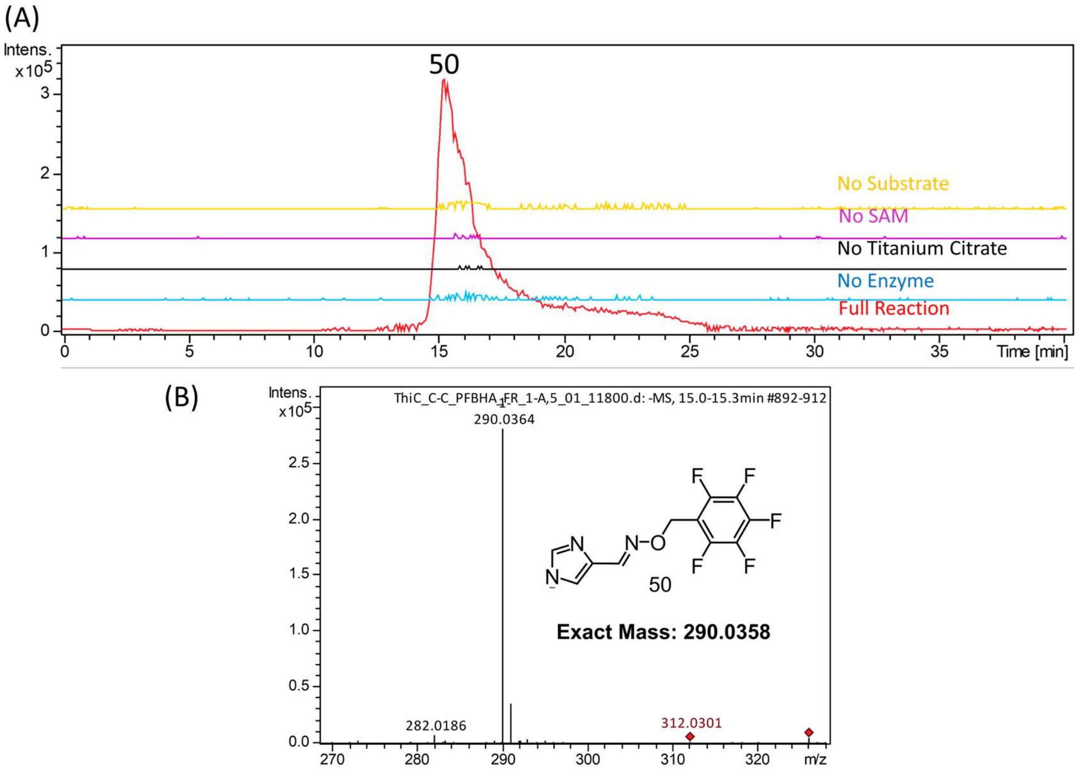

Figure S26. LC-MS analysis of the *At*ThiC catalyzed reaction of **43** after PFBHA treatment. (A) EIC of **50**. (B) MS analysis of **50** (negative ion mode).

Synthesis of authentic sample of 50

100 mg of **49** (1 mmol) was dissolved in 5 mL of 100 mM potassium phosphate (Kpi) buffer, pH=7.5. To this solution, 221 mg of PFBHA (0.88 mmol) was added and the reaction mixture was stirred at 60 °C for 2 hours. **50** precipitated and was filtered and dried for further analysis.

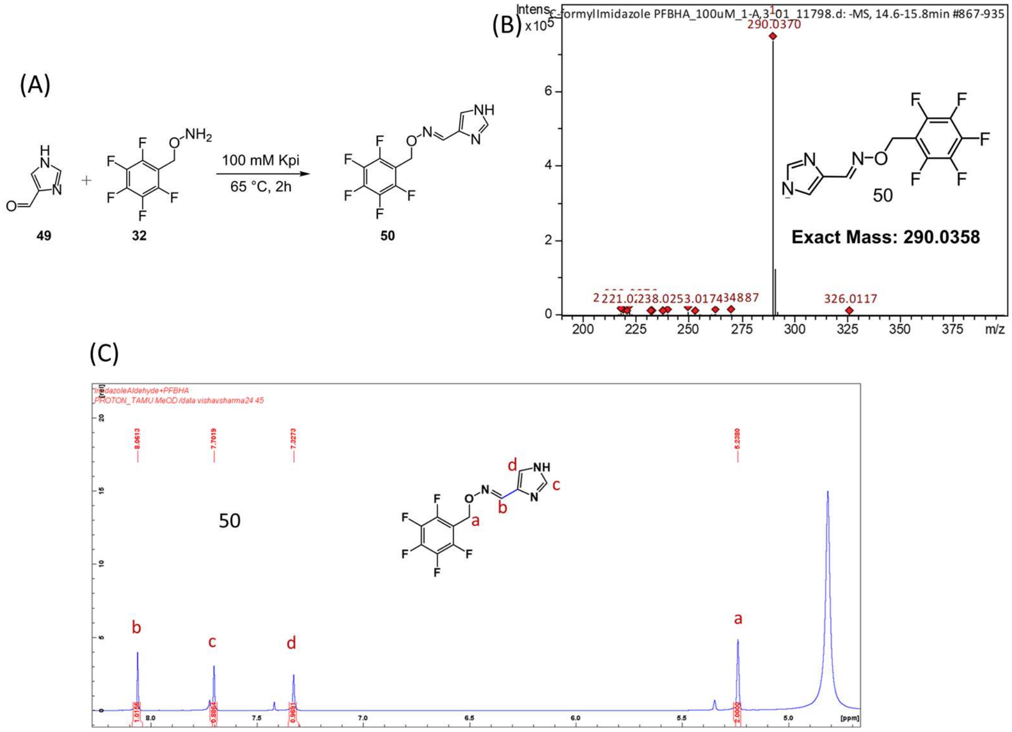

Figure S27. Synthesis of **50**. (A) Scheme for the synthesis of **50**. (B) MS analysis of **50** (negative ion mode). (C) ^{1}H-NMR of **50**.

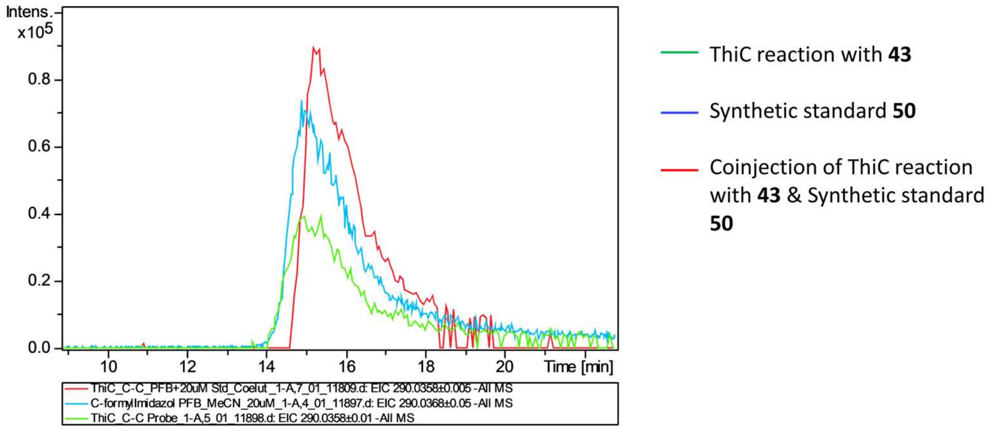

Figure S28. EIC showing the coinjection of the *At*ThiC-catalyzed reaction of **43** with the synthetic standard **50**.

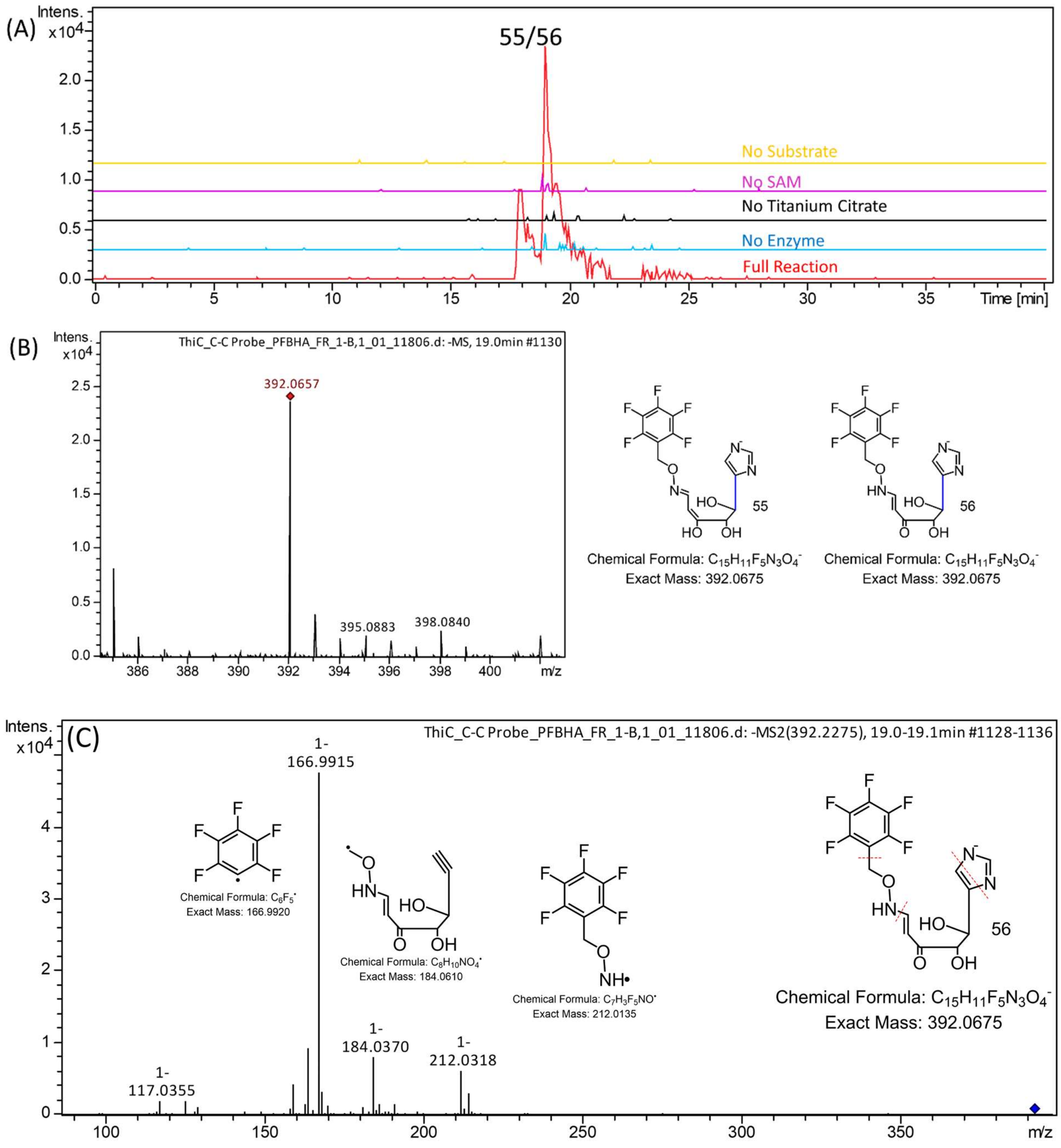
(A)
Intens. x10^4
55/56
No Substrate
No SAM
No Titanium Citrate
No Enzyme
Full Reaction
Time [min]
(B)
ThiC_C-C Probe_PFBHA_FR_1-B,1_01_11806.d: -MS, 19.0min #1130
392.0657
395.0883
398.0840
m/z
55
56
Chemical Formula: C15H11F5N3O4-
Exact Mass: 392.0675
Chemical Formula: C15H11F5N3O4-
Exact Mass: 392.0675
(C)
ThiC_C-C Probe_PFBHA_FR_1-B,1_01_11806.d: -MS2(392.2275), 19.0-19.1min #1128-1136
1- 166.9915
1- 184.0370
1- 212.0318
1- 117.0355
Chemical Formula: C6F5•
Exact Mass: 166.9920
Chemical Formula: C8H10NO4•
Exact Mass: 184.0610
Chemical Formula: C7H3F5NO•
Exact Mass: 212.0135
56
Chemical Formula: C15H11F5N3O4-
Exact Mass: 392.0675
m/z

(D)

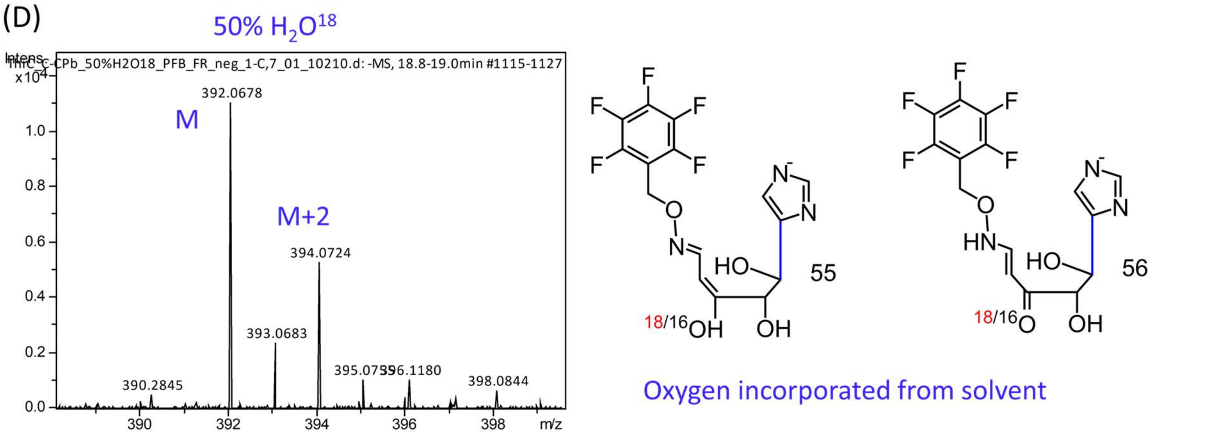

(E)

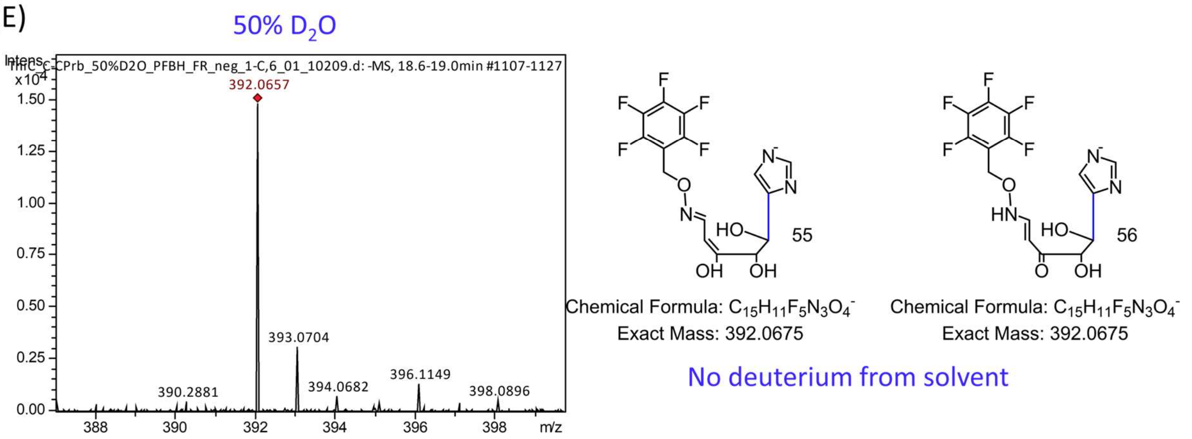

Figure S29. Characterization of **55**/**56** formed in the *At*ThiC catalyzed reaction of **43** after PFBHA treatment. (A) EIC of **55**/**56**. (B) MS analysis of **55**/**56**. (C) MS-MS analysis of **56**. (D) MS analysis of **55**/**56** after performing the reaction in 50% H_2O^{18} buffer showed an O^{18} incorporation in **55**/**56**. (E) MS analysis of **55**/**56** after performing the reaction in 50% D_2O buffer showed no solvent-exchangeable protons in **55**/**56**.

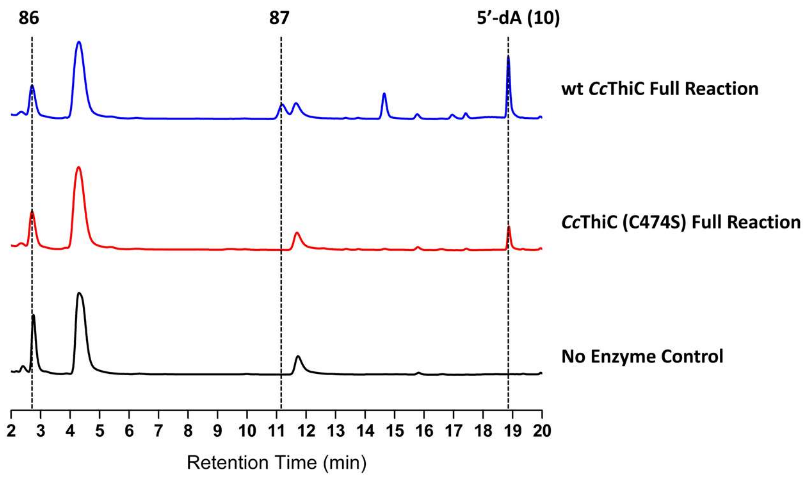

Figure S30. HPLC chromatogram (@240 nm, no PFBHA derivatization) of the *Cc*ThiC (C474S) catalyzed reaction of AIR. AIR (detected as **86**) is consumed in the *Cc*ThiC (C474S), but HMP-P (detected as **87**) is not formed.

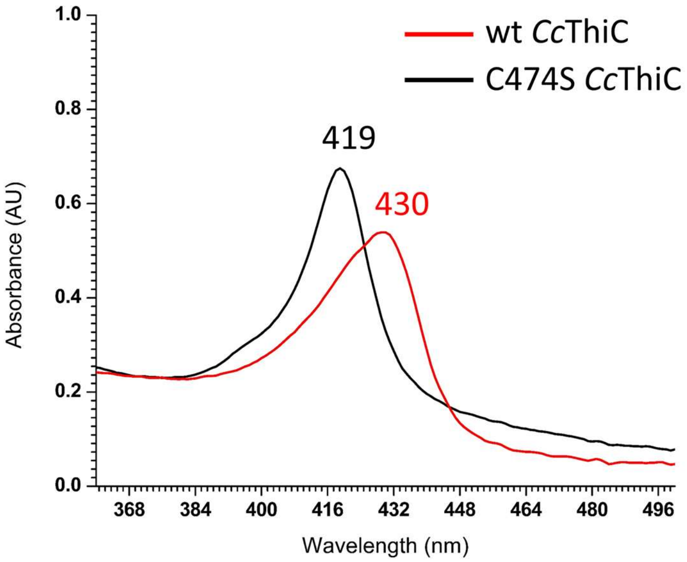

Figure S31. Hemoglobin assay for carbon monoxide detection. The red trace shows the wt *Cc*ThiC-catalyzed reaction of AIR in the presence of hemoglobin (50 μM), producing carboxyhemoglobin (430 nm). The black trace shows the *Cc*ThiC (C474S) catalyzed reaction of AIR in the presence of hemoglobin with only deoxyhemoglobin present (419 nm) and no carboxyhemoglobin production. A previously reported procedure was used for this assay.[6]

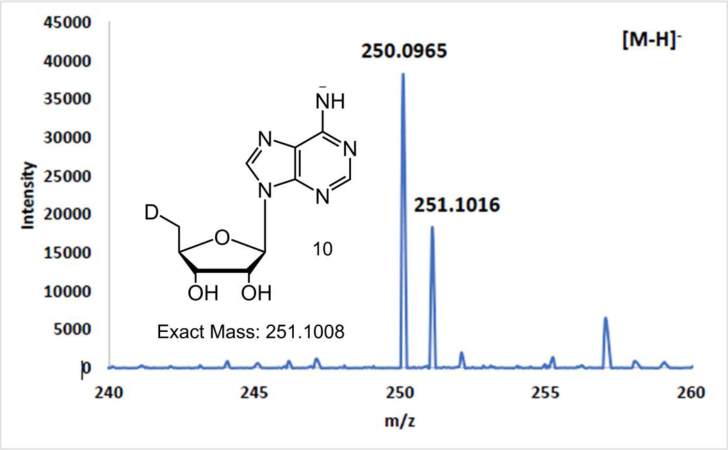

Figure S32. LC-MS analysis of the *Cc*ThiC (C474S) catalyzed reaction with 4'-^{2}H-AIR showing deuterium incorporation in 5'-dA **10** ([M-H]$^-$:251.1016). The [M-H]$^-$ of 250.09 observed is unlabeled 5'-dA formed due to unproductive hydrogen atom abstraction by 5'-dA radical.

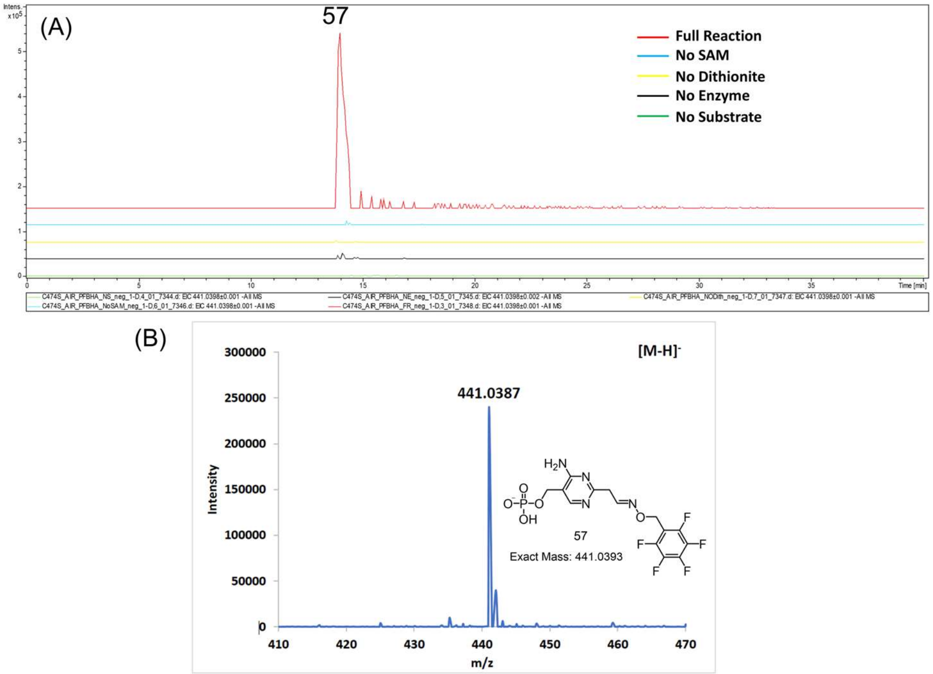

Figure S33. LC-MS of **57** formed in the *Cc*ThiC (C474S) reaction (negative ion mode). (A) EIC of **57**. (B) [M-H]$^-$ for **57**.

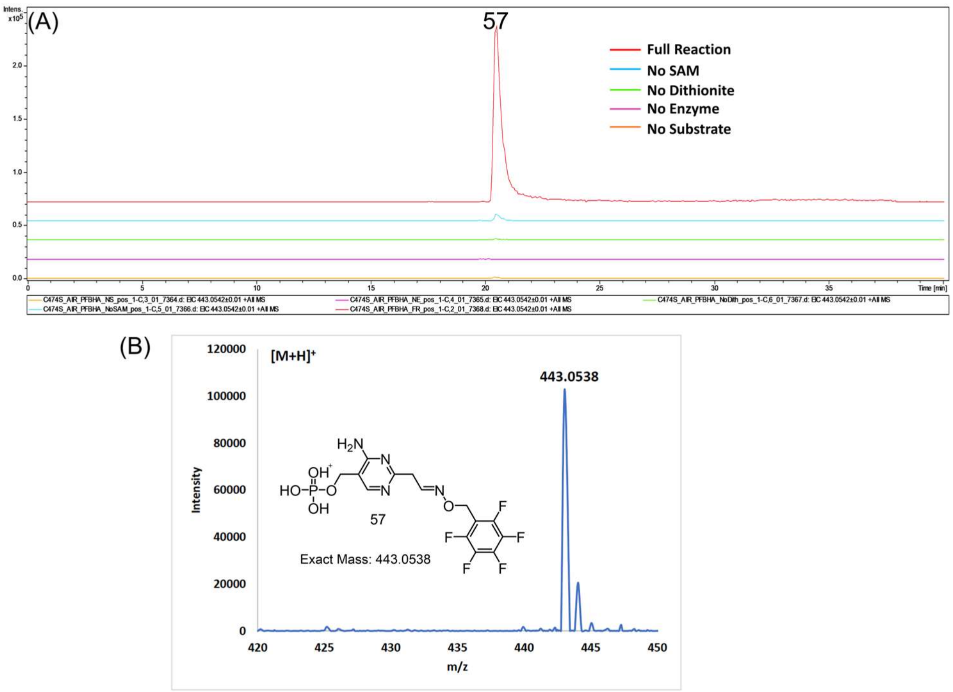

Figure S34. LC-MS of **57** formed in the *Cc*ThiC (C474S) reaction (positive ion mode). (A) EIC of **57**. (B) $[M+H]^+$ for **57**.

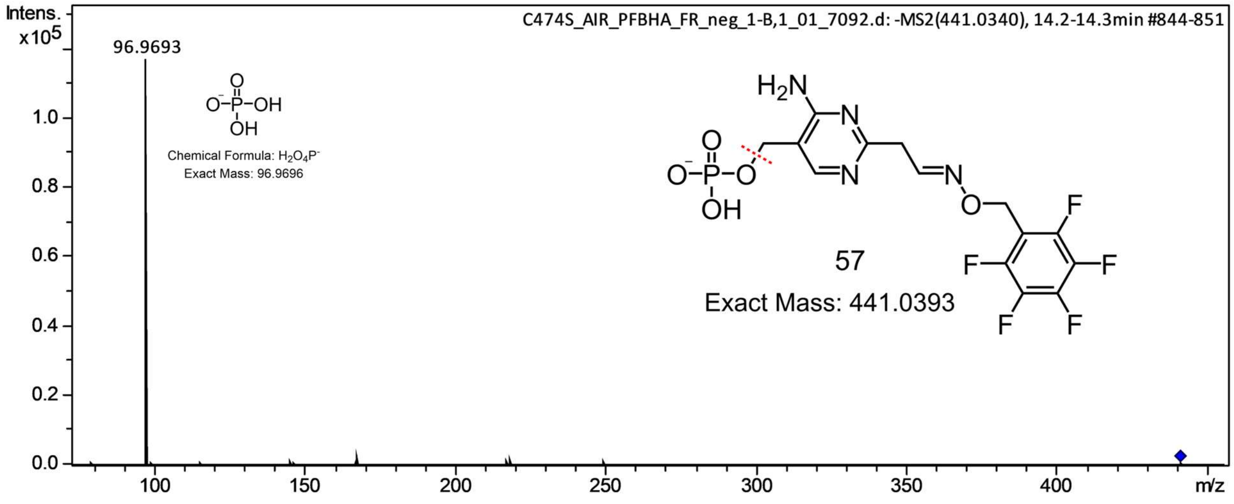

Figure S35. MS-MS analysis of **57** in negative ion mode.

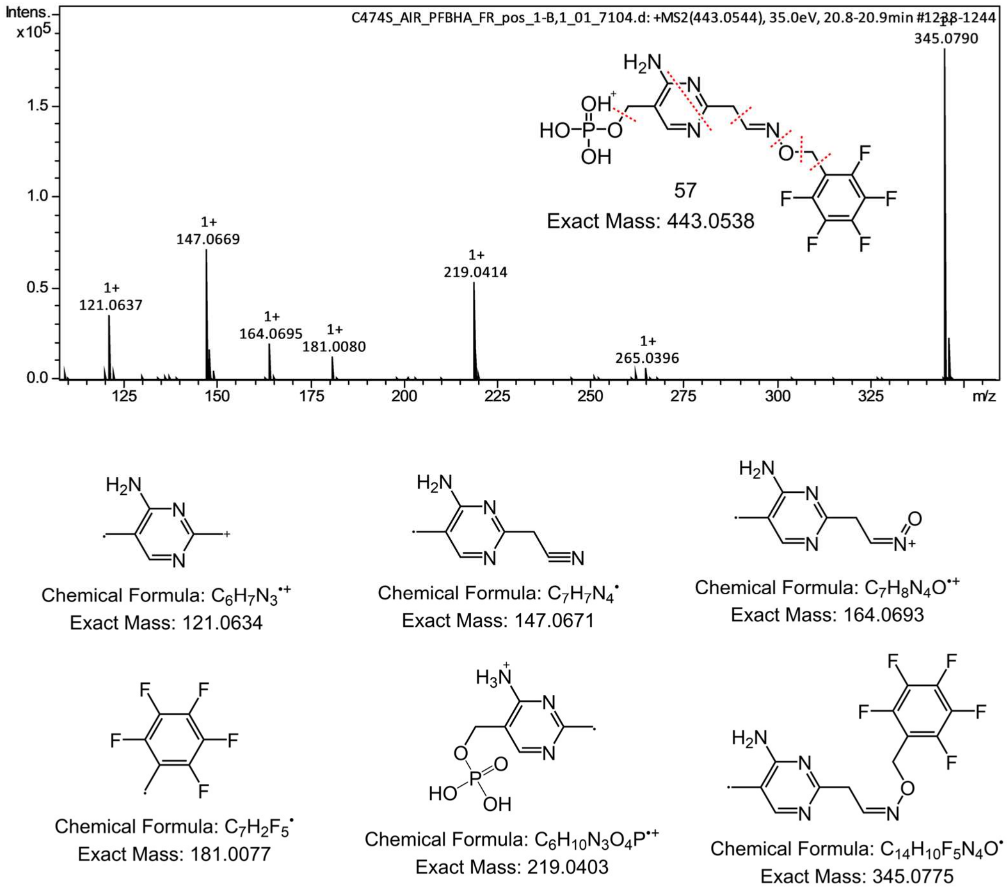

Figure S36. MS-MS analysis of **57** in positive ion mode.

(A)

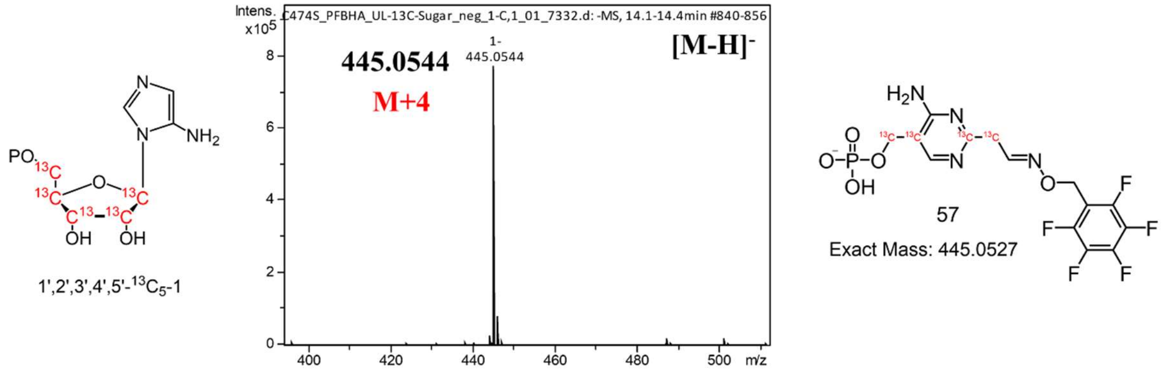

C474S_PFBHA_UL-13C-Sugar_neg_1-C,1_01_7332.d: -MS, 14.1-14.4min #840-856
Intens. x10^5
445.0544
M+4
[M-H]^-
1- 445.0544
m/z
1',2',3',4',5'-13C5-1
57
Exact Mass: 445.0527

(B)

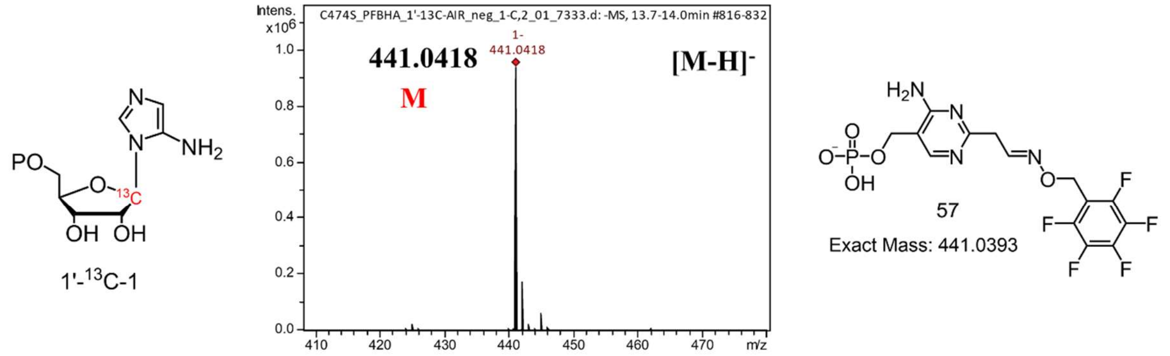

C474S_PFBHA_1'-13C-AIR_neg_1-C,2_01_7333.d: -MS, 13.7-14.0min #816-832
Intens. x10^6
441.0418
M
[M-H]^-
1- 441.0418
m/z
1'-13C-1
57
Exact Mass: 441.0393

(C)

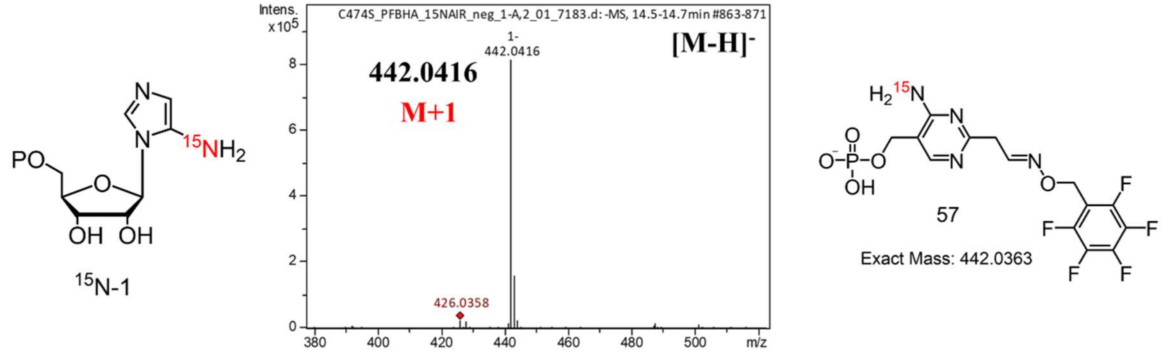

C474S_PFBHA_15NAIR_neg_1-A,2_01_7183.d: -MS, 14.5-14.7min #863-871
Intens. x10^5
442.0416
M+1
[M-H]^-
1- 442.0416
426.0358
m/z
15N-1
57
Exact Mass: 442.0363

(D)

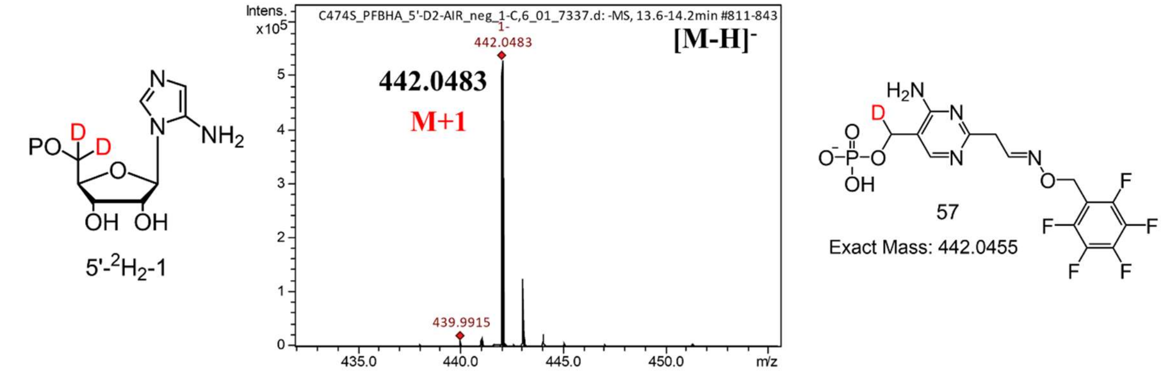

C474S_PFBHA_5'-D2-AIR_neg_1-C,6_01_7337.d: -MS, 13.6-14.2min #811-843
Intens. x10^5
442.0483
M+1
[M-H]^-
1- 442.0483
439.9915
m/z
5'-2H2-1
57
Exact Mass: 442.0455

(E)

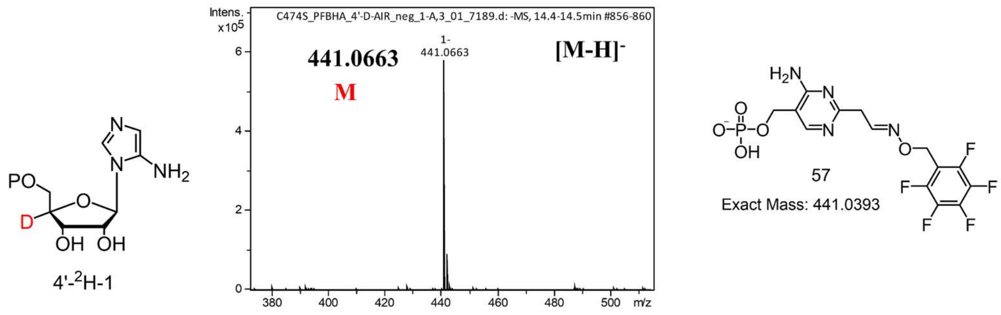

C474S_PFBHA_4'-D-AIR_neg_1-A,3_01_7189.d: -MS, 14.4-14.5min #856-860
441.0663
M
[M-H]-
4'-2H-1
57
Exact Mass: 441.0393

(F)

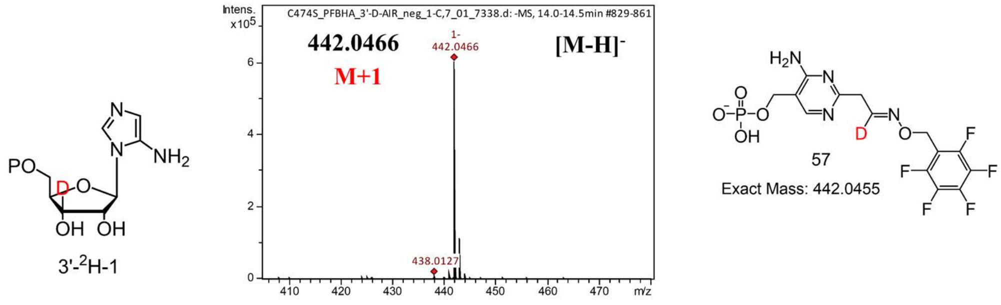

C474S_PFBHA_3'-D-AIR_neg_1-C,7_01_7338.d: -MS, 14.0-14.5min #829-861
442.0466
M+1
[M-H]-
438.0127
3'-2H-1
57
Exact Mass: 442.0455

(G)

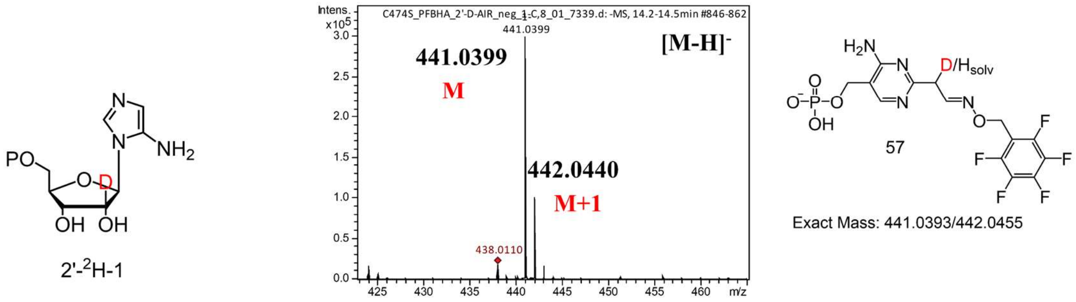

C474S_PFBHA_2'-D-AIR_neg_1-C,8_01_7339.d: -MS, 14.2-14.5min #846-862
441.0399
M
442.0440
M+1
[M-H]-
438.0110
2'-2H-1
D/Hsolv
57
Exact Mass: 441.0393/442.0455

(H)

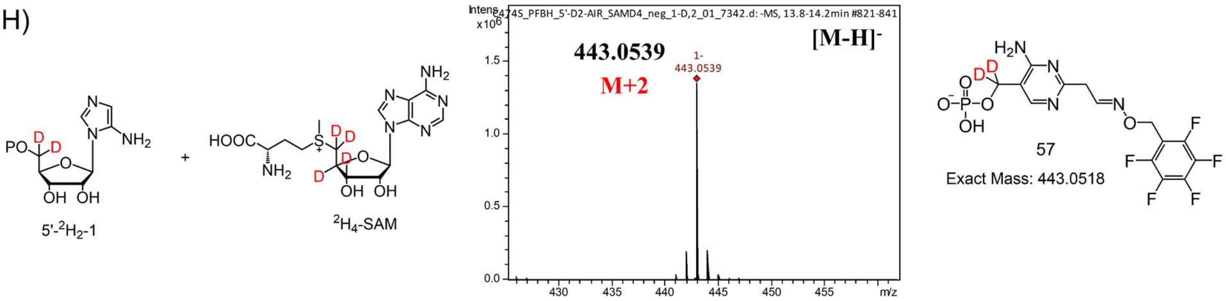

5'-2H2-1
2H4-SAM
C474S_PFBH_5'-D2-AIR_SAMD4_neg_1-D,2_01_7342.d: -MS, 13.8-14.2min #821-841
443.0539
M+2
[M-H]-
57
Exact Mass: 443.0518

(I)

50% ^2H_2O

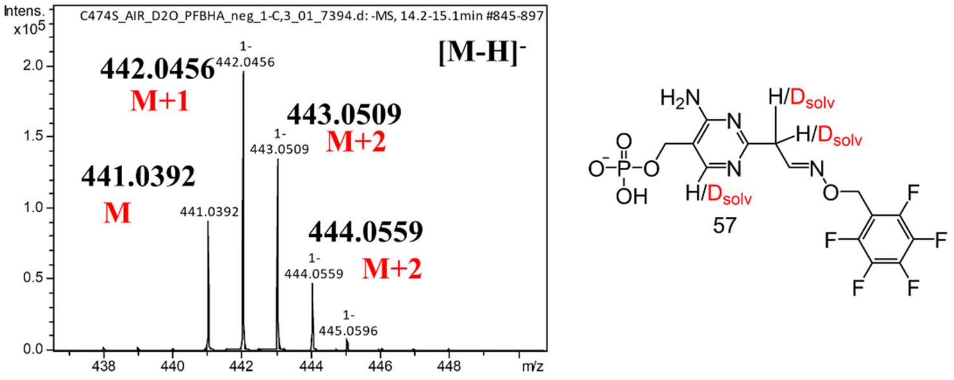

(J)

50% H_2O^{18}

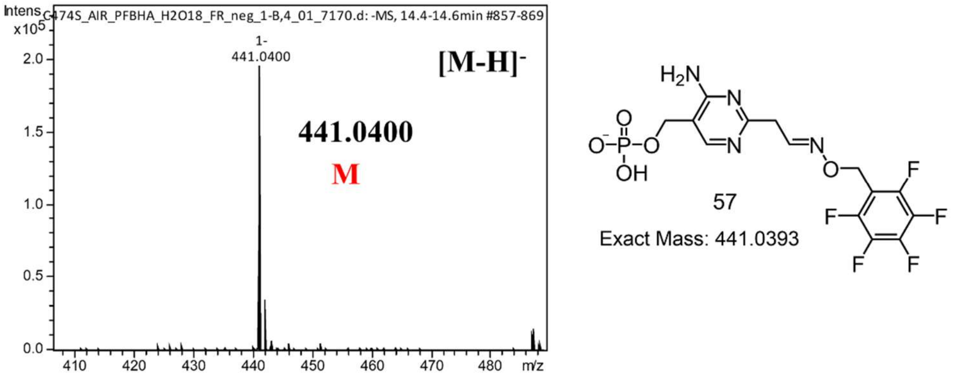

(K)

Isotopologue of **1**	Mass of **57**
**1**	**441 (M)**
1’,2’,3’,4’,5’-^{13}C-**1**	**445 (M+4)**
1’-^{13}C-**1**	**441 (M)**
^{15}N-**1**	**442 (M+1)**
5’-$^2H_2$-**1**	**442 (M+1)**
4’-^{2}H-**1**	**441 (M)**
3’-^{2}H-**1**	**442 (M+1)**
2’-^{2}H-**1**	**441:442 (M+1)**
5’-$^2H_2$-**1** + $^2H_4$-SAM	**443 (M+2)**
50% ^2H_2O	**441:442:443:444 (M+3)**
50% H_2O^{18}	**441 (M)**

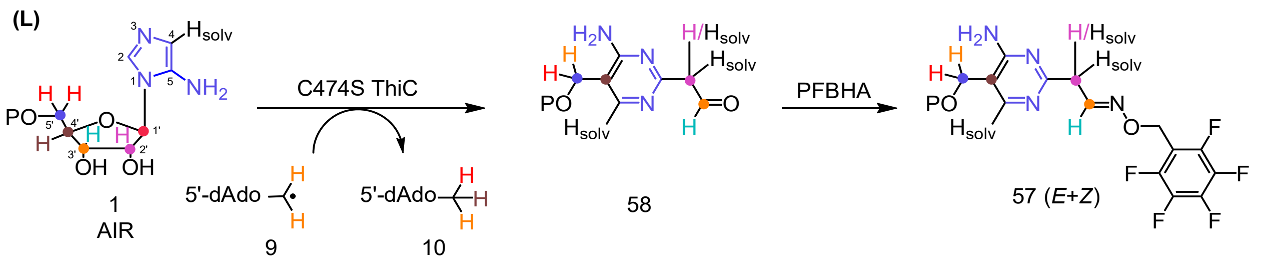

Figure S37. MS analysis of **57** formed upon using AIR (**1**) isotopologues and labeled solvent in the *Cc*ThiC (C474S) catalyzed reaction. (A) 1',2',3',4',5'-$^{13}C_5$-**1**. (B) 1'-^{13}C-**1**. (C) 5-^{15}N-**1**. (D) 5'-$^{2}H_2$-**1**. (E) 4'-^{2}H-**1**. (F) 3'-^{2}H-**1**. (G) 2'-^{2}H-**1**. The 2'-^{2}H is retained at the C2' carbon in **58**. However, the pKa of 2'-^{2}H decreases because of the α carbonyl group, and it exchanges with solvent. (H) 5'-$^{2}H_2$-**1** & $^{2}H_4$-SAM. (I) 50% $^{2}H_2O$. (J) 50% H_2O^{18}. (K) Summary of LC-MS analysis of label transfer from isotopologues of AIR to **57**. (L) The *Cc*ThiC (C474S) catalyzed reaction with the fate of all atoms shown in color.

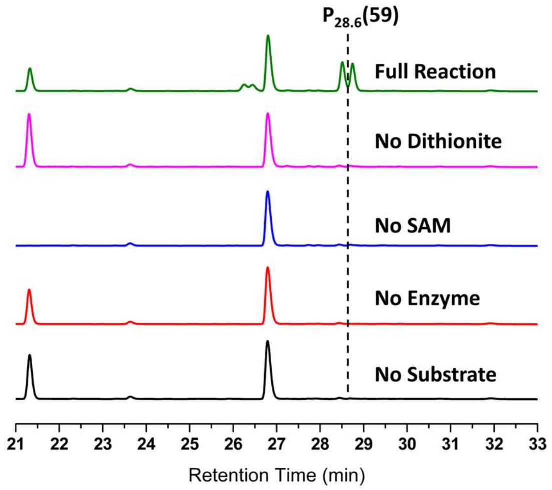

Figure S38. HPLC analysis of the *Cc*ThiC (C474S) catalyzed the reaction of AIR after CIP treatment and the addition of PFBHA, showing a new product $P_{28.6}$ (**59**) in the full reaction only. **59** is the dephosphorylated form of **57**.

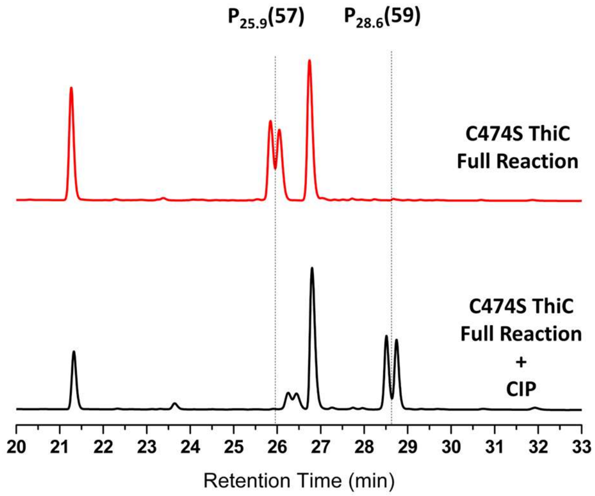

Figure S39. HPLC analysis of the *Cc*ThiC (C474S) catalyzed reaction of AIR after the addition of PFBHA shows the formation of $P_{25.9}$ (**57**) (Red Trace), and HPLC analysis of the *Cc*ThiC (C474S) catalyzed reaction of AIR after CIP treatment and the addition of PFBHA shows the formation of $P_{28.6}$ (**59**) (Black Trace). The comparison shows that **57** is converted to **59** upon CIP treatment.

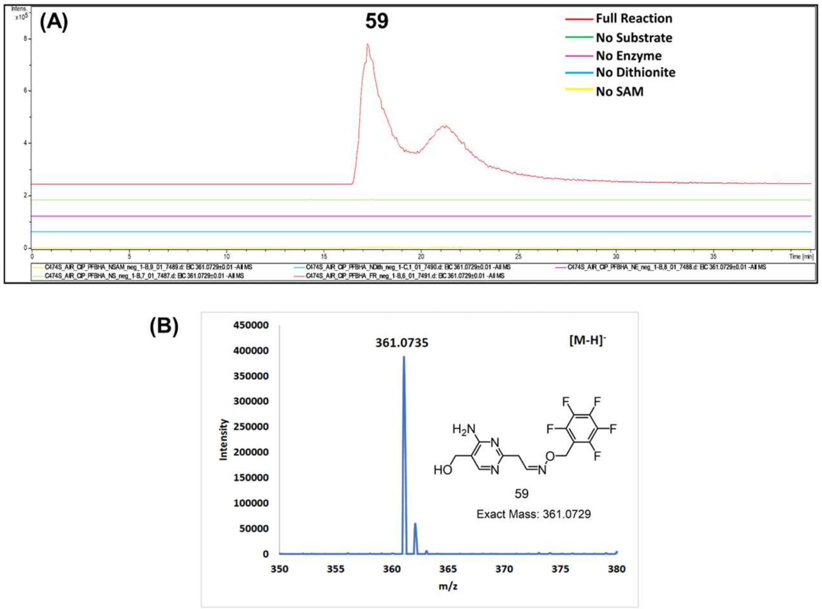

Figure S40. LC-MS of **59** in the *Cc*ThiC (C474S) catalyzed reaction (negative ion mode). (A) EIC of **59**. (B) $[M-H]^-$ for **59**.

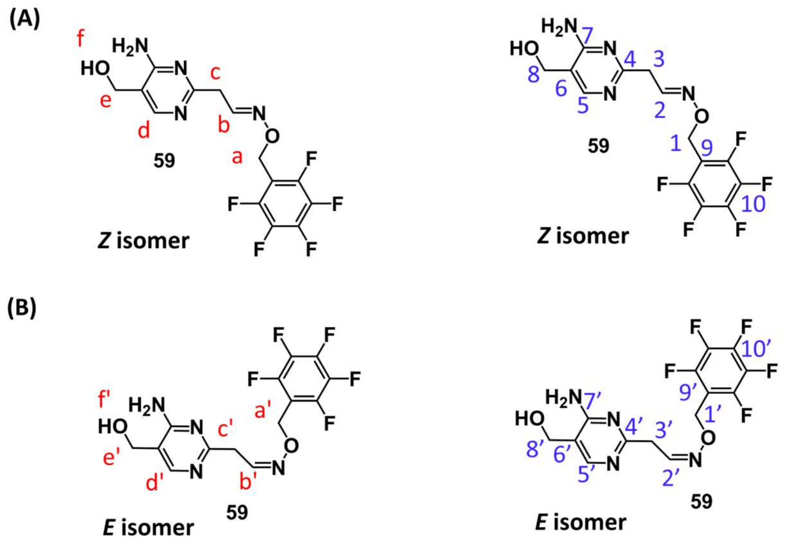

Figure S41. Structures of different isomers of **59** (A) *Z* isomer of **59** with hydrogens annotated from a-f and carbons annotated from 1-10. (B) *E* isomer of **59** with hydrogens annotated from a' - f' and carbon annotated from 1' -10'.

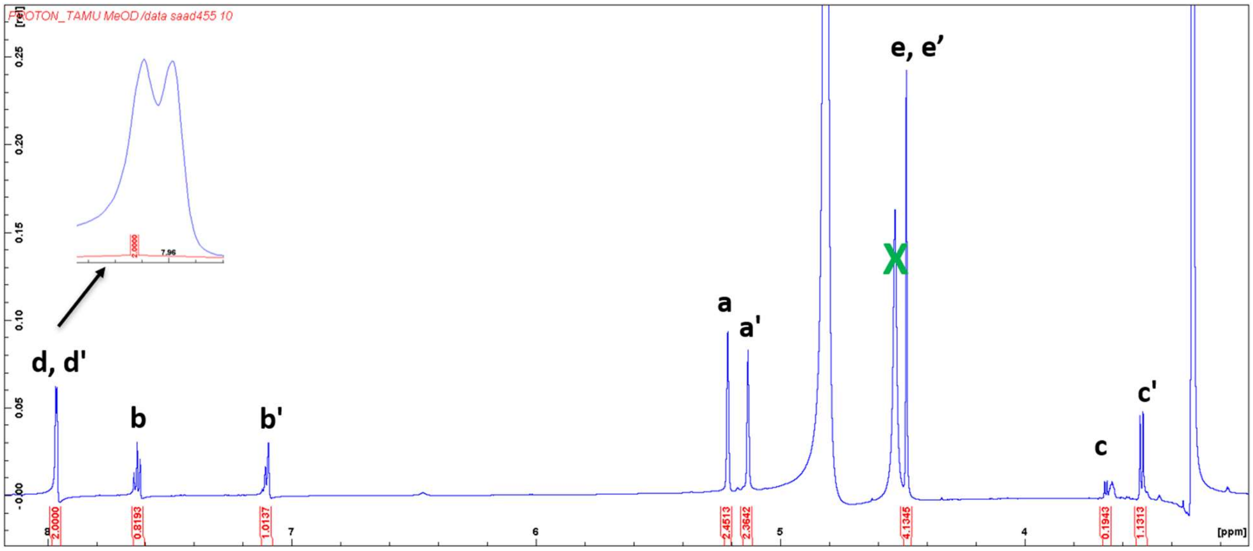

Figure S42. ^{1}H-NMR of **59**.

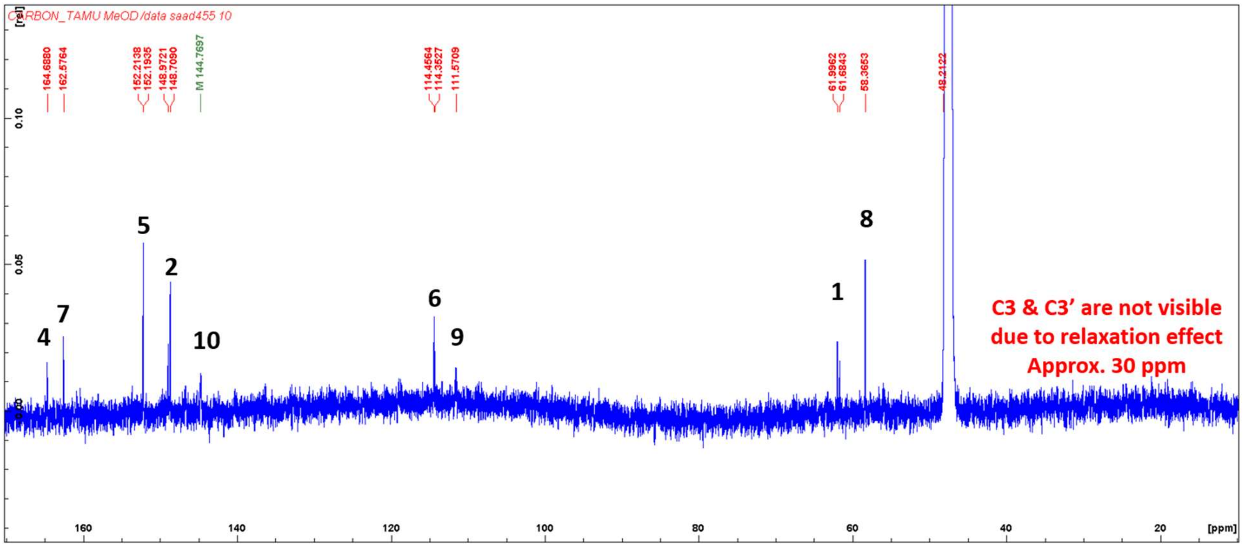

Figure S43. ^{13}C-NMR of **59**.

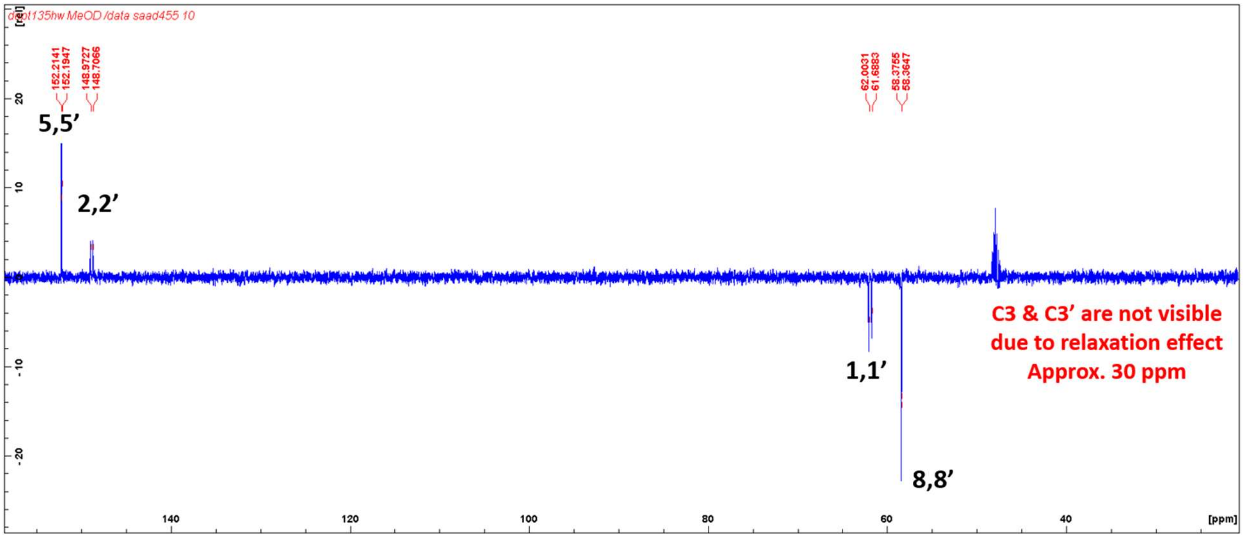

Figure S44. DEPT-135 NMR of **59**.

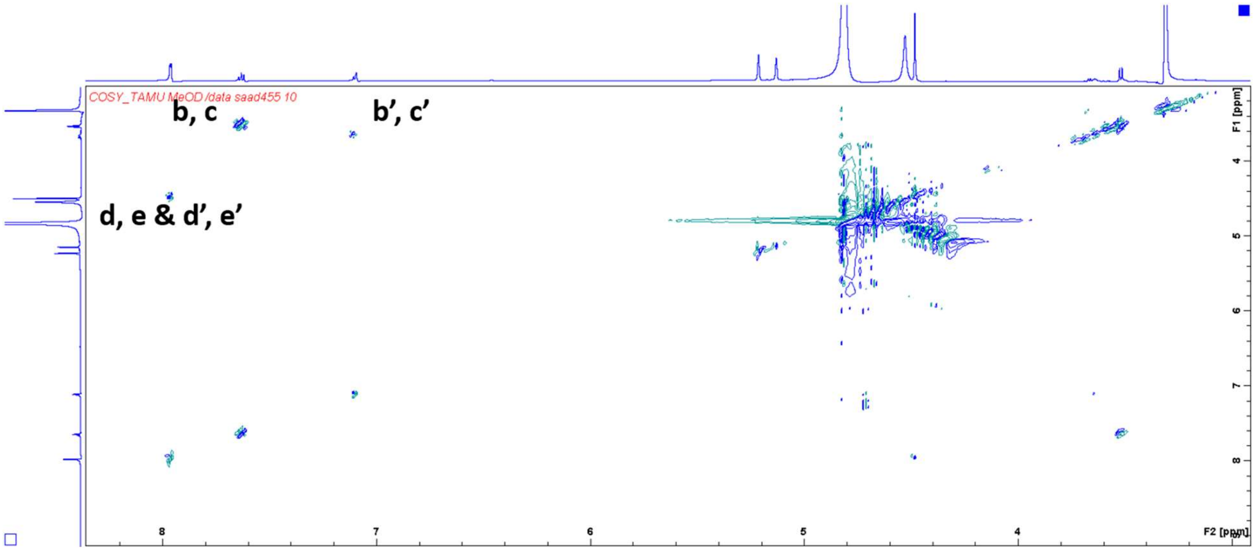

Figure S45. ^{1}H-^{1}H COSY NMR of 59.

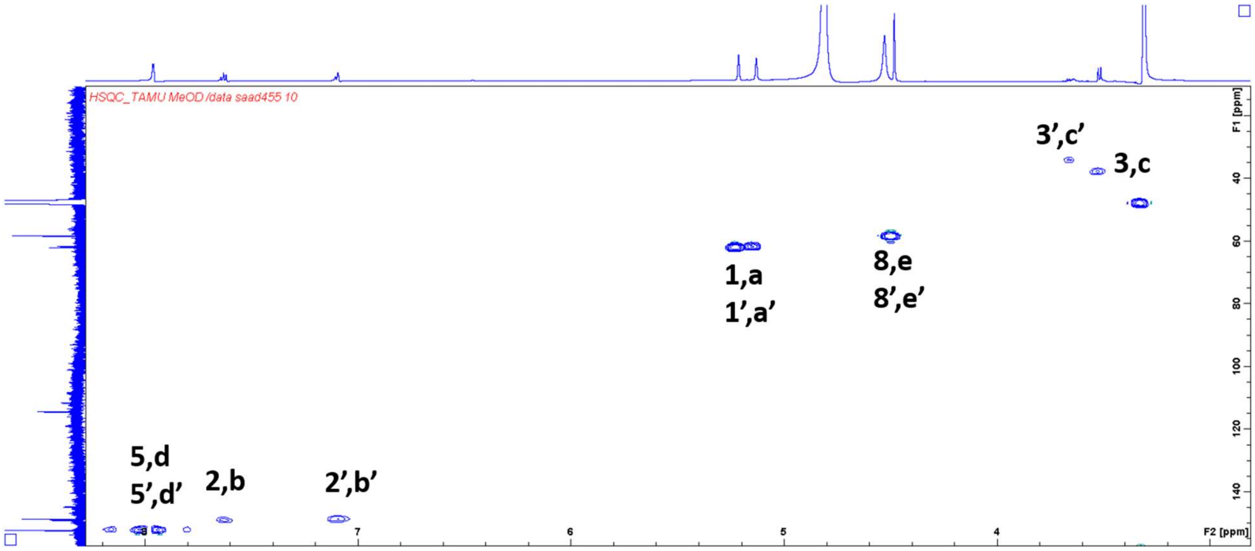

Figure S46. ^{1}H-^{13}C HSQC NMR of **59**.

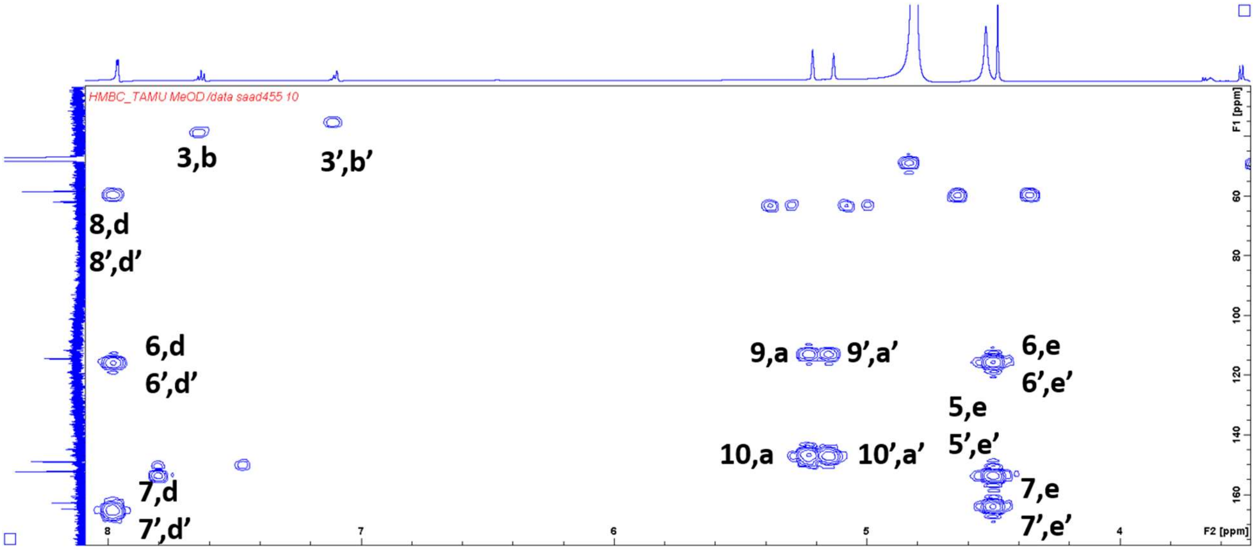

Figure S47. $^{1}H-^{13}C$ HMBC NMR of 59.

Quantitation of shunt product 57 in the *Cc*ThiC C474S reaction

To quantify **57**, a commercial standard of formaldehyde PFBHA oxime **88** and HMP **87** was used to derive calibration curves and calculate their respective extinction coefficient. The extinction coefficient of **87** and **88** were added to get an approximate extinction coefficient of **57**. (Figure S47B & D). Based on the calculated extinction coefficient of **57**, the amount of **57** formed in the *Cc*ThiC (C474S) catalyzed reaction was approximately 80 µM. The ratio of **57**:5'-dA is approximately 1:6. Reaction conditions: Enzyme=300 µM, AIR= 410 µM, SAM= 1mM, sodium dithionite = 2mM.

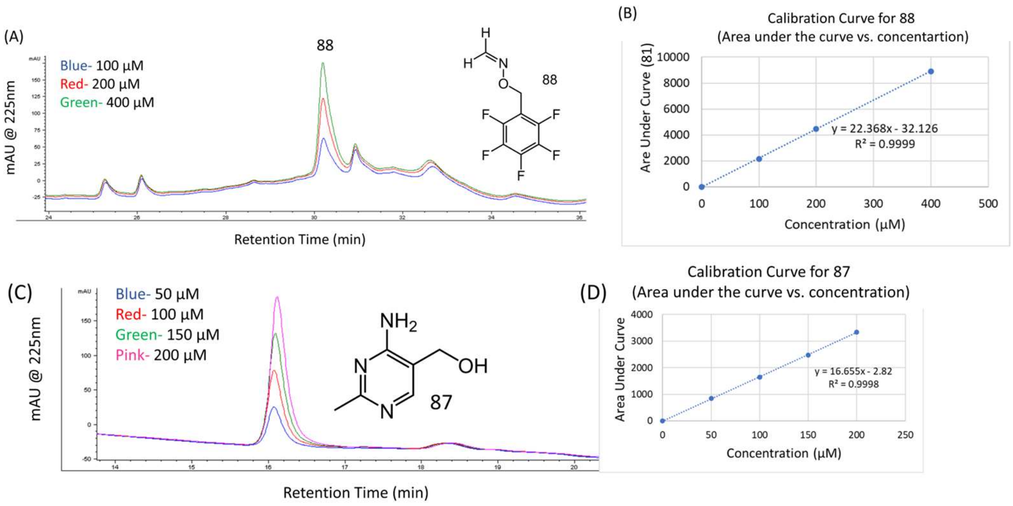

Figure S48. Quantitation of shunt product **57** in the *Cc*ThiC (C474S) catalyzed reaction of AIR. (A) HPLC traces for different concentrations of formaldehyde PFBHA oxime **88**. (B) Calibration curve for **88**. (C) HPLC traces for different concentrations of HMP **87**. (B) Calibration curve for **87**.

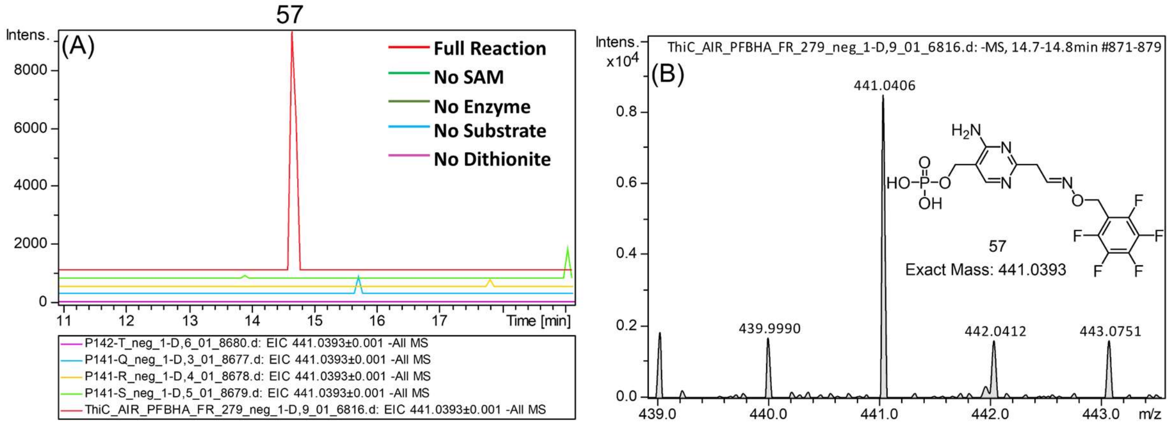

Figure S49. LC-MS of **57** formed in the wt *Cc*ThiC-catalyzed reaction of AIR (negative ion mode) (A) EIC of **57**. (B) $[M\text{-}H]^-$ for **57**.

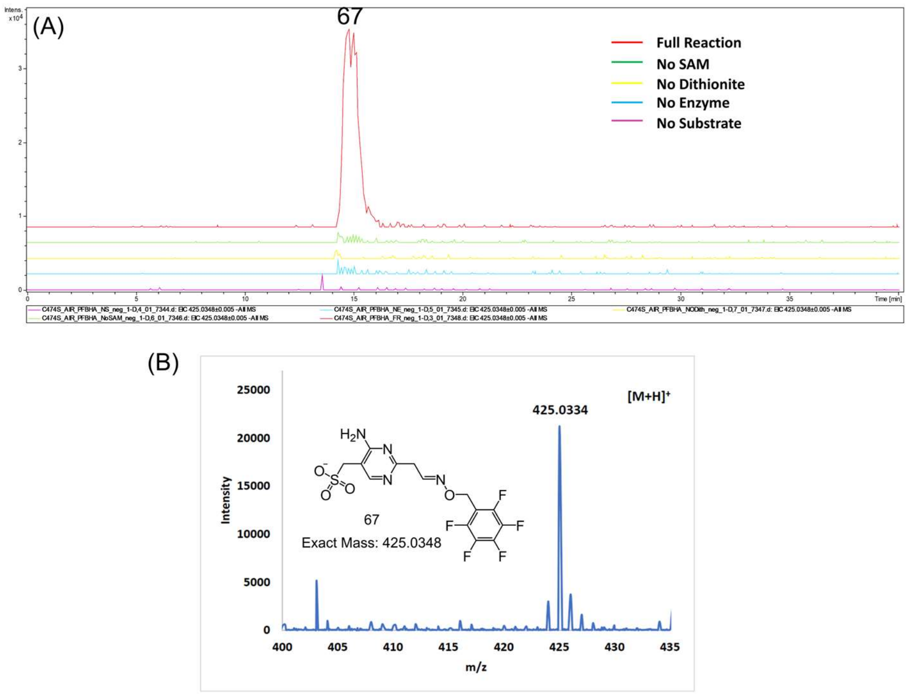

Figure S50. LC-MS of **67** in the *Cc*ThiC (C474S) catalyzed reaction (negative ion mode). (A) EIC of **67**. (B) $[M-H]^-$ for **67**.

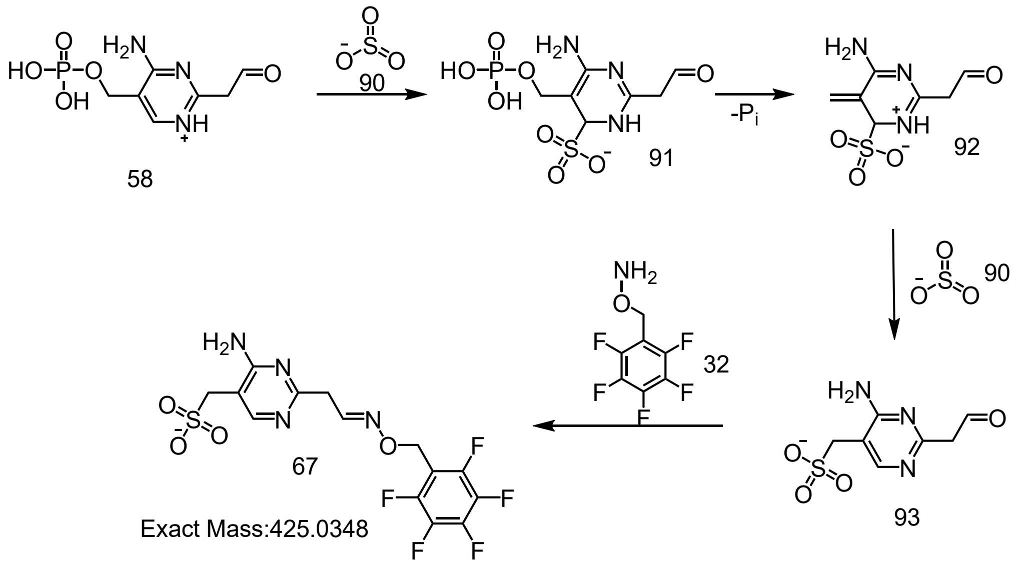

Figure S51. Proposed mechanism for the formation of **67** in the *Cc*ThiC (C474S) catalyzed reaction.

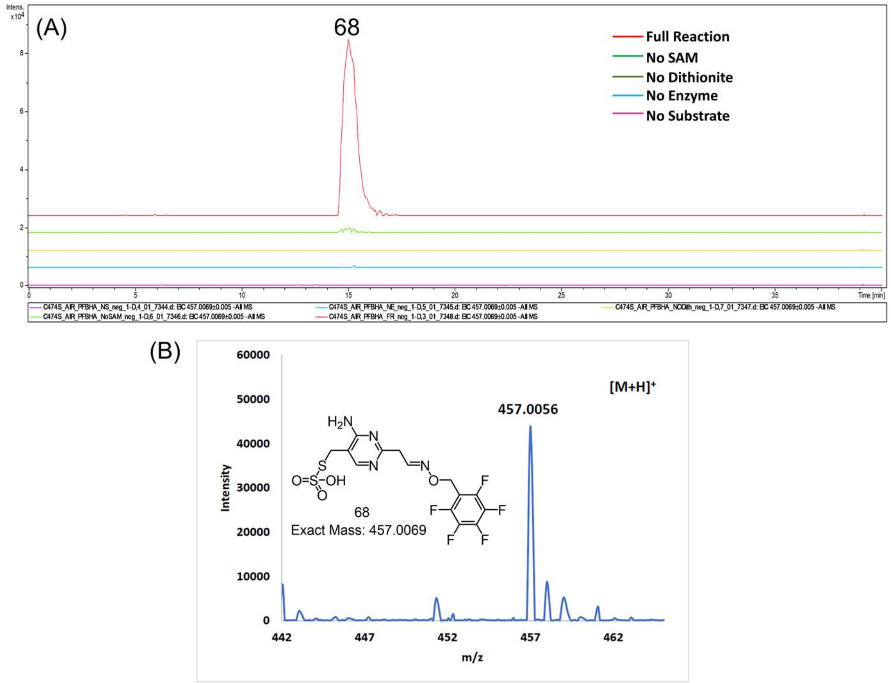

Figure S52. LC-MS of **68** formed in the *Cc*ThiC (C474S) catalyzed (negative ion mode). (A) EIC of **68**. (B) [M-H]$^-$ for **68**.

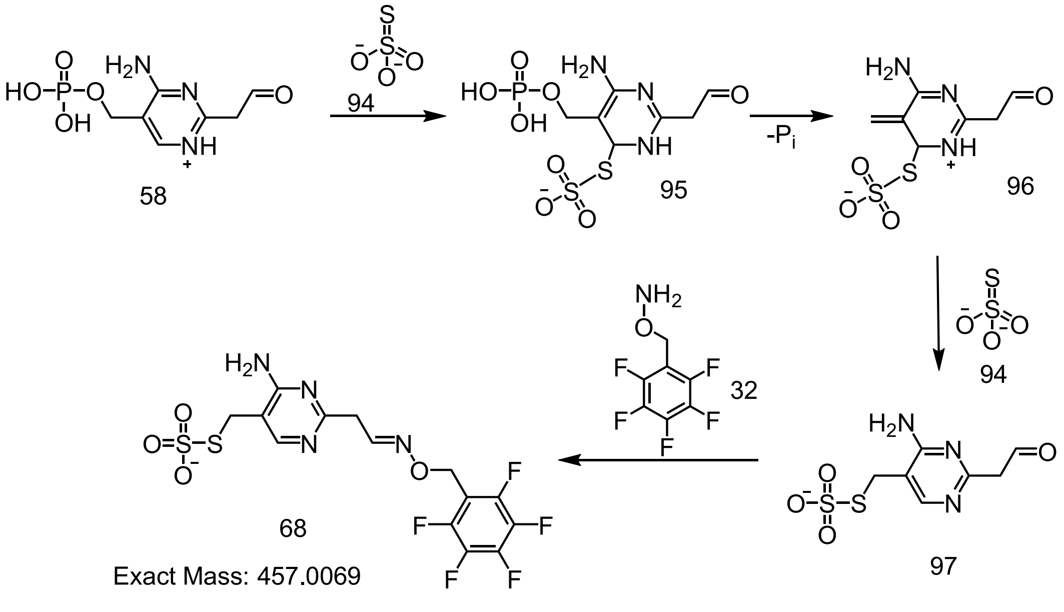

Figure S53. Proposed mechanism for the formation of **68** in the *Cc*ThiC (C474S) catalyzed reaction.

(A)

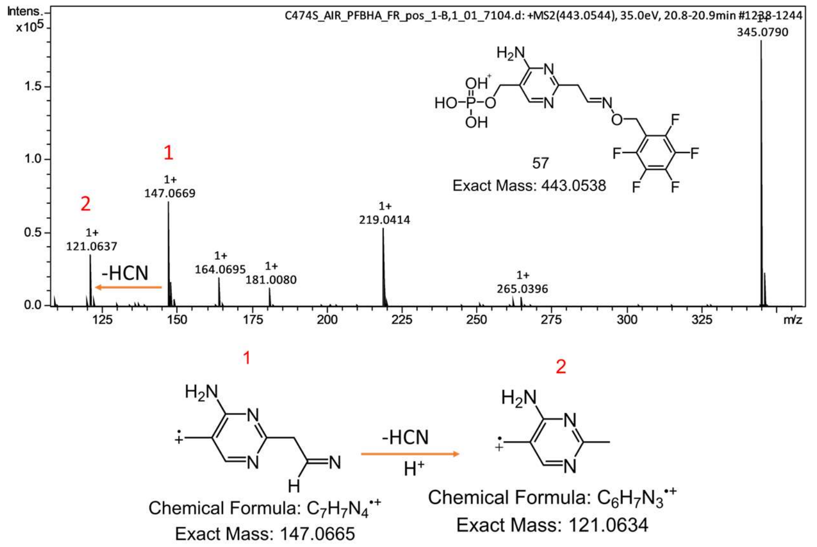

(B)

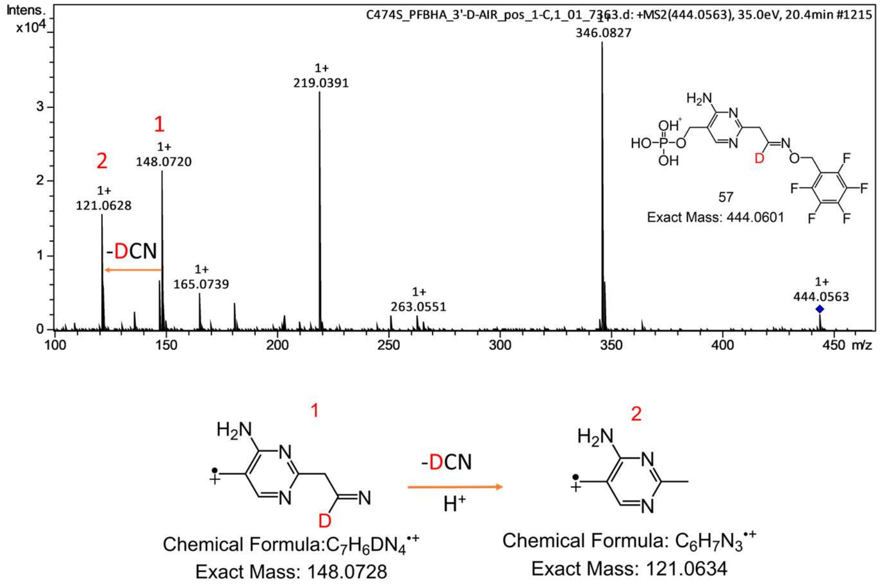

Figure S54. (A) Positive mode MS-MS analysis of **57** formed in the *Cc*ThiC (C474S) catalyzed reaction of AIR. (B) Positive mode MS-MS analysis of **57** formed in the *Cc*ThiC C474S reaction with 3'-^{2}H-AIR.

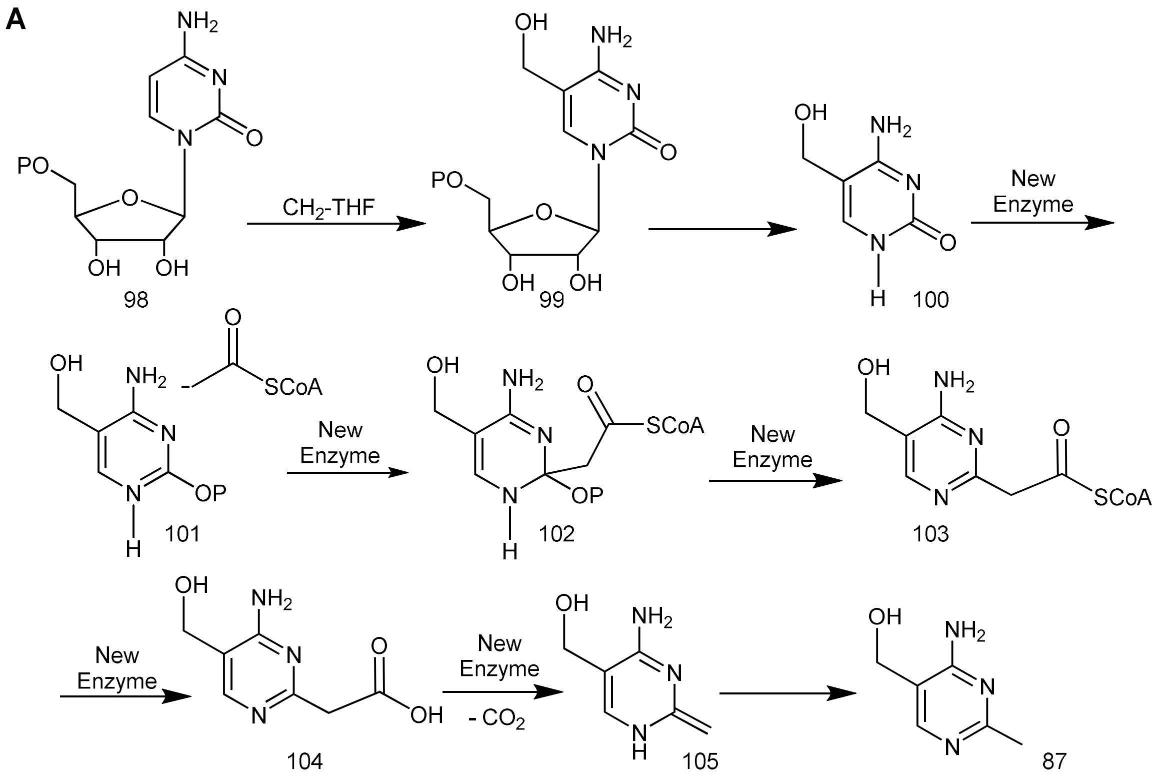

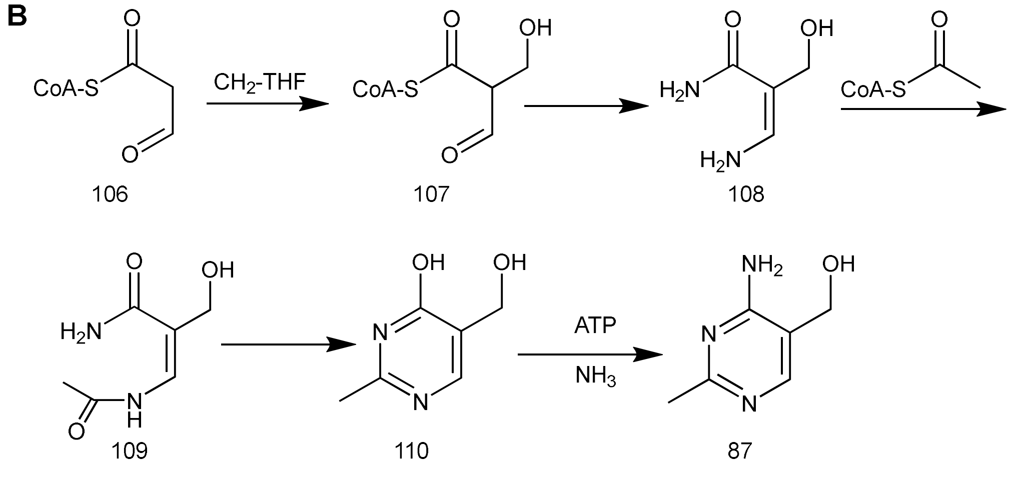

Figure S55 . Proposed alternative routes to HMP. A) Route based on bacimethrin biosynthesis. B) Route based on the canonical pyrimidine biosynthesis. Extensive genome sequence analysis of thiamin biosynthetic gene clusters suggests that the ThiC and THI5 catalyzed reactions are the only routes to the thiamin pyrimidine.

References

1. Raschke, M.; Burkle, L.; Muller, N.; Nunes-Nesi, A.; Fernie, A. R.; Arigoni, D.; Amrhein, N.; Fitzpatrick, T. B., Vitamin B1 biosynthesis in plants requires the essential iron sulfur cluster protein, THIC. *Proc Natl Acad Sci U S A* **2007,** *104* (49), 19637-42.

2. Chatterjee, A.; Li, Y.; Zhang, Y.; Grove, T. L.; Lee, M.; Krebs, C.; Booker, S. J.; Begley, T. P.; Ealick, S. E., Reconstitution of ThiC in thiamine pyrimidine biosynthesis expands the radical SAM superfamily. *Nat Chem Biol* **2008,** *4* (12), 758-65.

3. Hänzelmann, P.; Hernández, H. L.; Menzel, C.; García-Serres, R.; Huynh, B. H.; Johnson, M. K.; Mendel, R. R.; Schindelin, H., Characterization of MOCS1A, an Oxygen-sensitive Iron-Sulfur Protein Involved in Human Molybdenum Cofactor Biosynthesis *Journal of Biological Chemistry* **2004,** *279* (33), 34721-34732.

4. Zhang, Y.; Dougherty, M.; Downs, D. M.; Ealick, S. E., Crystal structure of an aminoimidazole riboside kinase from Salmonella enterica: implications for the evolution of the ribokinase superfamily. *Structure* **2004,** *12* (10), 1809-21.

5. Lawhorn, B. G.; Mehl, R. A.; Begley, T. P., Biosynthesis of the thiamin pyrimidine: the reconstitution of a remarkable rearrangement reaction. *Org Biomol Chem* **2004,** *2* (17), 2538-46.

6. Chatterjee, A.; Hazra, A. B.; Abdelwahed, S.; Hilmey, D. G.; Begley, T. P., A "radical dance" in thiamin biosynthesis: mechanistic analysis of the bacterial hydroxymethylpyrimidine phosphate synthase. *Angew Chem Int Ed Engl* **2010,** *49* (46), 8653-6.

7. Mehta, A. P.; Abdelwahed, S. H.; Fenwick, M. K.; Hazra, A. B.; Taga, M. E.; Zhang, Y.; Ealick, S. E.; Begley, T. P., Anaerobic 5-Hydroxybenzimidazole Formation from Aminoimidazole Ribotide: An Unanticipated Intersection of Thiamin and Vitamin B12 Biosynthesis. *J Am Chem Soc* **2015,** *137* (33), 10444-7.

8. Fenwick, M. K.; Mehta, A. P.; Zhang, Y.; Abdelwahed, S. H.; Begley, T. P.; Ealick, S. E., Non-canonical active site architecture of the radical SAM thiamin pyrimidine synthase. *Nat Commun* **2015,** *6*, 6480.

9. Harusawa, S.; Murai, Y.; Moriyama, H.; Imazu, T.; Ohishi, H.; Yoneda, R.; Kurihara, T., Efficient and beta-Stereoselective Synthesis of 4(5)-(beta-D-Ribofuranosyl)- and 4(5)-(2-Deoxyribofuranosyl)imidazoles(1). *J Org Chem* **1996,** *61* (13), 4405-4411.
