# Supplementary material for: Phosphomethylpyrimidine Synthase (ThiC): Trapping of Five Intermediates Provides Mechanistic Insights on a Complex Radical Cascade Reaction in Thiamin Biosynthesis
Source: ACS Cent Sci. 2024 May 13;10(5):988–1000. doi: 10.1021/acscentsci.4c00125 (PMC11117688; doi:10.1021/acscentsci.4c00125)
Supplement: Supplementary file 2 — oc4c00125_si_002.pdf [file oc4c00125_si_002.pdf]

oc-2024-00125x.R1

Name: Peer Review Information for "Phosphomethylpyrimidine Synthase (ThiC): Trapping of Five Intermediates Provides Mechanistic Insights on a Complex Radical Cascade Reaction in Thiamin Biosynthesis."

First of Reviewer Comments

Reviewer: 1

Comments to the Author

The authors investigated the reaction mechanism of ThiC, one of the most complex radical reactions. They detected intermediates and shunt products using wild-type and variants with substituted active site amino acids. Deuterated substrates and substrate analogs were also employed to gain further mechanistic insights. The proposed mechanism is consistent with the observations. The manuscript is well-written. The presented information and mechanisms would be highly interesting to the readership in the community of mechanistic enzymology. Therefore, the content of this manuscript would be worthy of publication after addressing the following comments.

1. To compare the 30 production by the wild type and the D383A mutant, can the authors determine the concentration of 30 produced? The authors mentioned that the D383A mutant did not produce other detectable side product. I wonder if the consumed amount of AIR is consistent with the amount of 30 produced.

2. The reaction of CcThiC generated 41 after derivatization. Can the authors detect 16 as a byproduct? Detection of 16 would further support the proposal that 41 is a consequence of reduction of 33, not via oxidation.

Reviewer: 2

Comments to the Author

In this study the authors investigate the remarkable reaction catalyzed by ThiC during thioamin biosynthesis. This reaction is stunningly complex and while the Begley group has done a

phenomenal amount of work to establish its mechanism, many uncertainties still remained. In this work, the authors look at a series of mutant enzymes of active site residues using a new method in which they trap intermediates that have no chromophore to make them visible and allow their characterization. They also use a substrate analog in similar manner. The analysis is first rate and very complete with full characterization by NMR and MS and using isotopologues to not only confirm the assigned structures but also provide insights in the origin of the atoms. The net result of this beautiful study is a revision of the previously proposed mechanism with respect to the middle and late steps of the process. Importantly, the revision is not a small nuance but a fundamental change from the previously suggested mechanism, while still being consistent with all the previous studies.

The fact that we still do not understand the details of the formation of a critical cofactor for life (thiamin) is a testament of the complexity of the ThiC reaction. Despite the complexity of the reaction and the current study, the authors do an excellent job making the findings and conclusions accessible to the reader. I very strongly support publication in ACS Chem Sci. These types of studies are not very common anymore but they are absolutely necessary to better understand how enzymes can catalyze highly complex transformations. I also found the proposed potential evolutionary basis for the structure of thiamin and its biosynthesis that does not utilize “standard” nucleotide enzymology intriguing.

I have some suggestions to improve the presentation and some minor suggestions on wording.

Table 1, entry on compound 54. I am not sure I agree that one can unambiguously say that the outcome with this substrate analog demonstrates that C4’-O bond cleavage is the first reaction of the AIR radical. I would suggest replacing “demonstrates” with “supports”. Same for the sentence on p. 8, line 7 that the outcome with 43 “establishes” that C4’-O bond cleavage is the first reaction of AIR radical.

The authors do not mention in the paper whether all the residues they mutated are completely conserved. I suspect they are (and it is possible the authors have previously commented on this) and it would be good to point this out, either in the introduction where the X-ray structure is discussed, or in the individual sections in which the mutants are introduced.

P17 is first mentioned on p. 4 and P24 on page 6 but it was not until the explanation of the nomenclature for P25.9 was explained on page 8 that I understood that P17 means the compound elutes at 17 min and P24 at 24 min. I suggest to introduce that nomenclature on p 4 at first use because it was puzzling to me until page 8.

It may be a matter of taste, but in many of the radical reactions three electrons are “moving” whereas the authors draw only two single headed arrows in the various mechanistic figures with the positions of some of the arrows a bit ambiguous. I realize that the whole concept of arrows is flawed but I think the current drawings can be confusing.

Regarding the conversion of 28 to 60, it may be useful to look at a study on the deprotonation of an enoxy radical which is discussed in Smith, D. M.; Buckel, W.; Zipse, H. Angew. Chem., Int. Ed. 2003, 752 42, 1867. I did not read the paper again but it may have information that is relevant (i.e. in the reverse direction, deprotonation of 60).

Figure S3 and elsewhere. In panel B the MSMS spectrum is shown and dashed lines indicates fragmentation but the fragments are not linked to specific masses/peaks. Given that the fragmentation is not very efficient it is not clear which peaks are which fragments. Annotation would be helpful, here and in other MSMS spectra.

Figure S9. The authors mention they detect AIR as compound 86 and HMP-P as compound 87. I think these are dephosphorylated compounds but the structure of 86 is never provided (or I could not find it) and the structure of 87 does not show up until Figure S47. I suggest to draw them in Figure S9 in the same way that the authors provide the structure of compound 88 in Figure S12. This is important given how often 86 and 87 is mentioned in the SI figures.

Figure S29. The WT reaction seems to produce one peak at ~15 min that is neither in the no-enzyme control nor in the C475S reaction. Is this peak reproducibly observed? What is its mass?

Author's Response to Peer Review Comments:

Response to the Reviewers’ Comments

Note: The figure numbers in the SI have been updated and the main paper now reflects the new SI figure numbers.

Formatting Needs:

MS Files: Synopsis is missing.

A synopsis has now been added at the end of the paper.

Reviewer: 1

Recommendation: Publish in ACS Central Science after minor revisions noted.

Comments:
The authors investigated the reaction mechanism of ThiC, one of the most complex radical reactions. They detected intermediates and shunt products using wild-type and variants with substituted active site amino acids. Deuterated substrates and substrate analogs were also employed to gain further mechanistic insights. The proposed mechanism is consistent with the observations. The manuscript is well-written. The presented information and mechanisms would be highly interesting to the readership in the community of mechanistic enzymology. Therefore, the content of this manuscript would be worthy of publication after addressing the following comments.

We thank the reviewer for appreciating our work.

1. To compare the 30 production by the wild type and the D383A mutant, can the authors determine the concentration of 30 produced? The authors mentioned that the D383A mutant did not produce other detectable side product. I wonder if the consumed amount of AIR is consistent with the amount of 30 produced.

Supporting Information, Page S20: In Figure S13, we reported the quantitation of **30** in the *At*ThiC (D383A)-catalyzed reaction.

The *text states: "Reaction conditions: Enzyme=300 μM, AIR= 410 μM, SAM= 1mM, Ti (III) citrate= 6mM. The amount of AIR consumed was 250 μM and the amount of 5'-dA formed was 280 μM"* has been added above Figure S13 to make it clearer.

The amount of **30** formed in the *At*ThiC (D383A) reaction is approximately 32 μM, which is clearly less than the amount of AIR consumed (250 μM). Therefore, it is likely that *At*ThiC (D383A) produces other shunt products that are not detectable under our reaction conditions. The same can also be said about the wild-type ThiC. Even though we detect multiple shunt products in the wild-type reaction, additional ones likely remain undetectable. Further studies are needed to identify such shunt products.

2. The reaction of CcThiC generated 41 after derivatization. Can the authors detect 16 as a byproduct? Detection of 16 would further support the proposal that 41 is a consequence of reduction of 33, not via oxidation.

Page 6, Studies on *Cc*ThiC (E413Q), Figure 6E: Our attempts to detect **16** in the *Cc*ThiC (E413Q) reaction were unsuccessful, likely because of the high reactivity of **16.** (Reference #26)

Figure 6E: It is equally likely that **41** is formed after the oxidation of **33**. We did consider the possibility of oxidation of **33,** however, **33** being a radical cation is likely to have a higher reduction potential, and the presence of a strong reducing agent (titanium (III) citrate) in the reaction mixture will favor the reduction of **33**. In the absence of any data for the detection of **16**, we proposed the reduction of **33** for the reason mentioned above.

Additional Questions:

Quality of experimental data, technical rigor: Top 5%

Significance to chemistry researchers in this and related fields: Top 5%

Broad interest to other researchers: Top 5%

Novelty: Top 5%

Is this research study suitable for media coverage or a First Reactions (a News & Views piece in the journal)?: Yes

Reviewer: 2

Recommendation: Publish in ACS Central Science after minor revisions noted.

Comments:
In this study the authors investigate the remarkable reaction catalyzed by ThiC during thioamin biosynthesis. This reaction is stunningly complex and while the Begley group has done a phenomenal amount of work to establish its mechanism, many uncertainties still remained. In this work, the authors look at a series of mutant enzymes of active site residues using a new method in which they trap intermediates that have no chromophore to make them visible and allow their characterization. They also use a substrate analog in similar manner. The analysis is first rate and very complete with full characterization by NMR and MS and using isotopologues to not only confirm the assigned structures but also provide insights in the origin of the atoms. The net result of

this beautiful study is a revision of the previously proposed mechanism with respect to the middle and late steps of the process. Importantly, the revision is not a small nuance but a fundamental change from the previously suggested mechanism, while still being consistent with all the previous studies.

The fact that we still do not understand the details of the formation of a critical cofactor for life (thiamin) is a testament of the complexity of the ThiC reaction. Despite the complexity of the reaction and the current study, the authors do an excellent job making the findings and conclusions accessible to the reader. I very strongly support publication in ACS Chem Sci. These types of studies are not very common anymore but they are absolutely necessary to better understand how enzymes can catalyze highly complex transformations. I also found the proposed potential evolutionary basis for the structure of thiamin and its biosynthesis that does not utilize "standard" nucleotide enzymology intriguing. I have some suggestions to improve the presentation and some minor suggestions on wording.

We are grateful to the reviewer for such encouraging words about our work and for providing valuable suggestions.

Table 1, entry on compound 54. I am not sure I agree that one can unambiguously say that the outcome with this substrate analog demonstrates that C4'-O bond cleavage is the first reaction of the AIR radical. I would suggest replacing "demonstrates" with "supports". Same for the sentence on p. 8, line 7 that the outcome with 43 "establishes" that C4'-O bond cleavage is the first reaction of AIR radical.

Agreed.

Page 8, Paragraph 1: "Establishes" is replaced with "supports".

Page 11, Table 1 (Row 4, Column 2): "Demonstrates" is replaced with "supports".

The authors do not mention in the paper whether all the residues they mutated are completely conserved. I suspect they are (and it is possible the authors have previously commented on this) and it would be good to point this out, either in the introduction where the X-ray structure is discussed, or in the individual sections in which the mutants are introduced.

Page 6, Paragraph 1: The text “*All the residues mutated in this study are highly conserved across ThiC orthologs*” has now been added.

P17 is first mentioned on p. 4 and P24 on page 6 but it was not until the explanation of the nomenclature for P25.9 was explained on page 8 that I understood that P17 means the compound elutes at 17 min and P24 at 24 min. I suggest to introduce that nomenclature on p 4 at first use because it was puzzling to me until page 8.

We have now explained this nomenclature on page 4, paragraph 3.

It may be a matter of taste, but in many of the radical reactions three electrons are “moving” whereas the authors draw only two single headed arrows in the various mechanistic figures with the positions of some of the arrows a bit ambiguous. I realize that the whole concept of arrows is flawed but I think the current drawings can be confusing.

To be uniform across all the schemes, we have removed all the arrows.

Regarding the conversion of 28 to 60, it may be useful to look at a study on the deprotonation of an enoxy radical which is discussed in Smith, D. M.; Buckel, W.; Zipse, H. Angew. Chem., Int. Ed. 2003, 752 42, 1867. I did not read the paper again but it may have information that is relevant (i.e. in the reverse direction, deprotonation of 60).

Page 10, Figure 10: The tautomerization of **28** to **60** is proposed based on our preliminary mechanistic proposal (Figure 3). Based on the data presented in this study, **28** is no longer an intermediate in our current mechanistic proposal (Page 13, Figure 11).

However, the reference is an important example of large acidity enhancements of protons beta to radicals. It has now been cited (ref #51) for the conversion of **63** to **64** in Figure 11.

Figure S3 and elsewhere. In panel B the MSMS spectrum is shown and dashed lines indicates fragmentation but the fragments are not linked to specific masses/peaks. Given that the fragmentation is not very efficient it is not clear which peaks are which fragments. Annotation would be helpful, here and in other MSMS spectra.

All the MS-MS spectra are now annotated (Supporting Information: Figures S4, S17, S29, S35, S36).

Figure S9. The authors mention they detect AIR as compound 86 and HMP-P as compound 87. I think these are dephosphorylated compounds but the structure of 86 is never provided (or I could

not find it) and the structure of 87 does not show up until Figure S47. I suggest to draw them in Figure S9 in the same way that the authors provide the structure of compound 88 in Figure S12. This is important given how often 86 and 87 is mentioned in the SI figures.

Supporting Information, Page S11: We did report the structure of **86** and **87** on page S11 but did not caption the figure. We regret the inconvenience caused. The figure on page S11 is now captioned as Figure S2 and it has been cited in the main paper on page 6, paragraph 1.

Figure S29. The WT reaction seems to produce one peak at ~15 min that is neither in the noenzyme control nor in the C475S reaction. Is this peak reproducibly observed? What is its mass?

Supporting Information, Page S34, Figure S30: The peak at ~15 min in wt *Cc*ThiC is 2'deoxyguanosine that comes bound with the purified wt ThiC. The *Cc*ThiC (C474S) variant also purifies with bound 2'-deoxyguanosine but the levels are significantly lower.

Guanosine and adenosine are also bound to the purified wt ThiC. The binding site and the reason for binding of these nucleosides to wt ThiC is not clear at this point.

Additional Questions:

Quality of experimental data, technical rigor: Top 1%

Significance to chemistry researchers in this and related fields: Top 5%

Broad interest to other researchers: Top 5%

Novelty: Top 1%

Is this research study suitable for media coverage or a First Reactions (a News & Views piece in the journal)?: No
